# Supplementary material for: TREM2 Alleviates Neuroinflammation by Maintaining Cellular Metabolic Homeostasis and Mitophagy Activity During Early Inflammation
Source: Diseases. 2025 Feb 16;13(2):60. doi: 10.3390/diseases13020060 (PMC11854088; doi:10.3390/diseases13020060)
Supplement: Supplementary file 1 [file diseases-13-00060-s001.zip › figure s1.pdf]

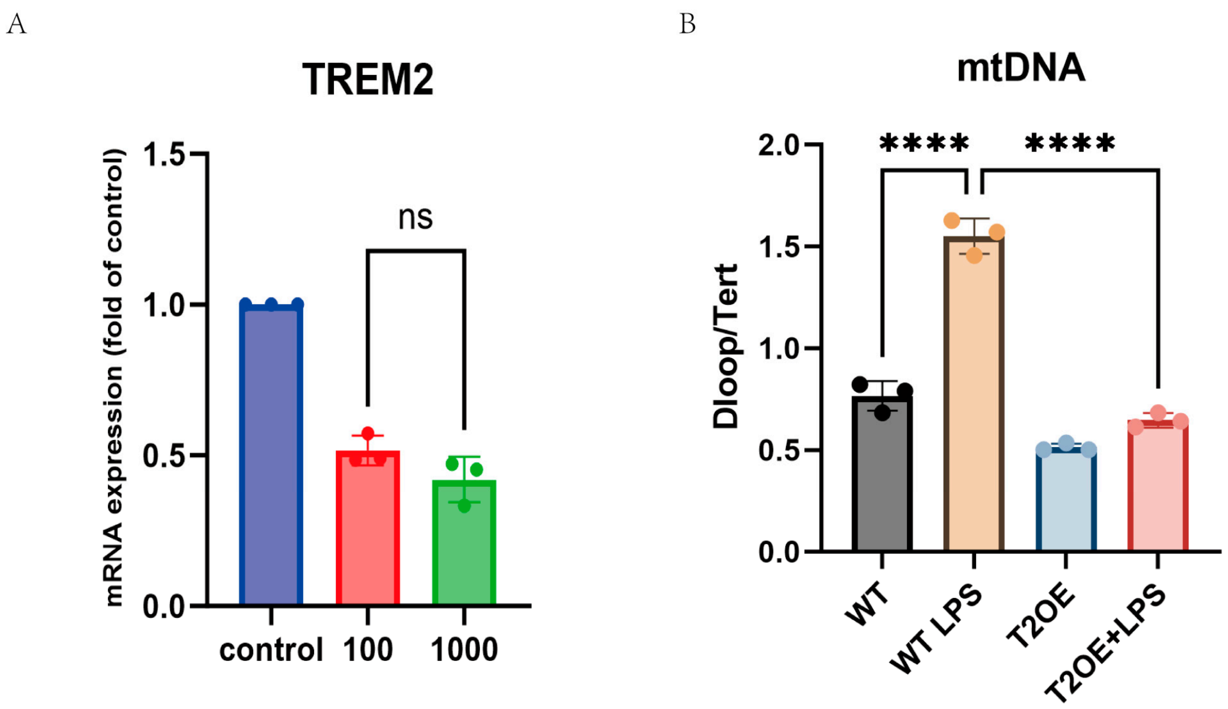

Figure S1. Expression levels of the TREM2 gene in wild-type BV-2 cells treated with different concentrations of LPS for 4 hours (A). RT−qPCR was used to quantify the mRNA expression of the TREM2 gene (n = 3). The control group consisted of untreated wild-type BV-2 cells. mtDNA release in TREM2-overexpressing and wild-type cells following LPS treatment for 4 hours (B). qPCR was used to determine the ratio of mitochondrial genes (Dloop) to nuclear genes (Tert) (n = 3). The results presented are representative of three independent experiments. ns: not significant , ****p<0.0001.
