# Supplementary material for: TREM2 Alleviates Neuroinflammation by Maintaining Cellular Metabolic Homeostasis and Mitophagy Activity During Early Inflammation
Source: Diseases. 2025 Feb 16;13(2):60. doi: 10.3390/diseases13020060 (PMC11854088; doi:10.3390/diseases13020060)
Supplement: Supplementary file 1 [file diseases-13-00060-s001.zip › Western blot showed in the article.pdf]

the quantitative data for these images can now be found in the supplementary file

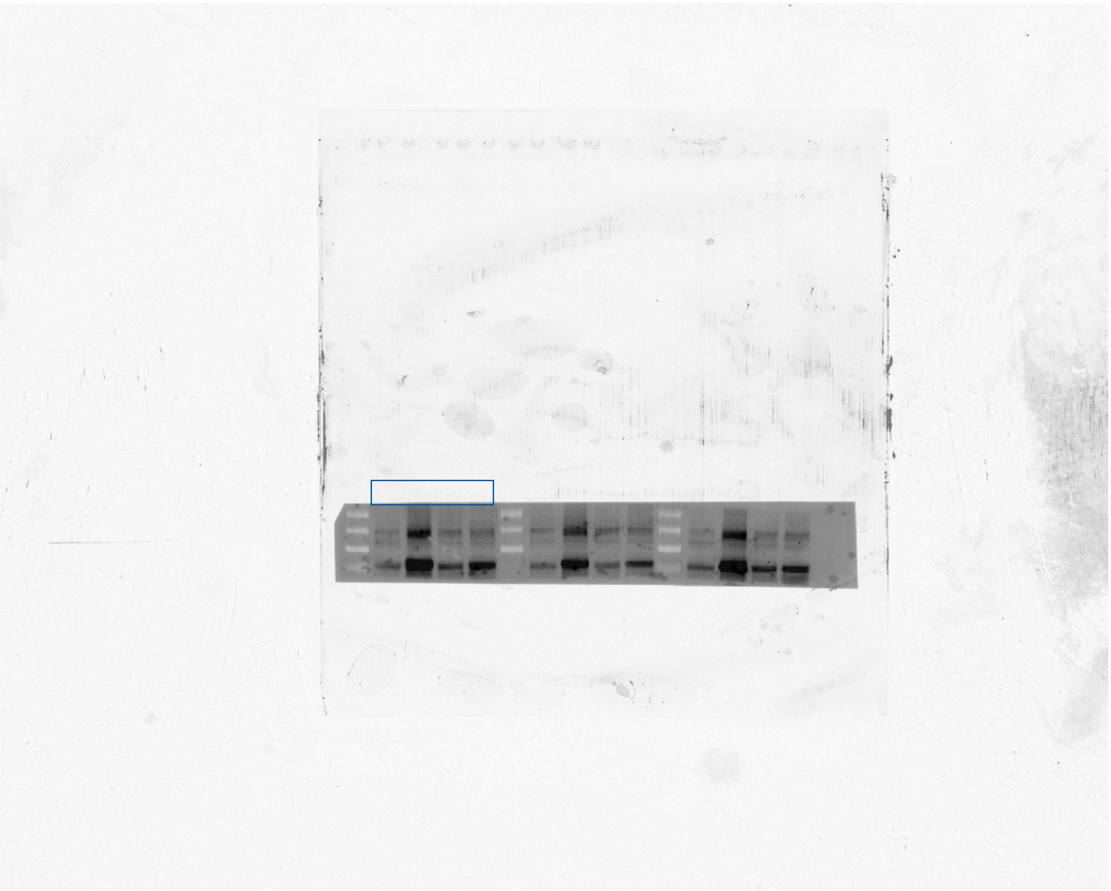

The region within the blue box represents the iNOS blot displayed in the manuscript.

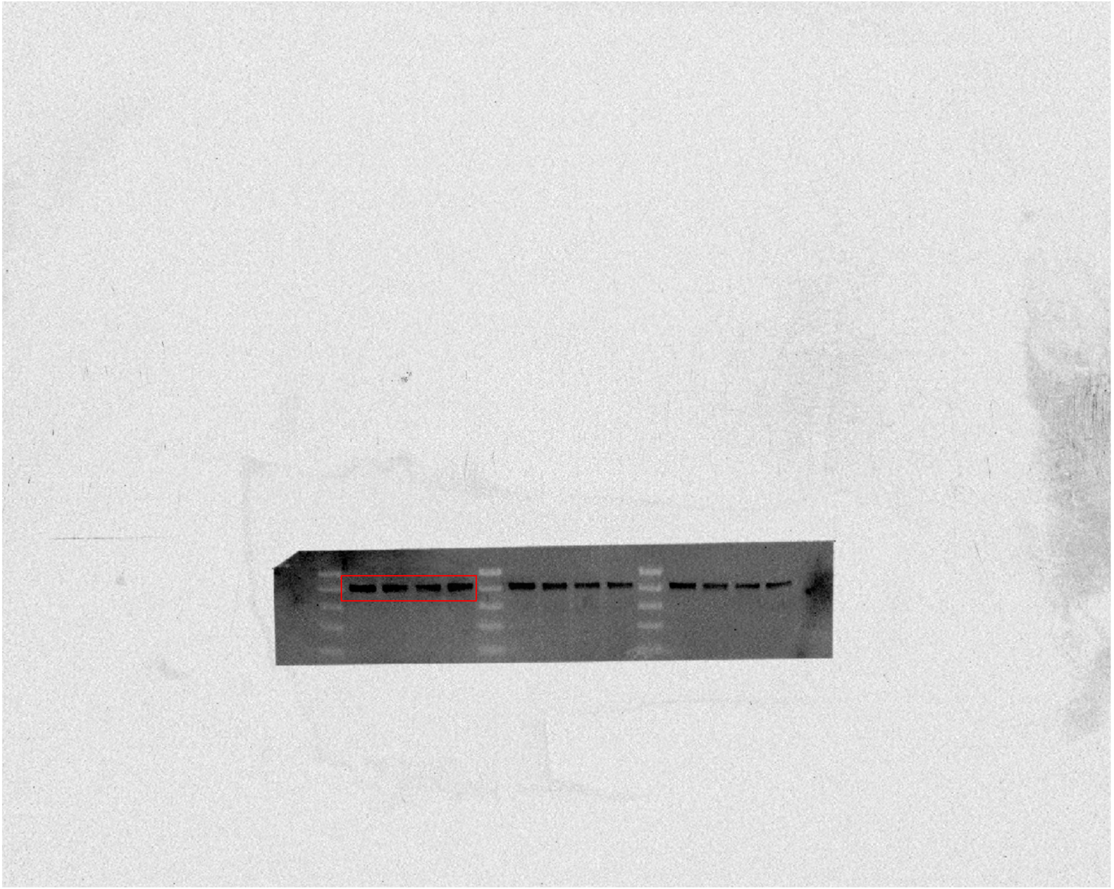

The region within the red box represents the ACTB blot corresponding to iNOS displayed in the manuscript.

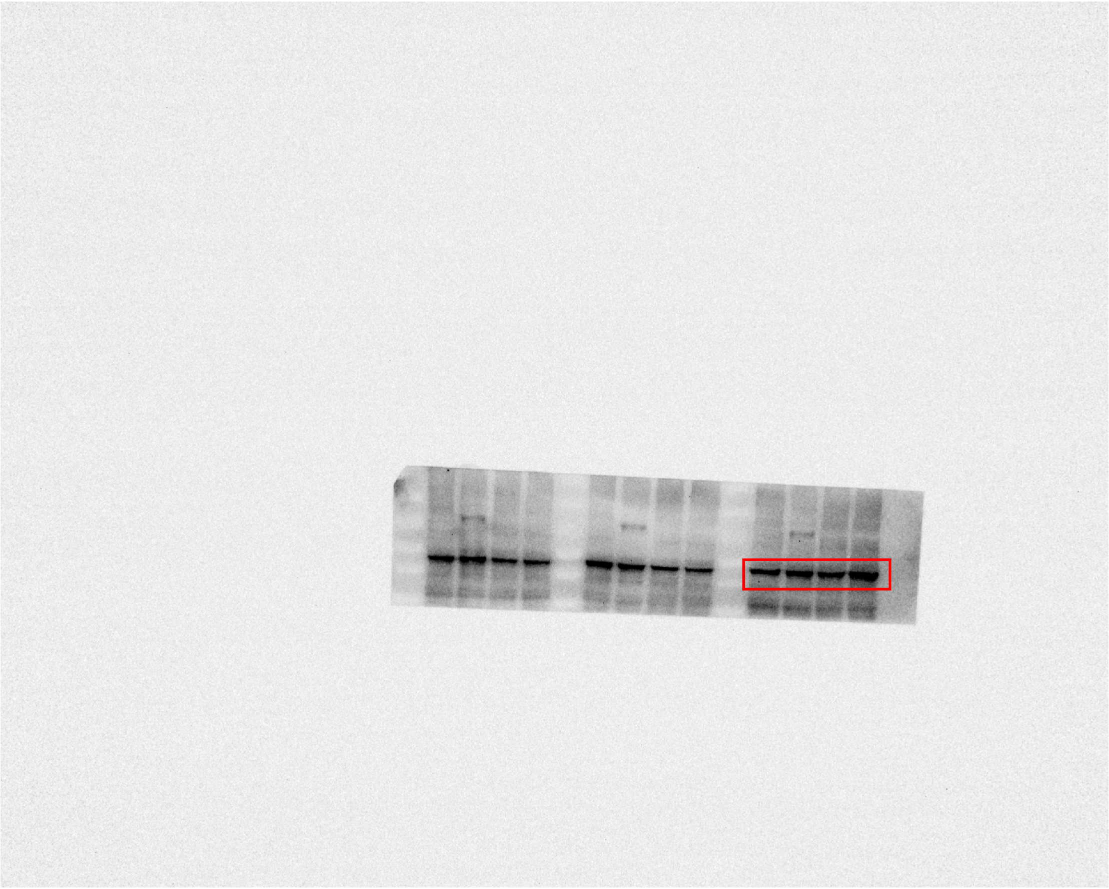

The region within the red box represents the ACTB blot corresponding to iNOS displayed in the manuscript. After iNOS exposure, the membrane was stripped and re-probed for ACTB.

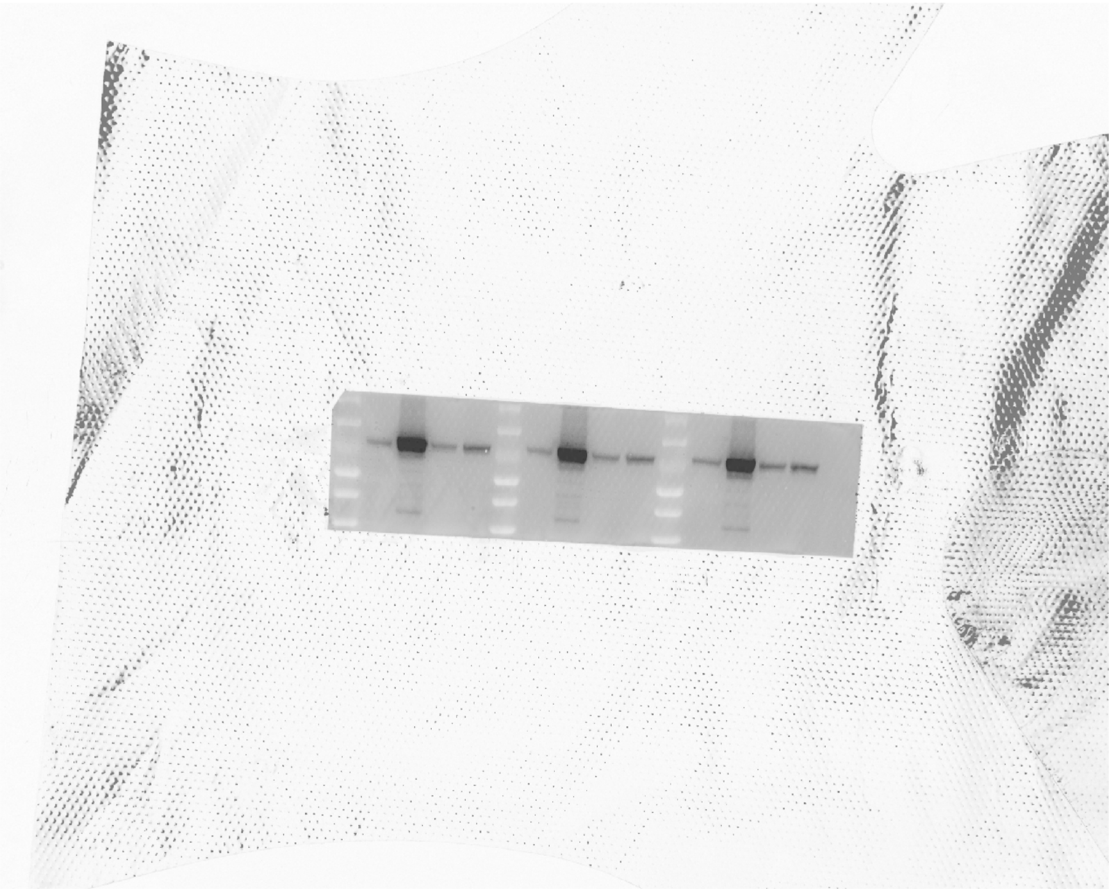

The region within the blue box represents the COX2 blot displayed in the manuscript.

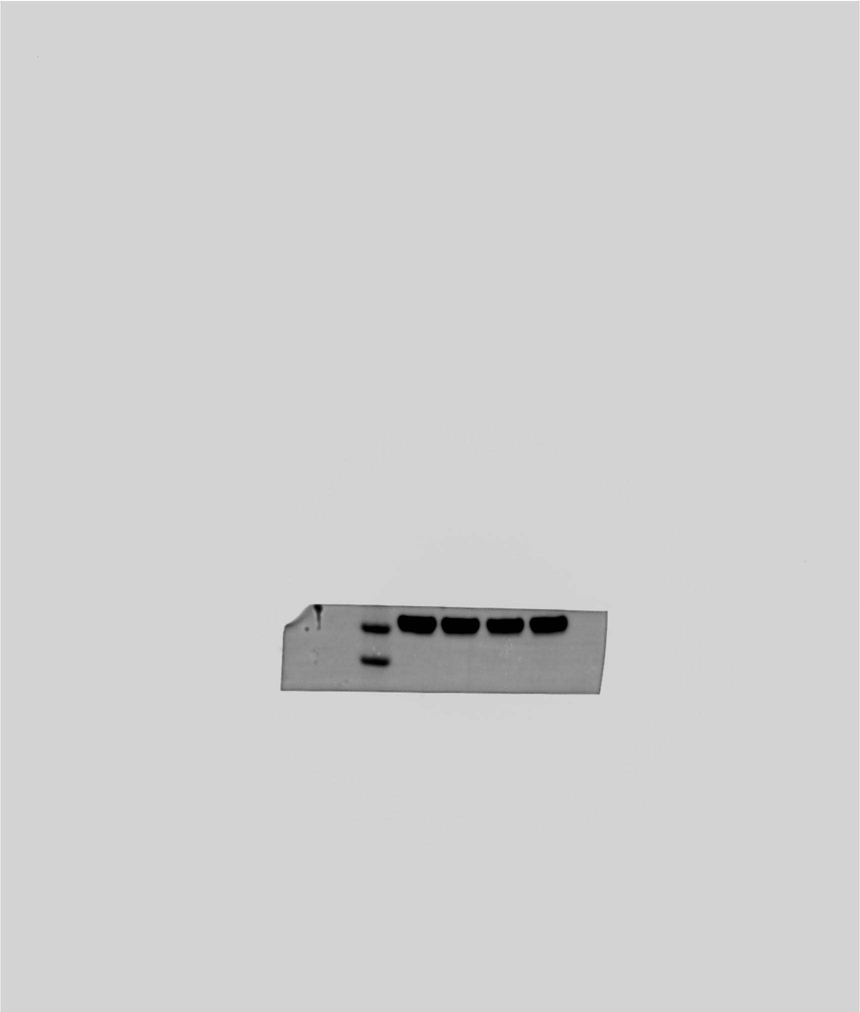

The region within the red box represents the ACTB blot corresponding to p62 and LC3 displayed in the manuscript.

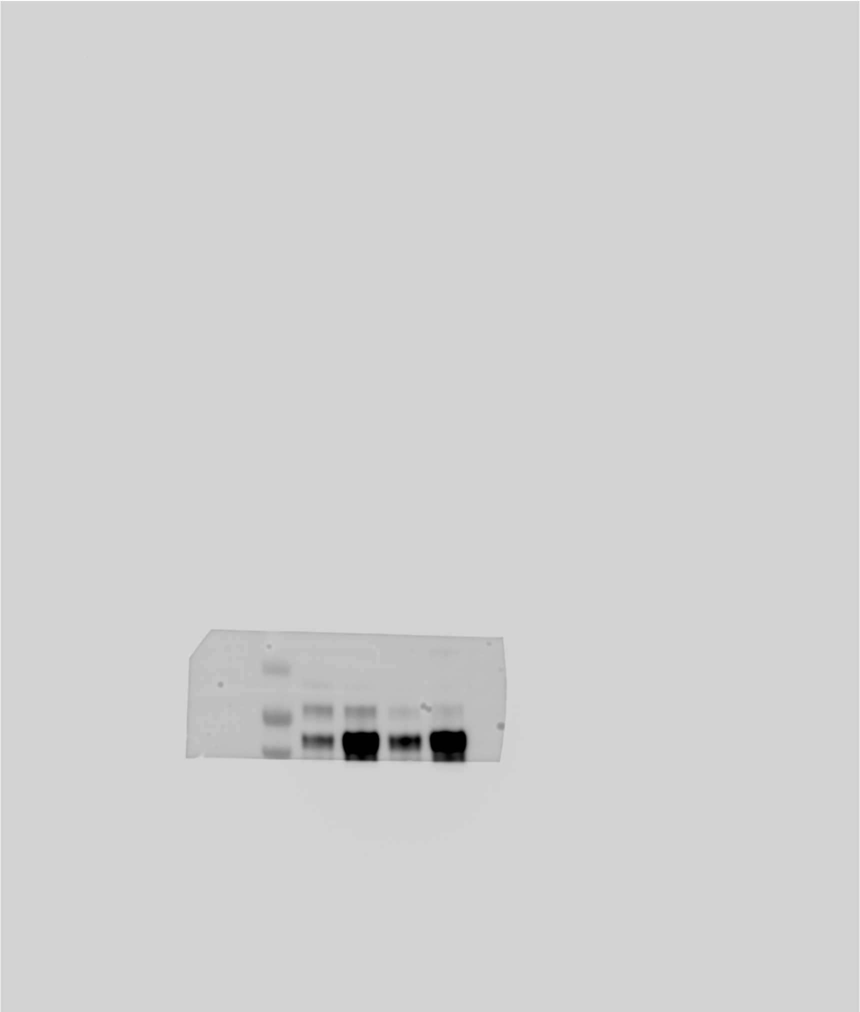

The region within the blue box represents the p62 blot displayed in the manuscript.

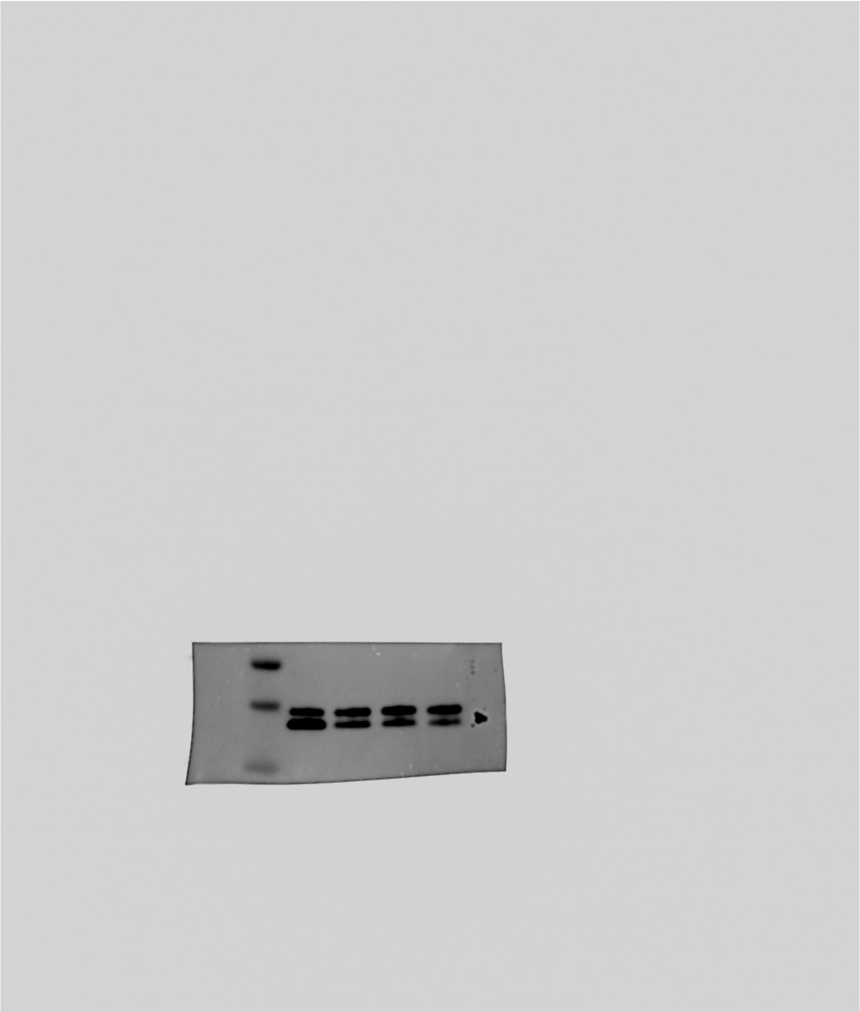

The region within the blue box represents the LC3 blot displayed in the manuscript.
