# Supplementary figures and images for: TREM2 Alleviates Neuroinflammation by Maintaining Cellular Metabolic Homeostasis and Mitophagy Activity During Early Inflammation
Source: Diseases. 2025 Feb 16;13(2):60. doi: 10.3390/diseases13020060 (PMC11854088; doi:10.3390/diseases13020060)

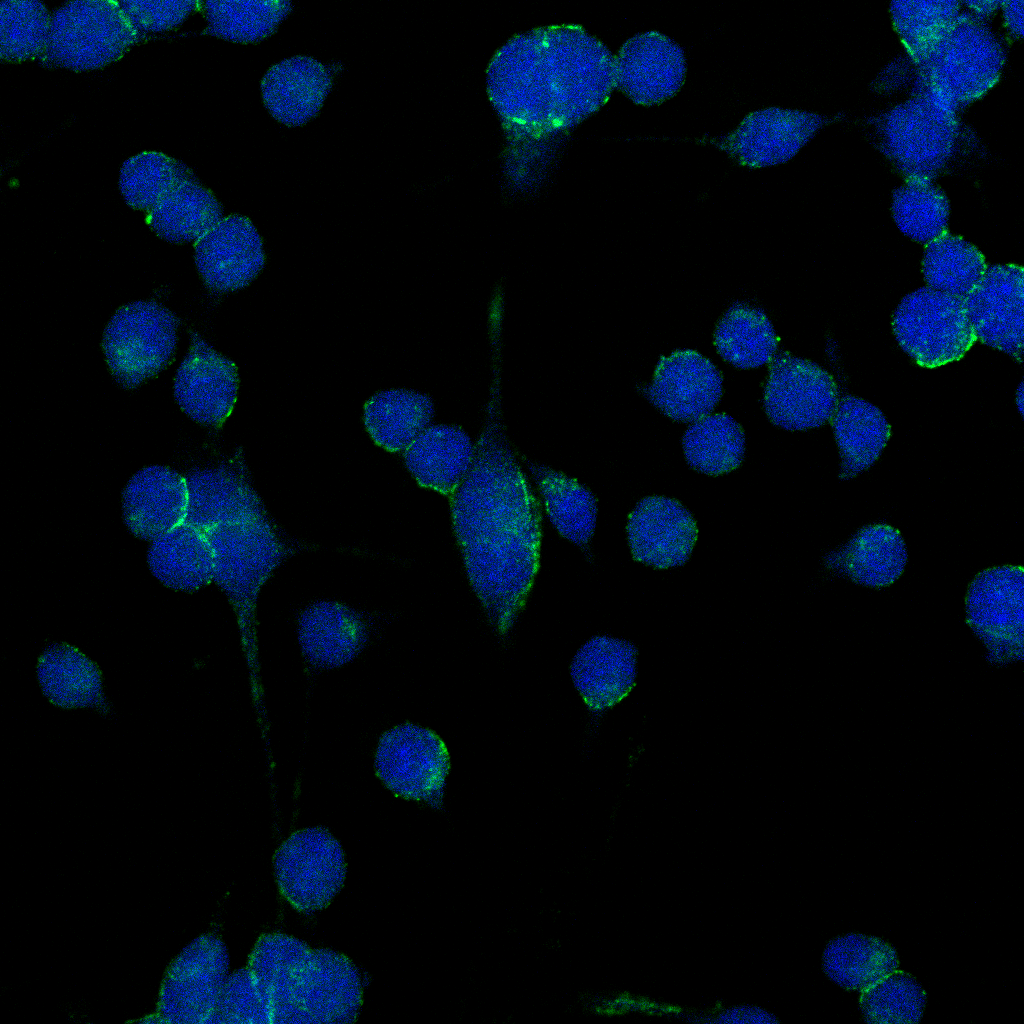

Supplement: Supplementary file 1 [file diseases-13-00060-s001.zip › source data-IF/all IF raw data/OE/OE-1 600-1.tif]

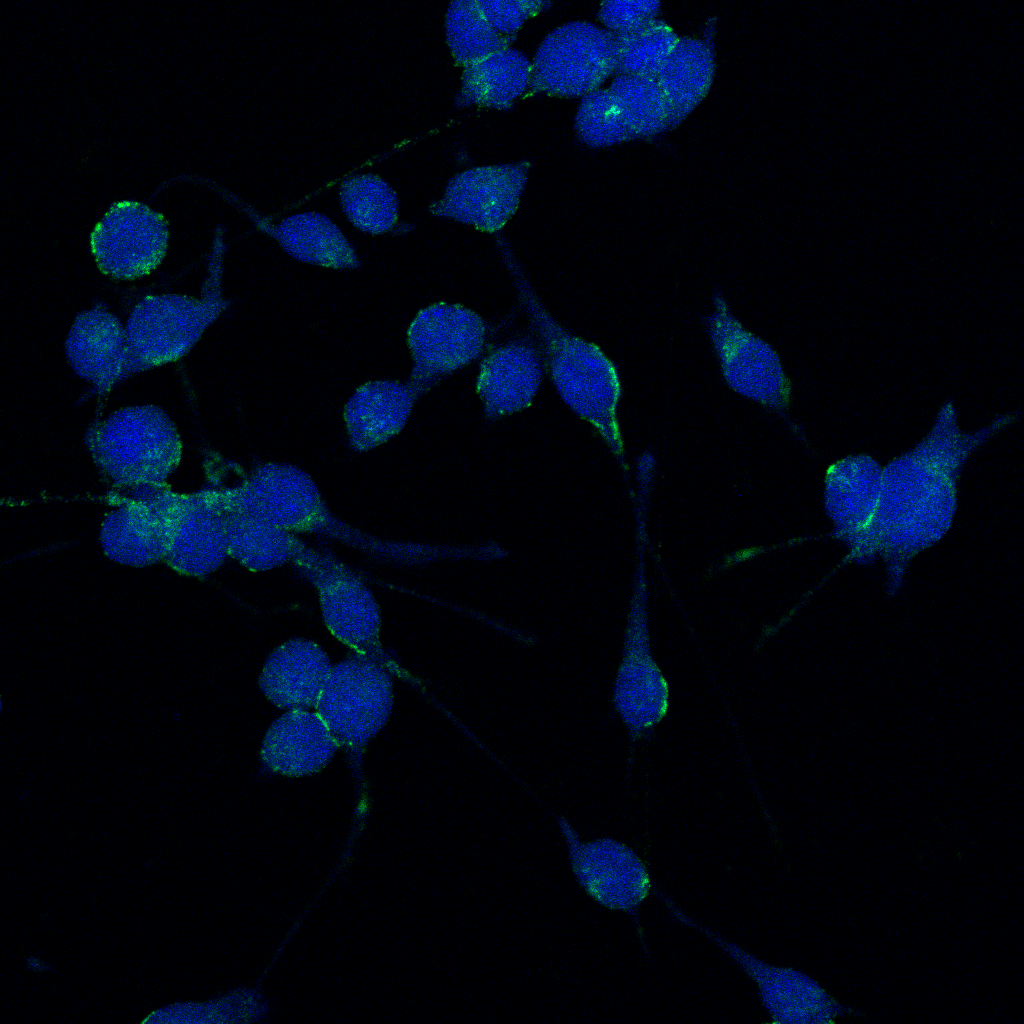

Supplement: Supplementary file 1 [file diseases-13-00060-s001.zip › source data-IF/all IF raw data/OE/OE-1 600-2.tif]

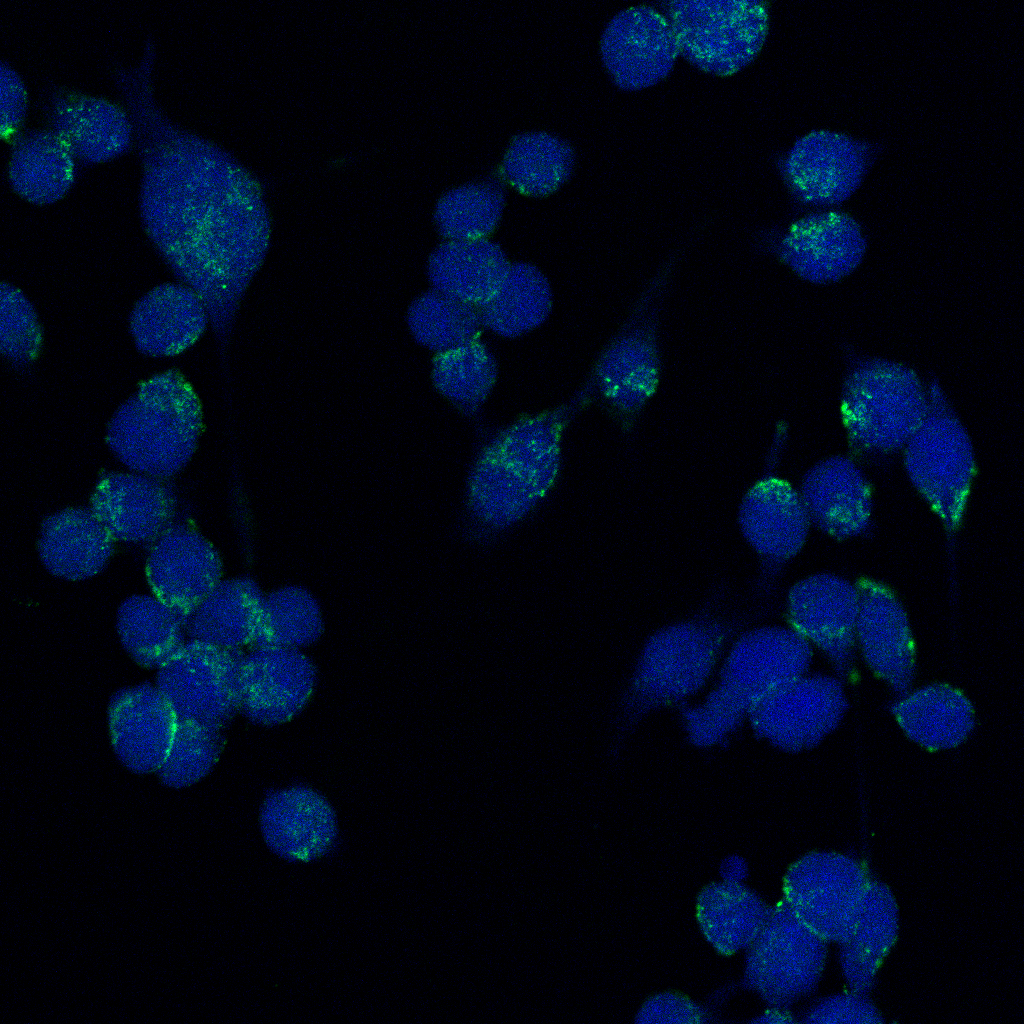

Supplement: Supplementary file 1 [file diseases-13-00060-s001.zip › source data-IF/all IF raw data/OE/OE-1 600-3.tif]

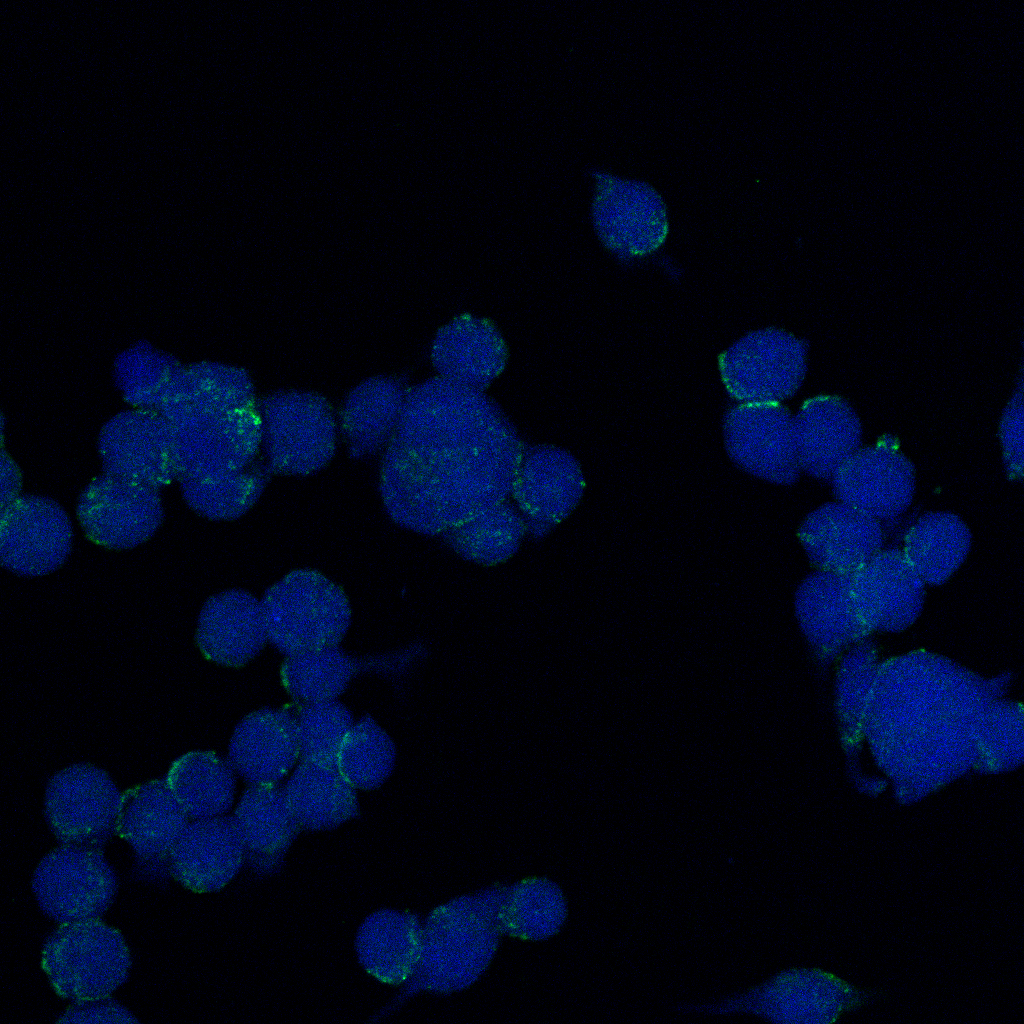

Supplement: Supplementary file 1 [file diseases-13-00060-s001.zip › source data-IF/all IF raw data/OE/OE-1 600-4.tif]

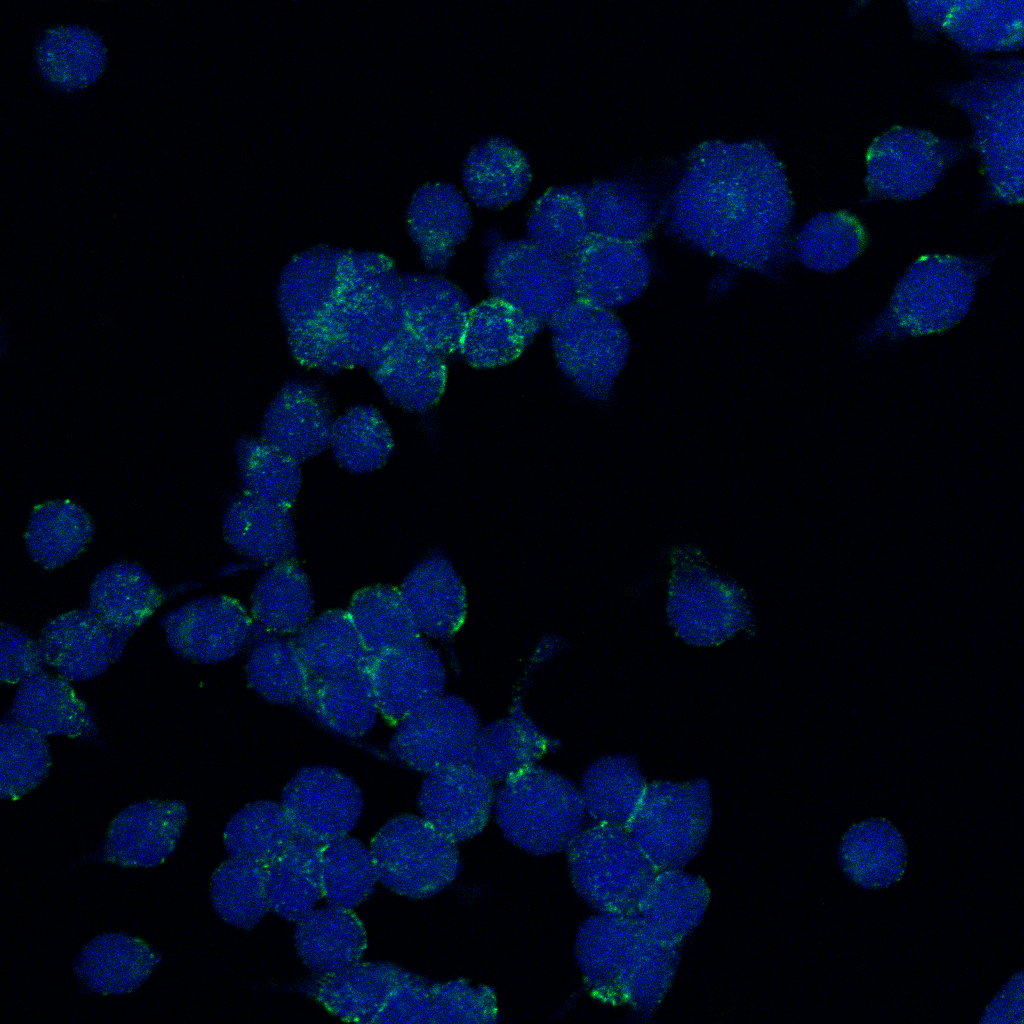

Supplement: Supplementary file 1 [file diseases-13-00060-s001.zip › source data-IF/all IF raw data/OE/OE-1 600-5.tif]

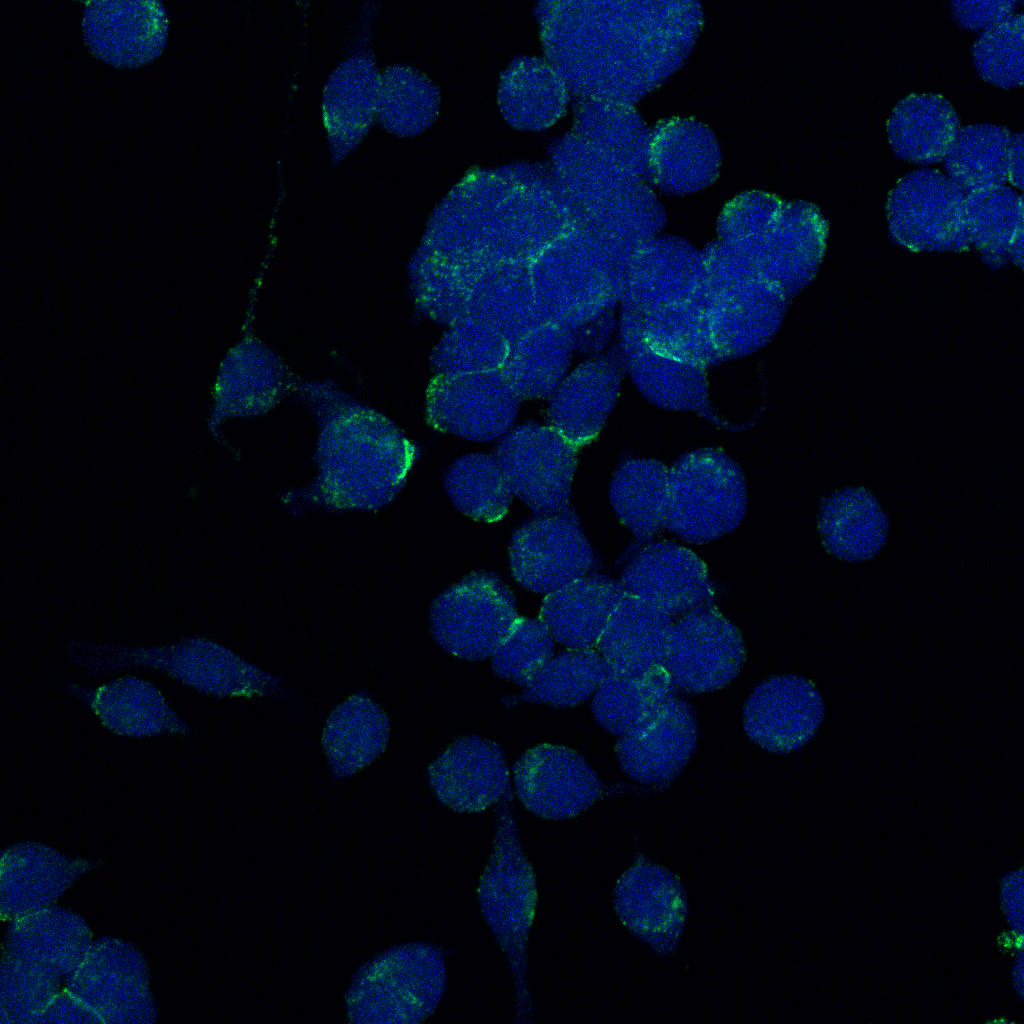

Supplement: Supplementary file 1 [file diseases-13-00060-s001.zip › source data-IF/all IF raw data/OE/OE-1 600-6.tif]

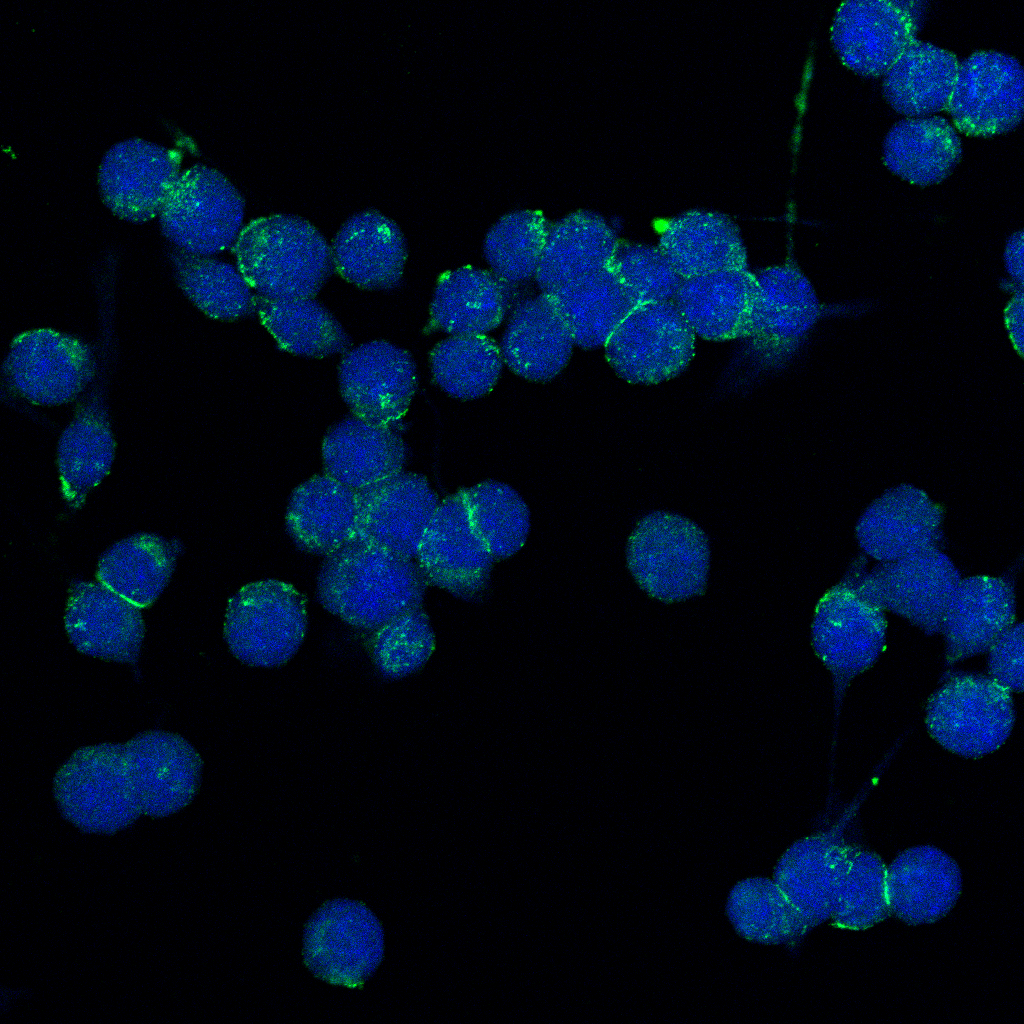

Supplement: Supplementary file 1 [file diseases-13-00060-s001.zip › source data-IF/all IF raw data/OE/OE-2 600-1.tif]

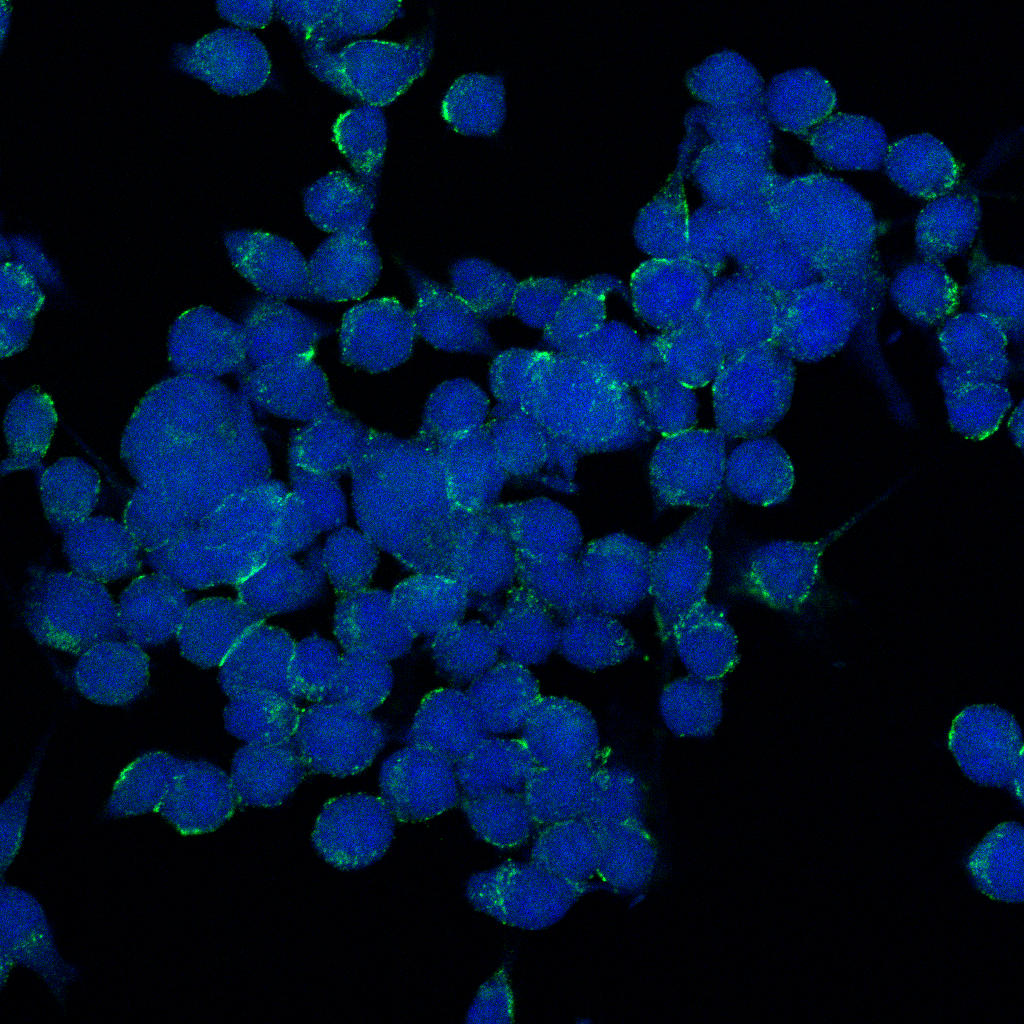

Supplement: Supplementary file 1 [file diseases-13-00060-s001.zip › source data-IF/all IF raw data/OE/OE-2 600-2.tif]

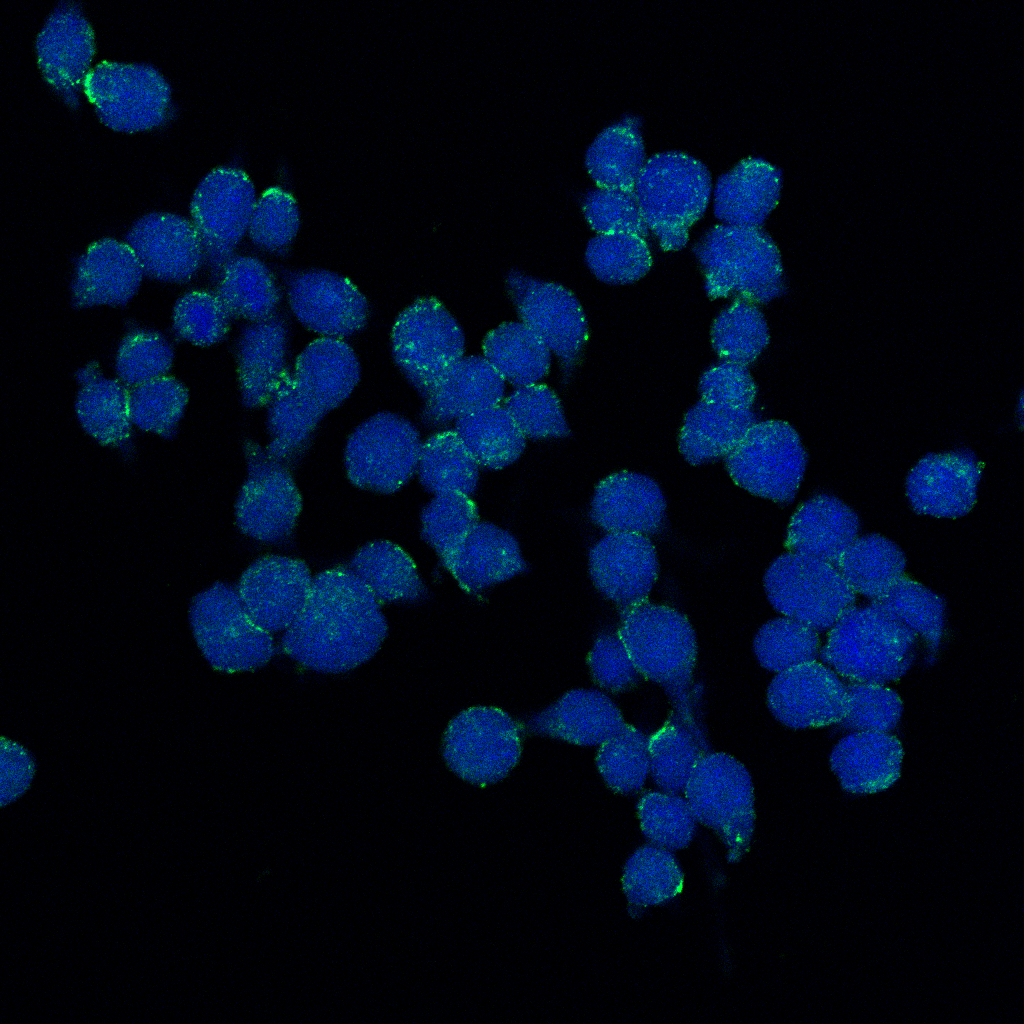

Supplement: Supplementary file 1 [file diseases-13-00060-s001.zip › source data-IF/all IF raw data/OE/OE-2 600-3.tif]

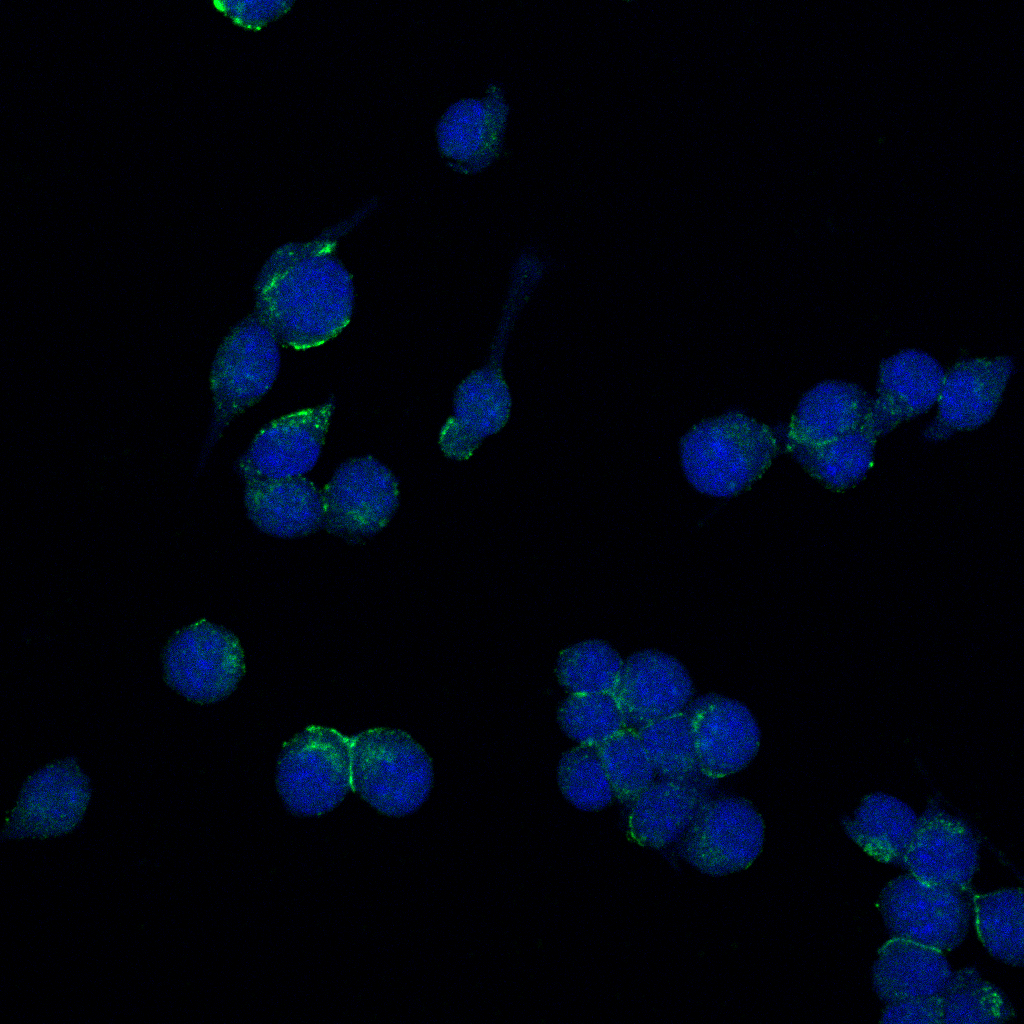

Supplement: Supplementary file 1 [file diseases-13-00060-s001.zip › source data-IF/all IF raw data/OE/OE-2 600-4.tif]

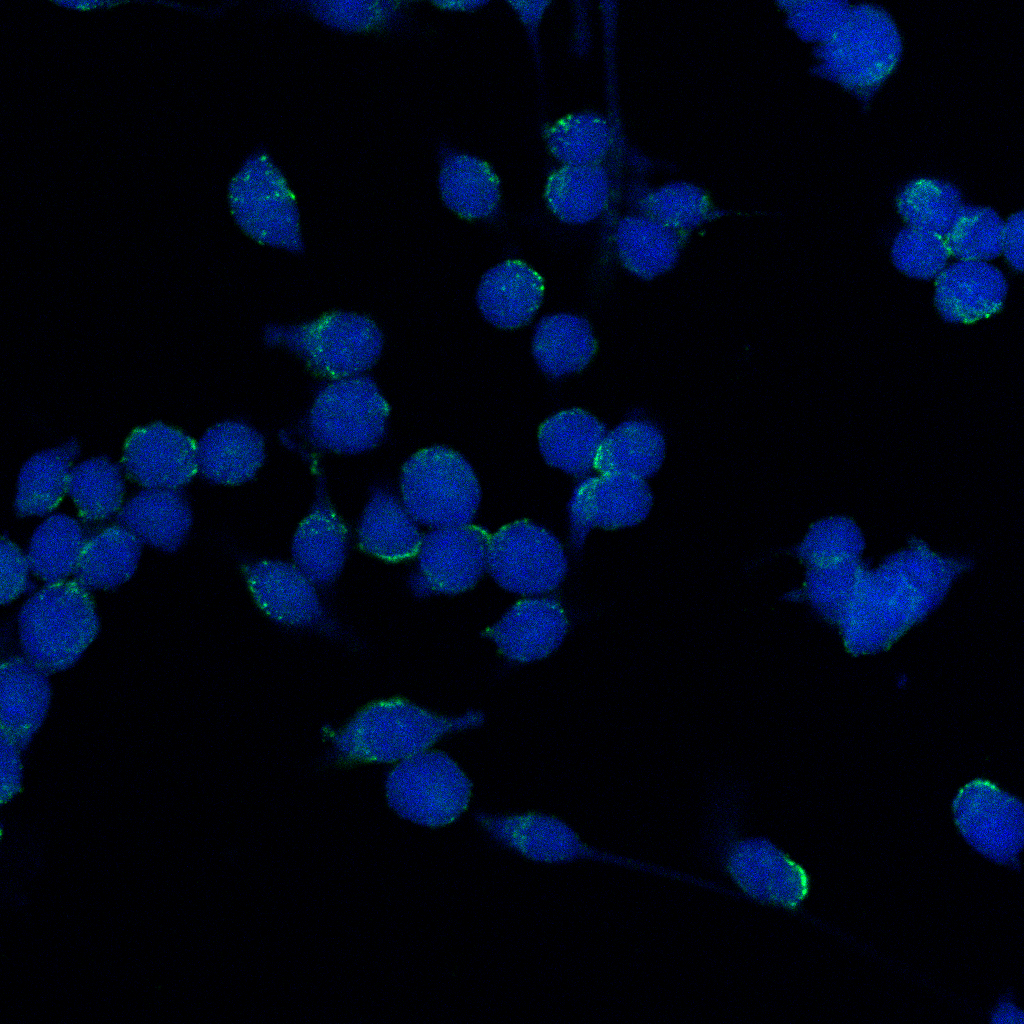

Supplement: Supplementary file 1 [file diseases-13-00060-s001.zip › source data-IF/all IF raw data/OE/OE-2 600-5.tif]

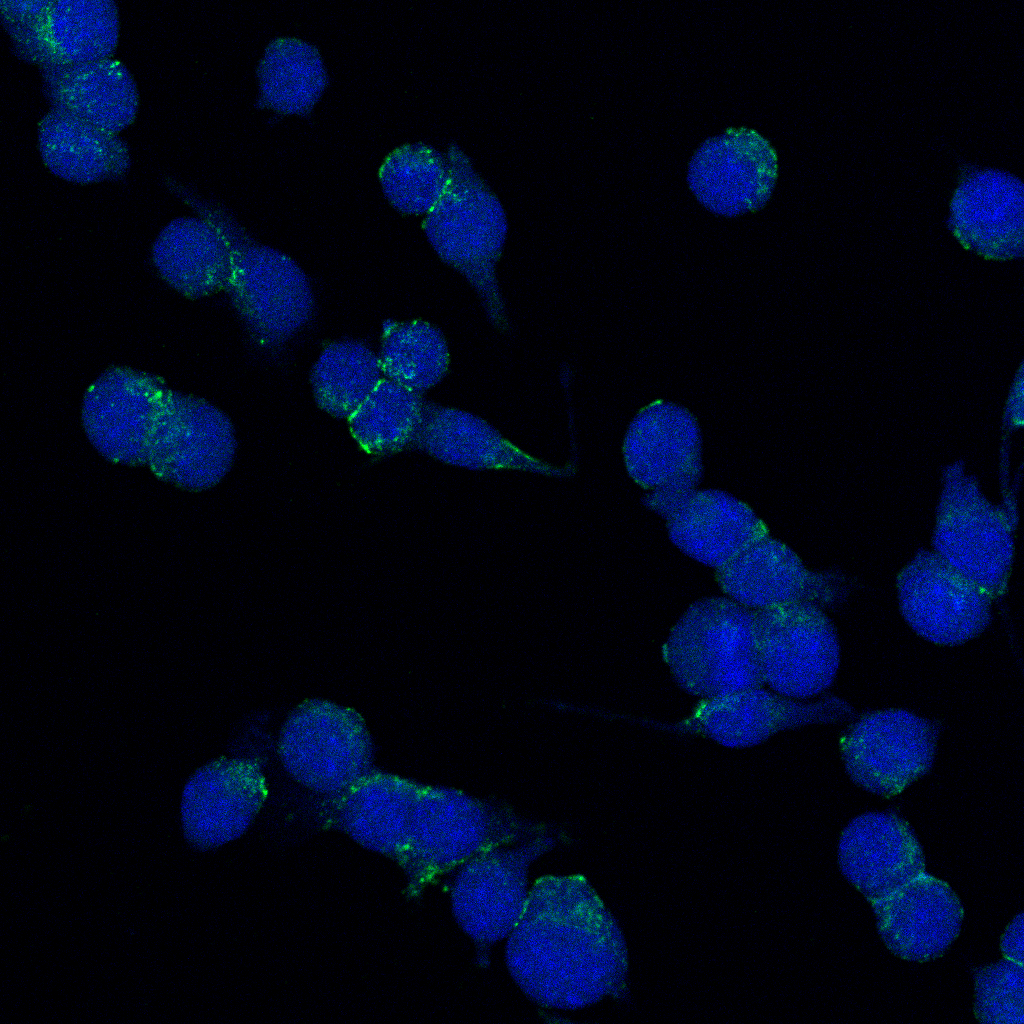

Supplement: Supplementary file 1 [file diseases-13-00060-s001.zip › source data-IF/all IF raw data/OE/OE-2 600-6.tif]

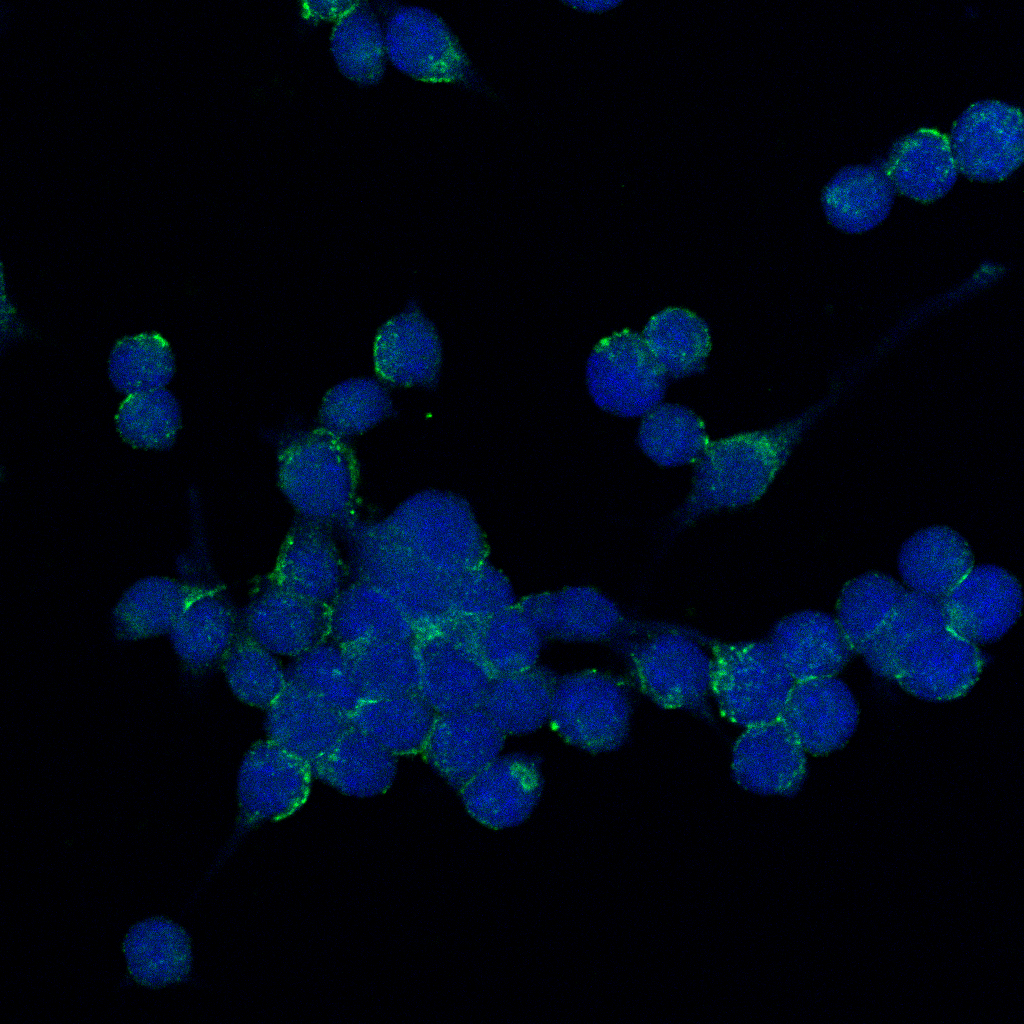

Supplement: Supplementary file 1 [file diseases-13-00060-s001.zip › source data-IF/all IF raw data/OE/OE-3 600-1.tif]

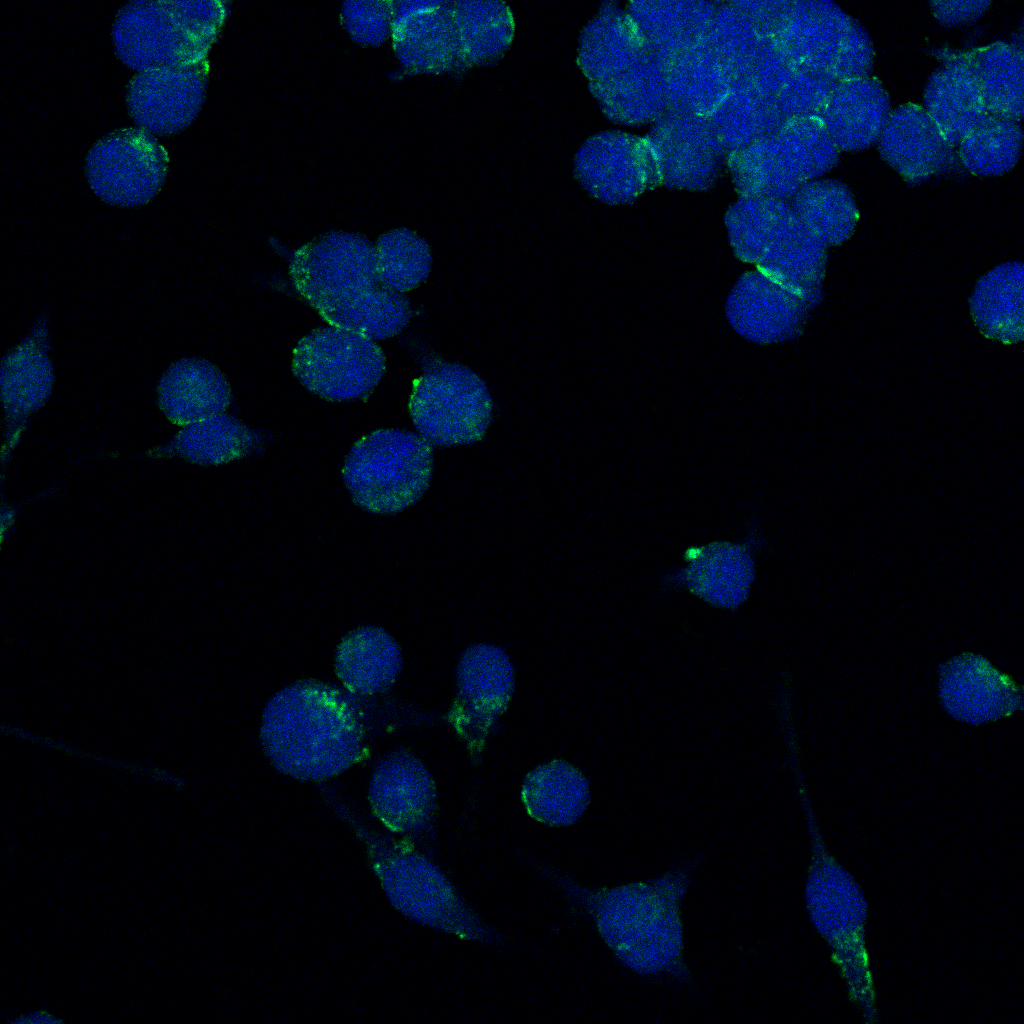

Supplement: Supplementary file 1 [file diseases-13-00060-s001.zip › source data-IF/all IF raw data/OE/OE-3 600-2.tif]

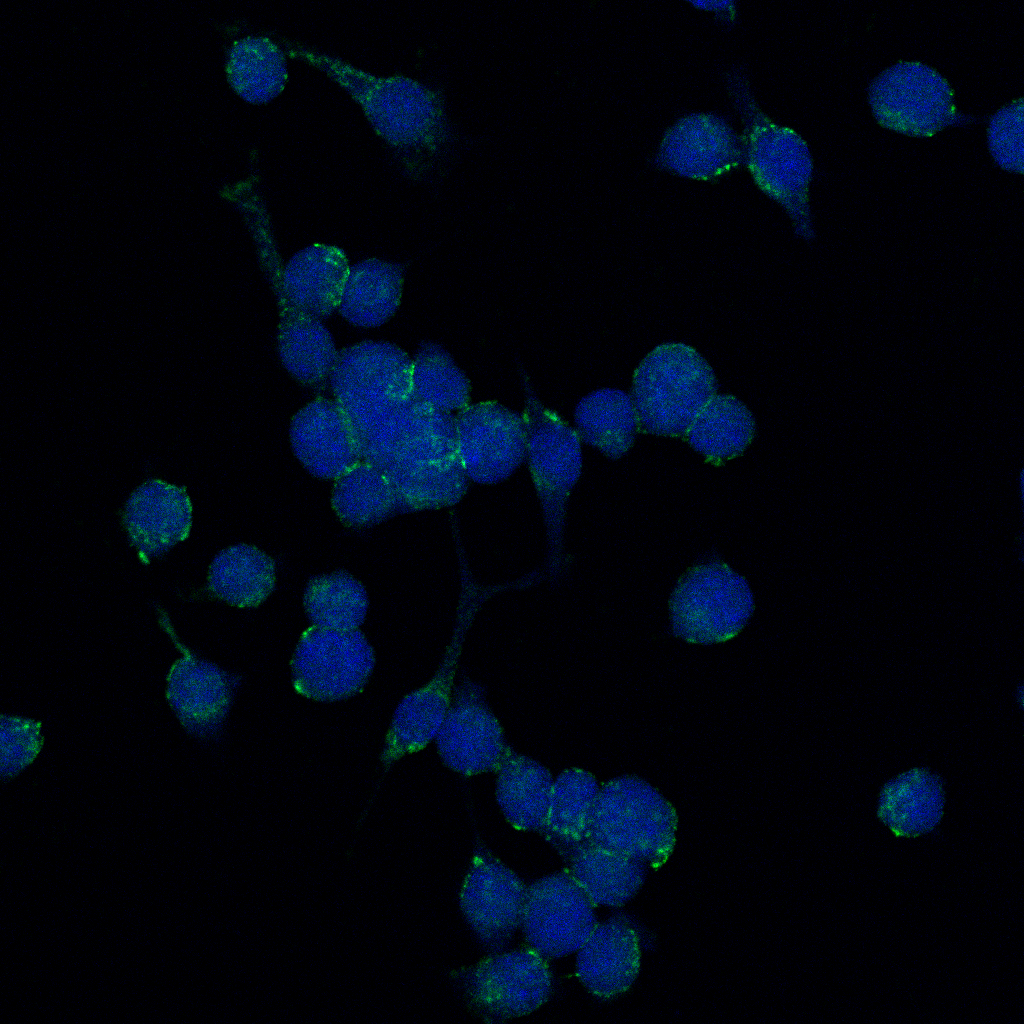

Supplement: Supplementary file 1 [file diseases-13-00060-s001.zip › source data-IF/all IF raw data/OE/OE-3 600-3.tif]

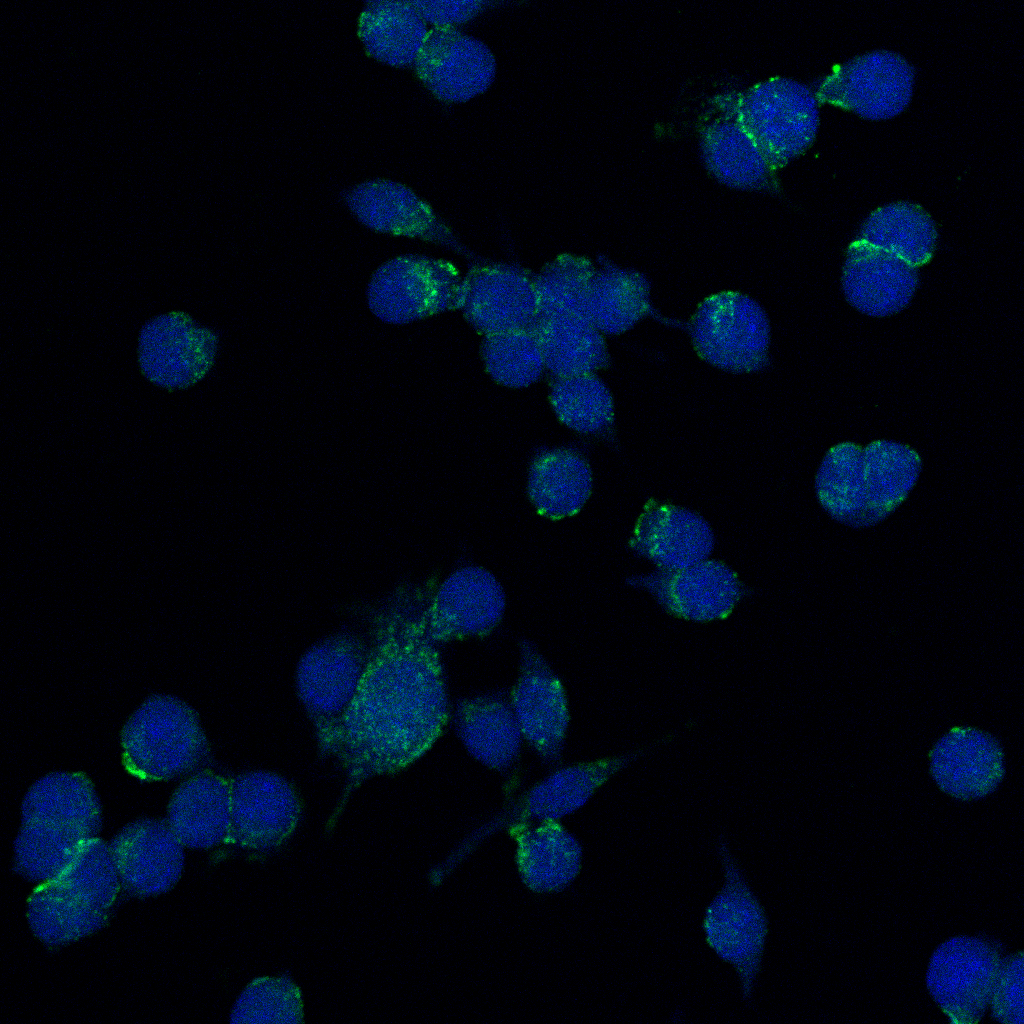

Supplement: Supplementary file 1 [file diseases-13-00060-s001.zip › source data-IF/all IF raw data/OE/OE-3 600-4.tif]

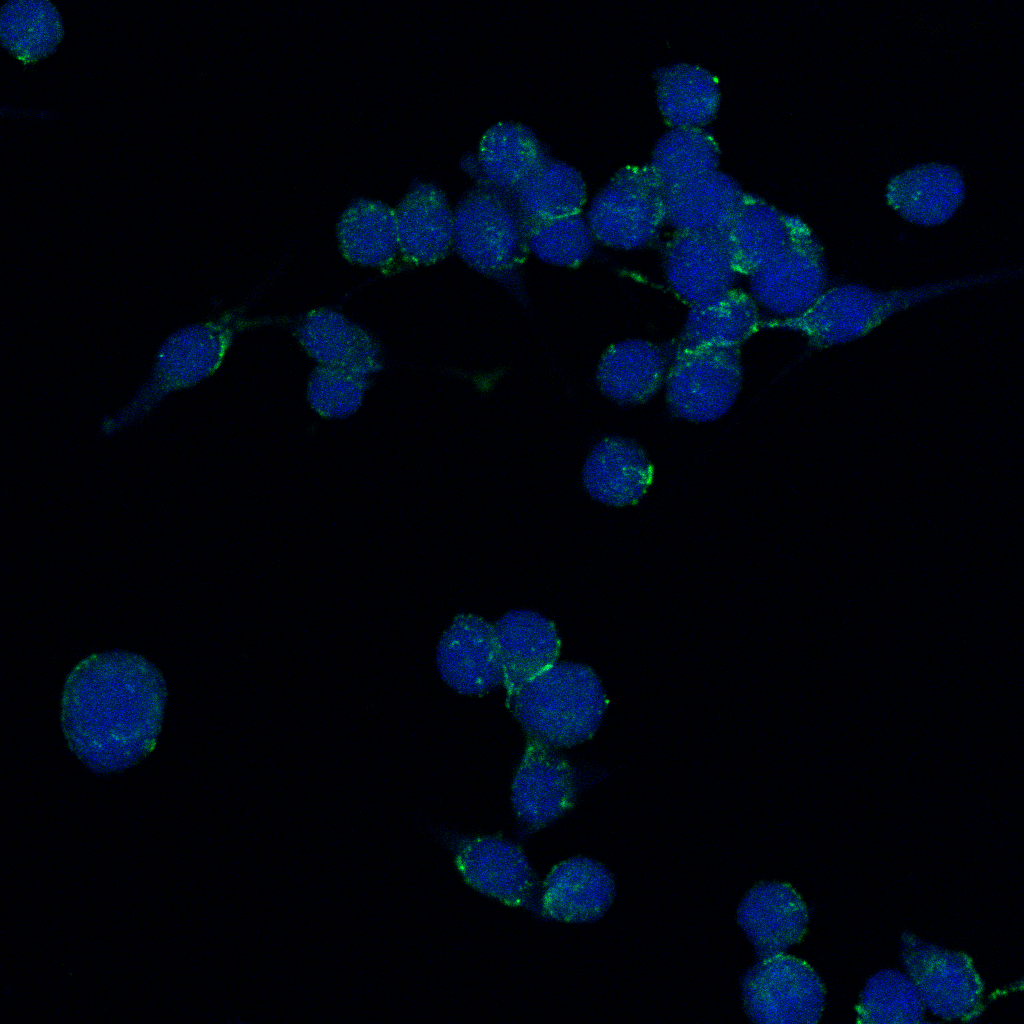

Supplement: Supplementary file 1 [file diseases-13-00060-s001.zip › source data-IF/all IF raw data/OE/OE-3 600-5.tif]

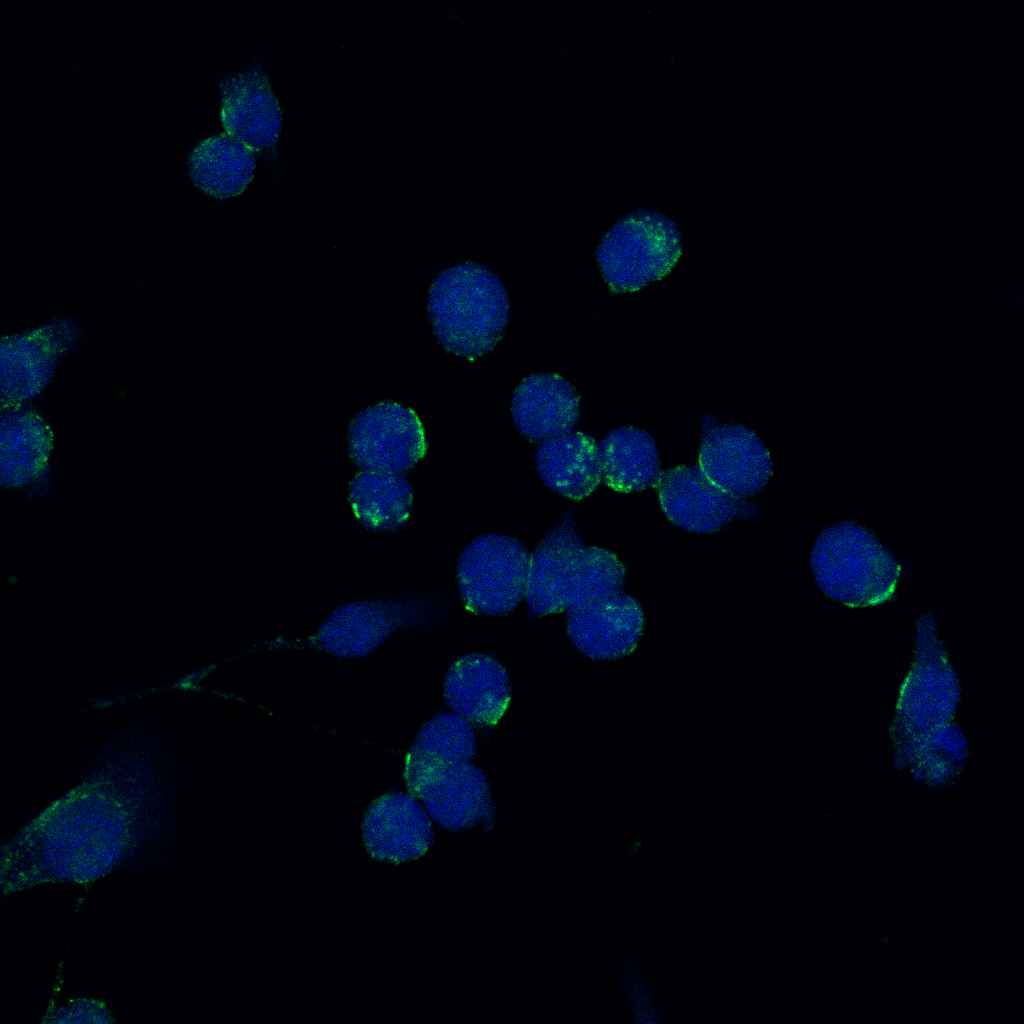

Supplement: Supplementary file 1 [file diseases-13-00060-s001.zip › source data-IF/all IF raw data/OE/OE-3 600-6.tif]

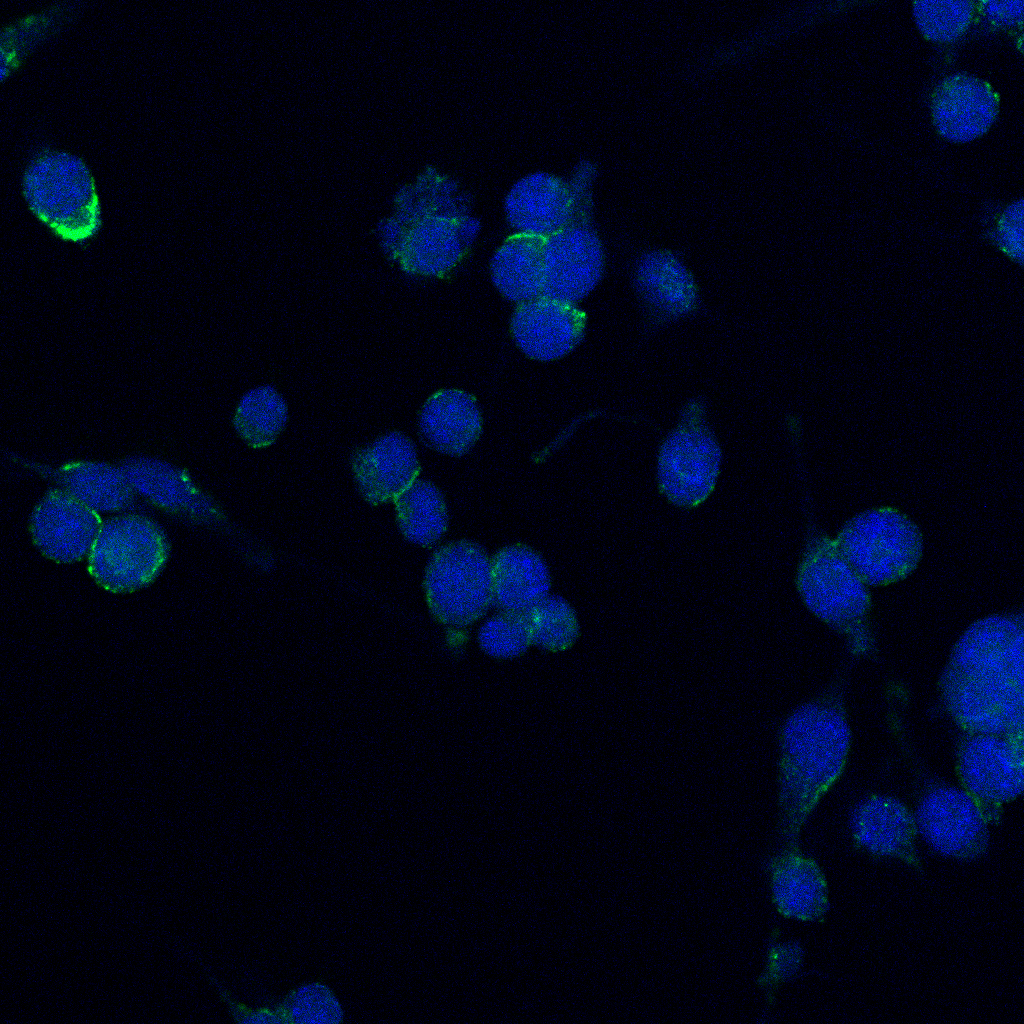

Supplement: Supplementary file 1 [file diseases-13-00060-s001.zip › source data-IF/all IF raw data/OELPS/OE LPS 1 600-1.tif]

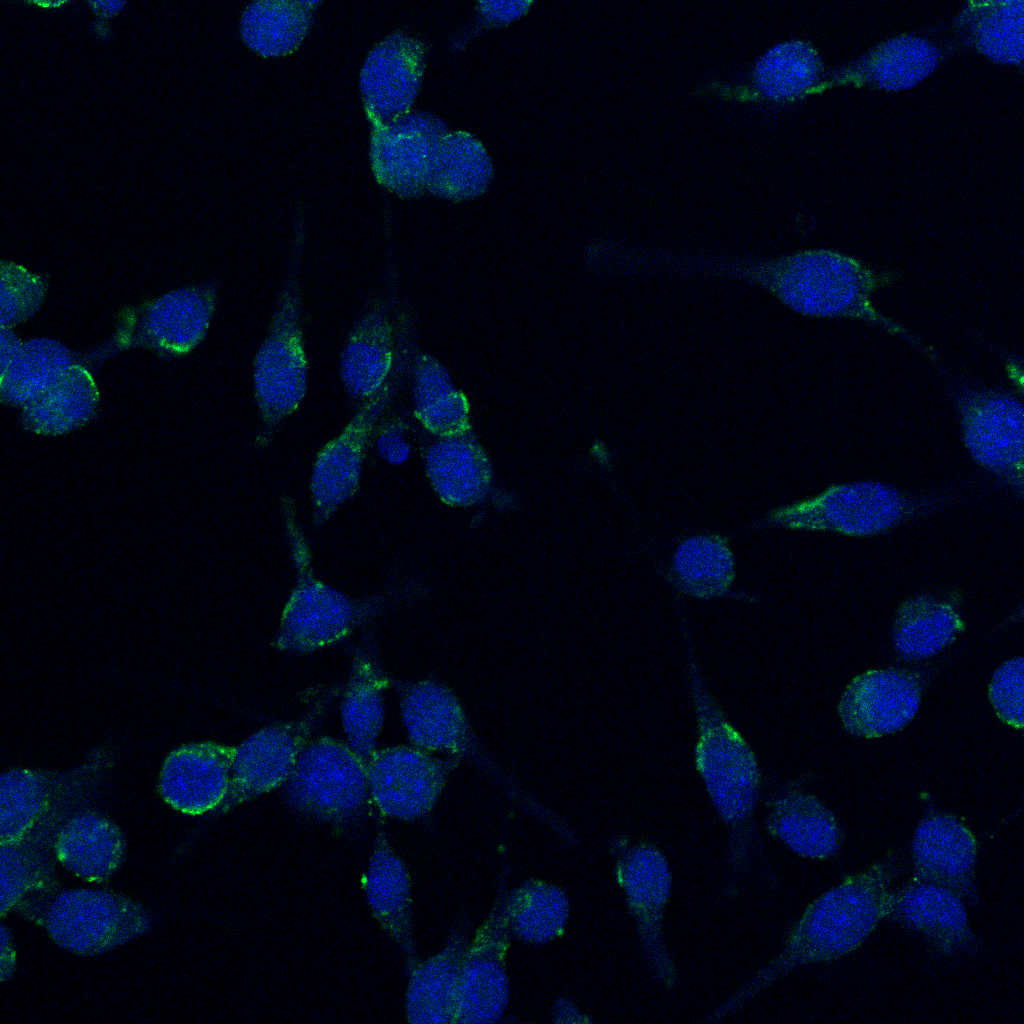

Supplement: Supplementary file 1 [file diseases-13-00060-s001.zip › source data-IF/all IF raw data/OELPS/OE LPS 1 600-2.tif]

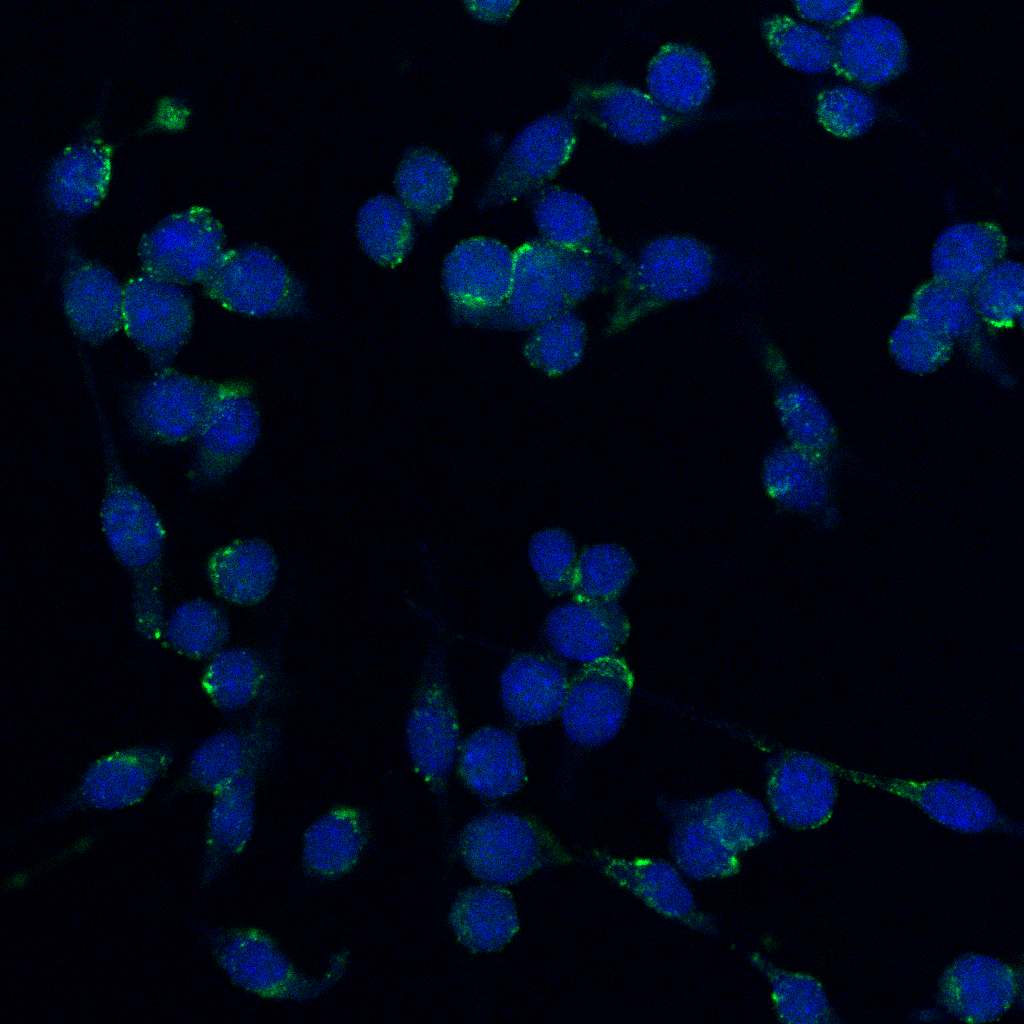

Supplement: Supplementary file 1 [file diseases-13-00060-s001.zip › source data-IF/all IF raw data/OELPS/OE LPS 1 600-3.tif]

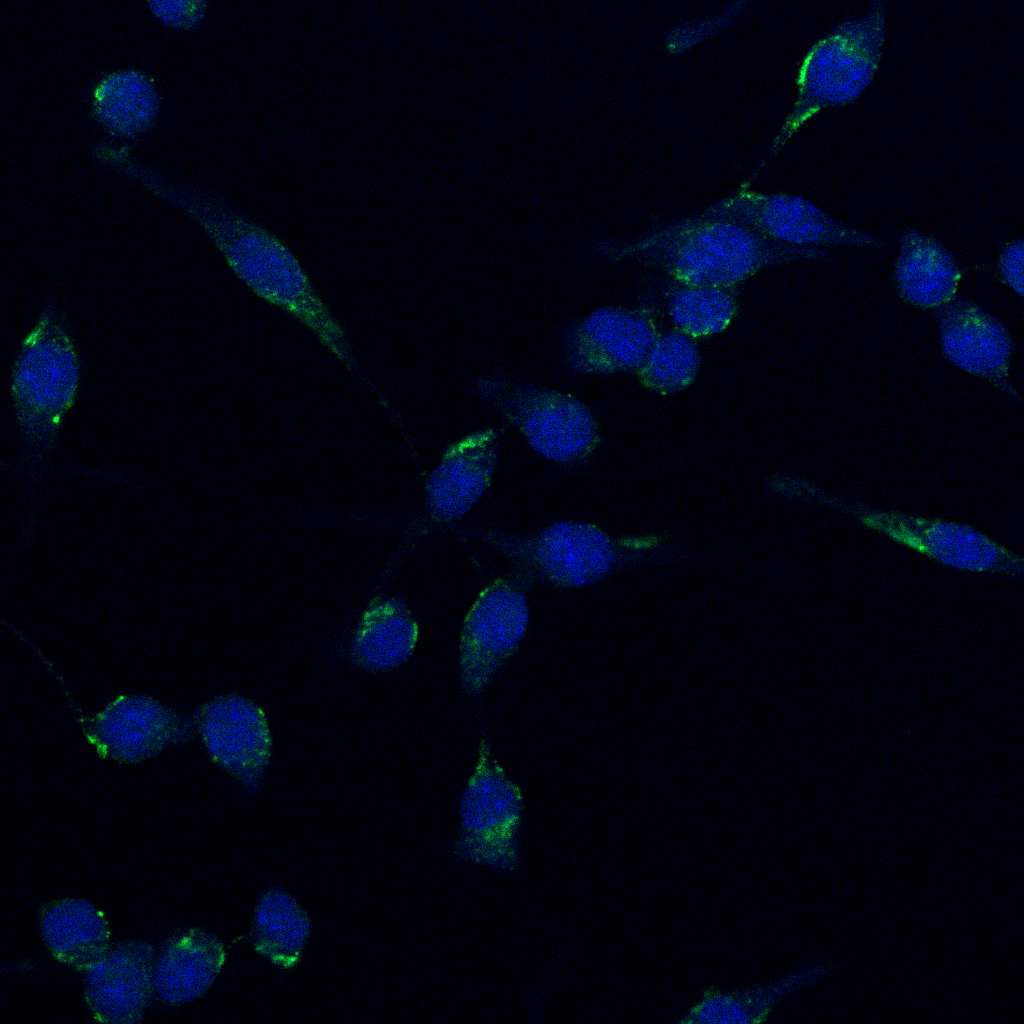

Supplement: Supplementary file 1 [file diseases-13-00060-s001.zip › source data-IF/all IF raw data/OELPS/OE LPS 1 600-4.tif]

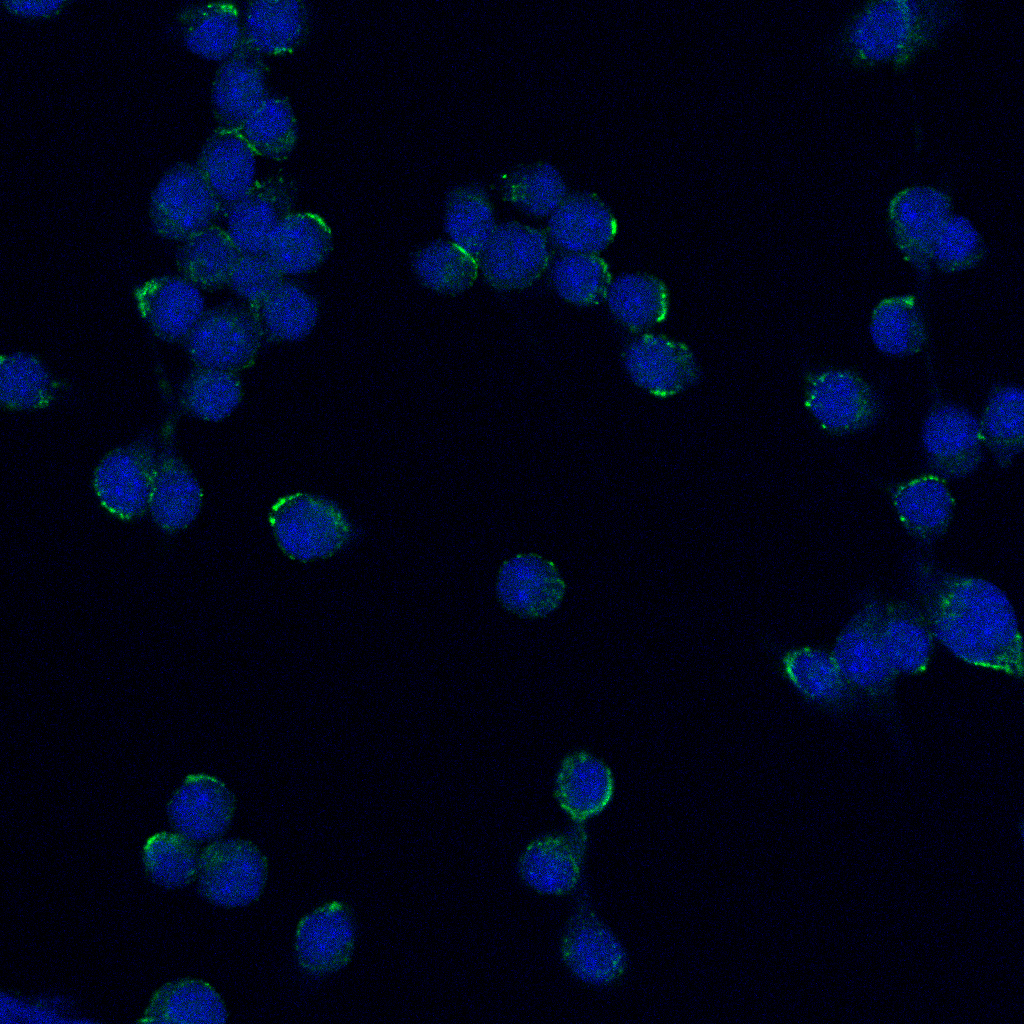

Supplement: Supplementary file 1 [file diseases-13-00060-s001.zip › source data-IF/all IF raw data/OELPS/OE LPS 1 600-5.tif]

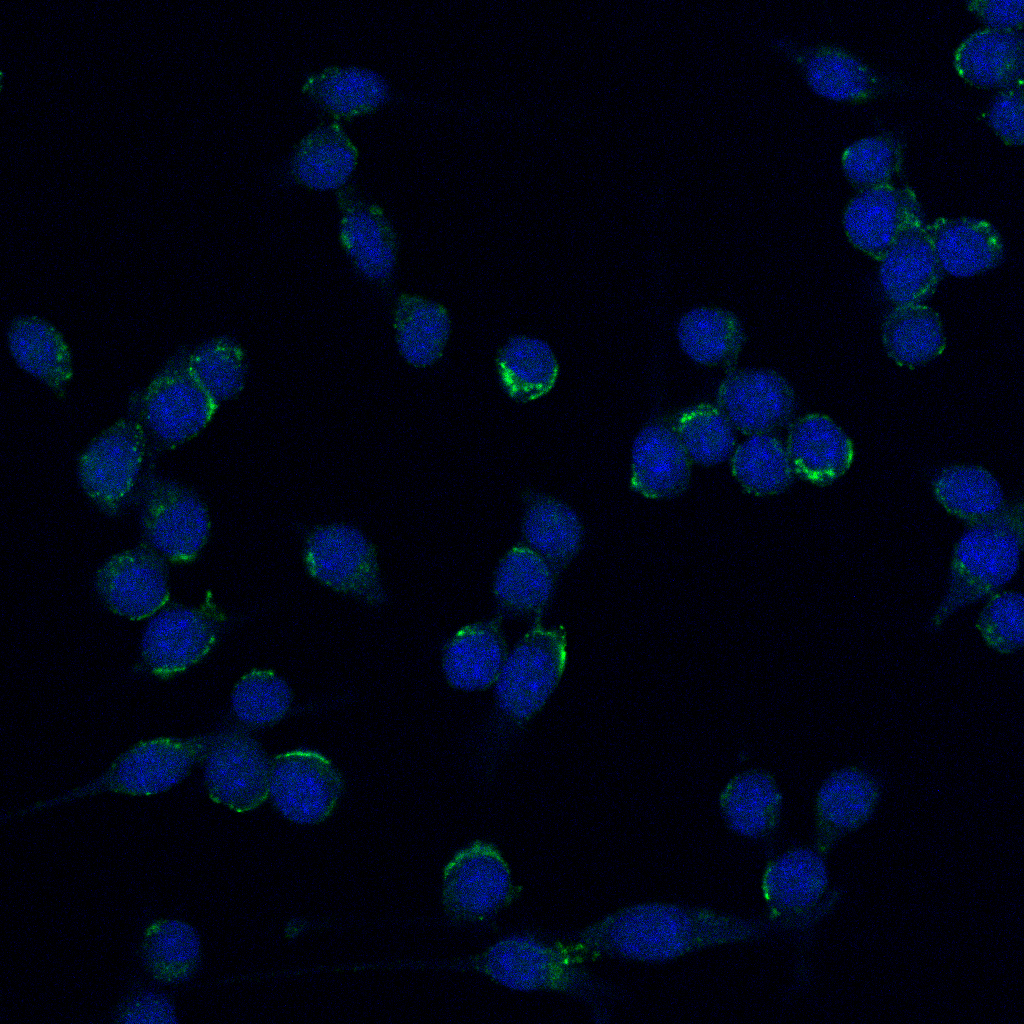

Supplement: Supplementary file 1 [file diseases-13-00060-s001.zip › source data-IF/all IF raw data/OELPS/OE LPS 1 600-6.tif]

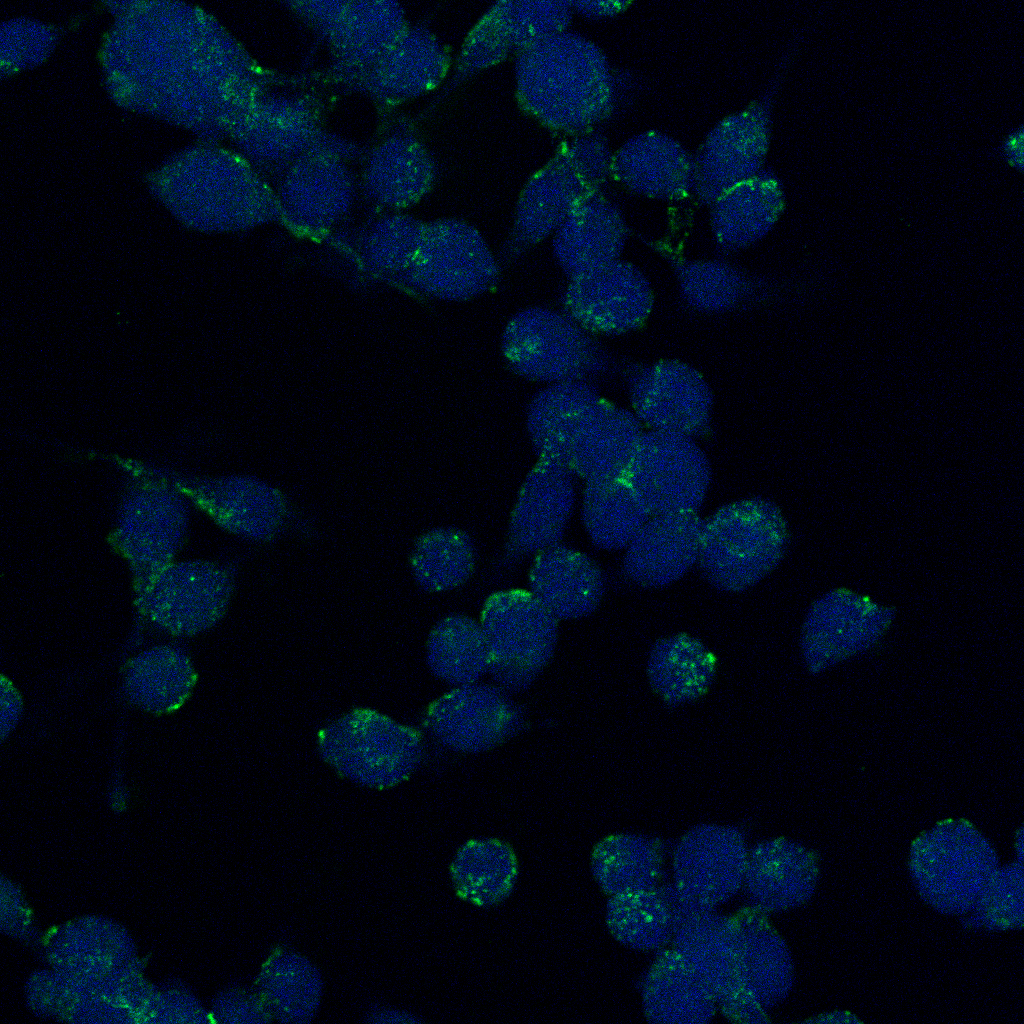

Supplement: Supplementary file 1 [file diseases-13-00060-s001.zip › source data-IF/all IF raw data/OELPS/OE LPS 2 600-1.tif]

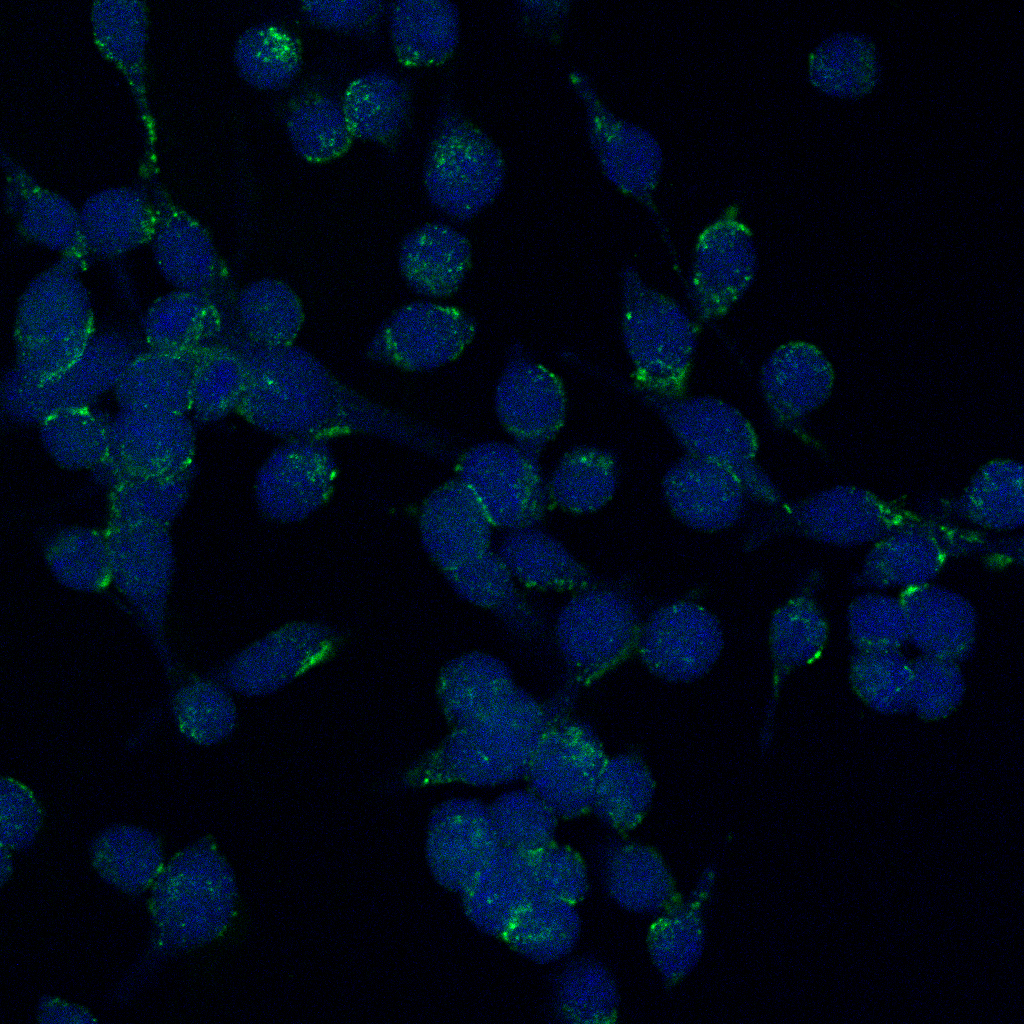

Supplement: Supplementary file 1 [file diseases-13-00060-s001.zip › source data-IF/all IF raw data/OELPS/OE LPS 2 600-2.tif]

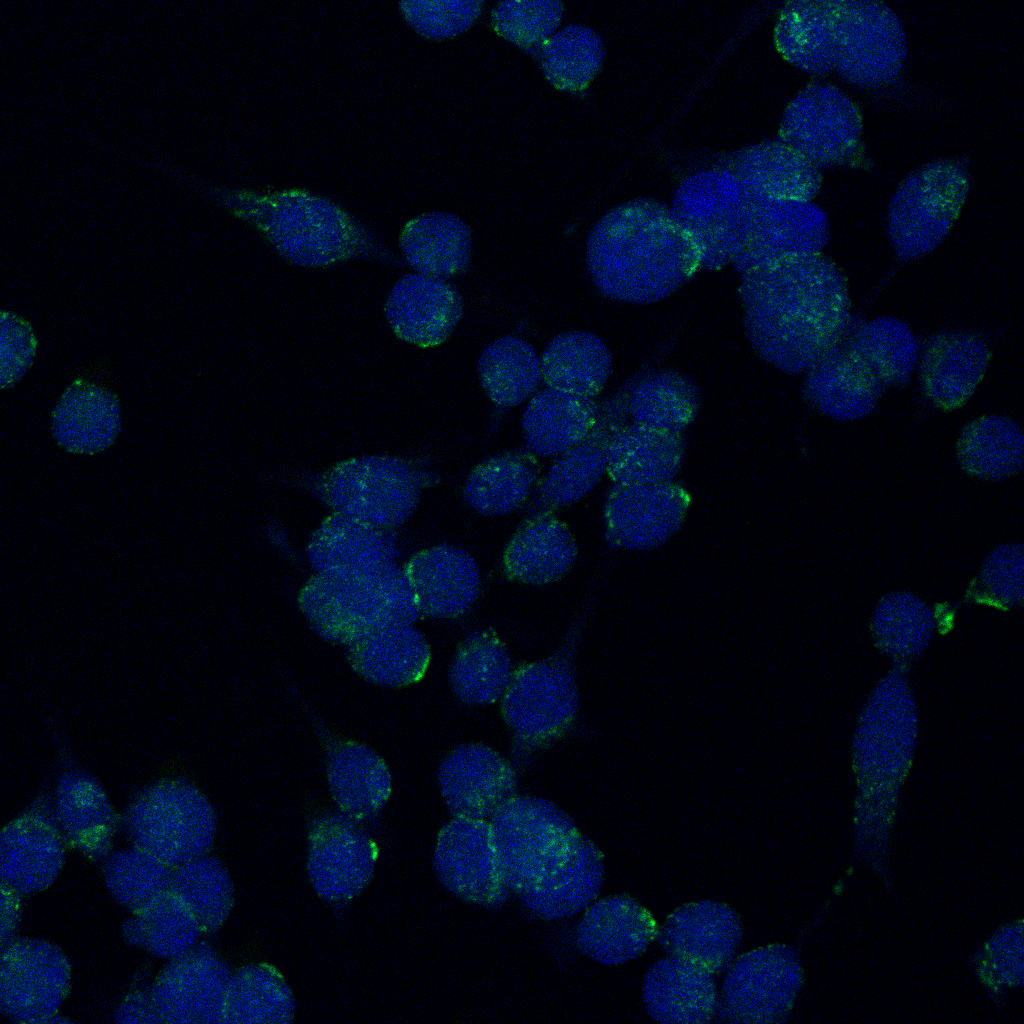

Supplement: Supplementary file 1 [file diseases-13-00060-s001.zip › source data-IF/all IF raw data/OELPS/OE LPS 2 600-3.tif]

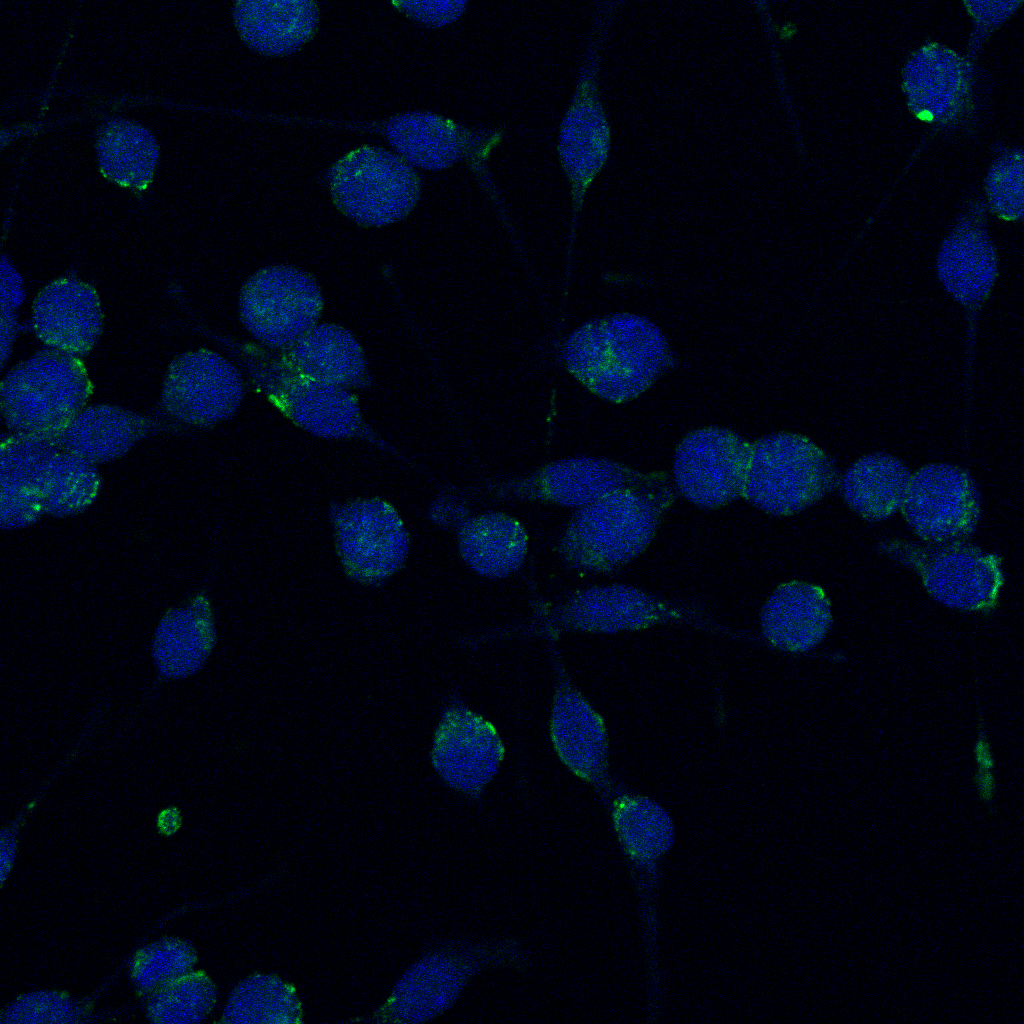

Supplement: Supplementary file 1 [file diseases-13-00060-s001.zip › source data-IF/all IF raw data/OELPS/OE LPS 2 600-4.tif]

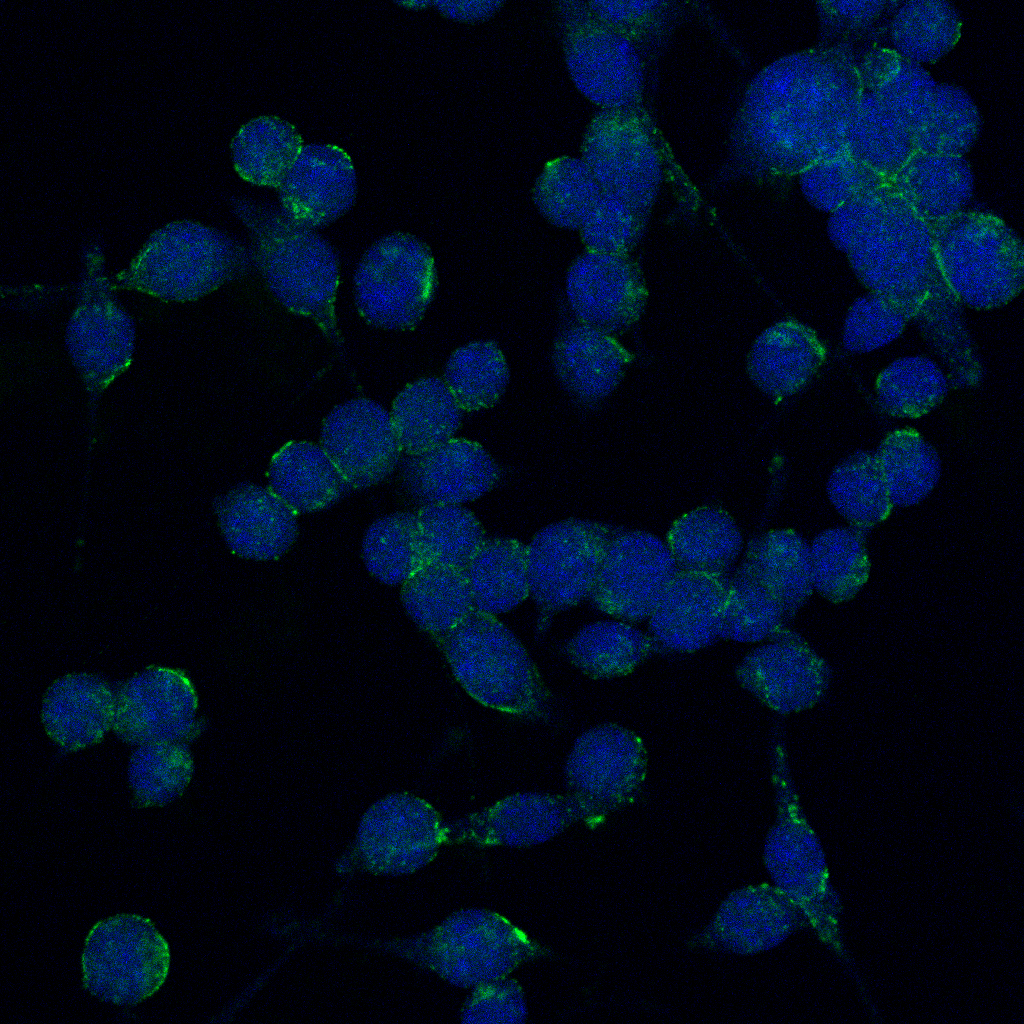

Supplement: Supplementary file 1 [file diseases-13-00060-s001.zip › source data-IF/all IF raw data/OELPS/OE LPS 2 600-5.tif]

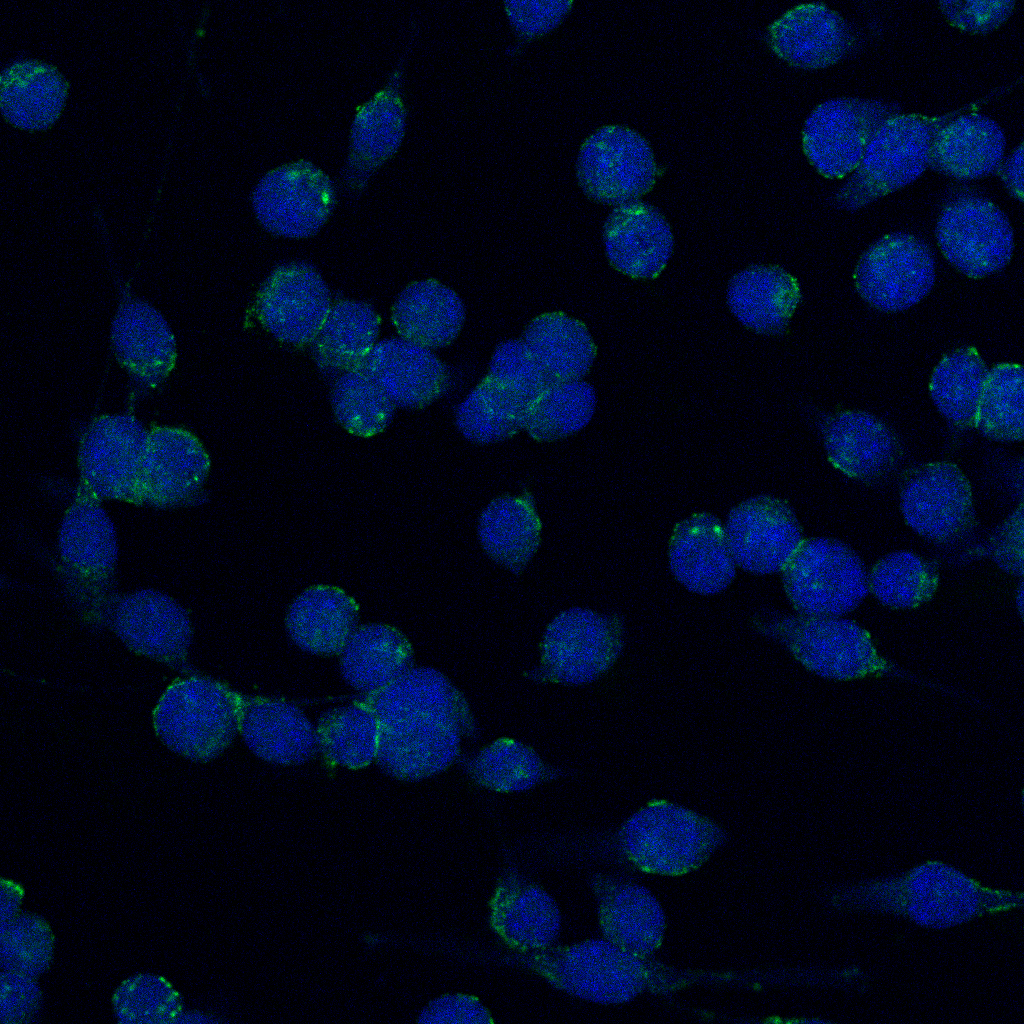

Supplement: Supplementary file 1 [file diseases-13-00060-s001.zip › source data-IF/all IF raw data/OELPS/OE LPS 2 600-6.tif]

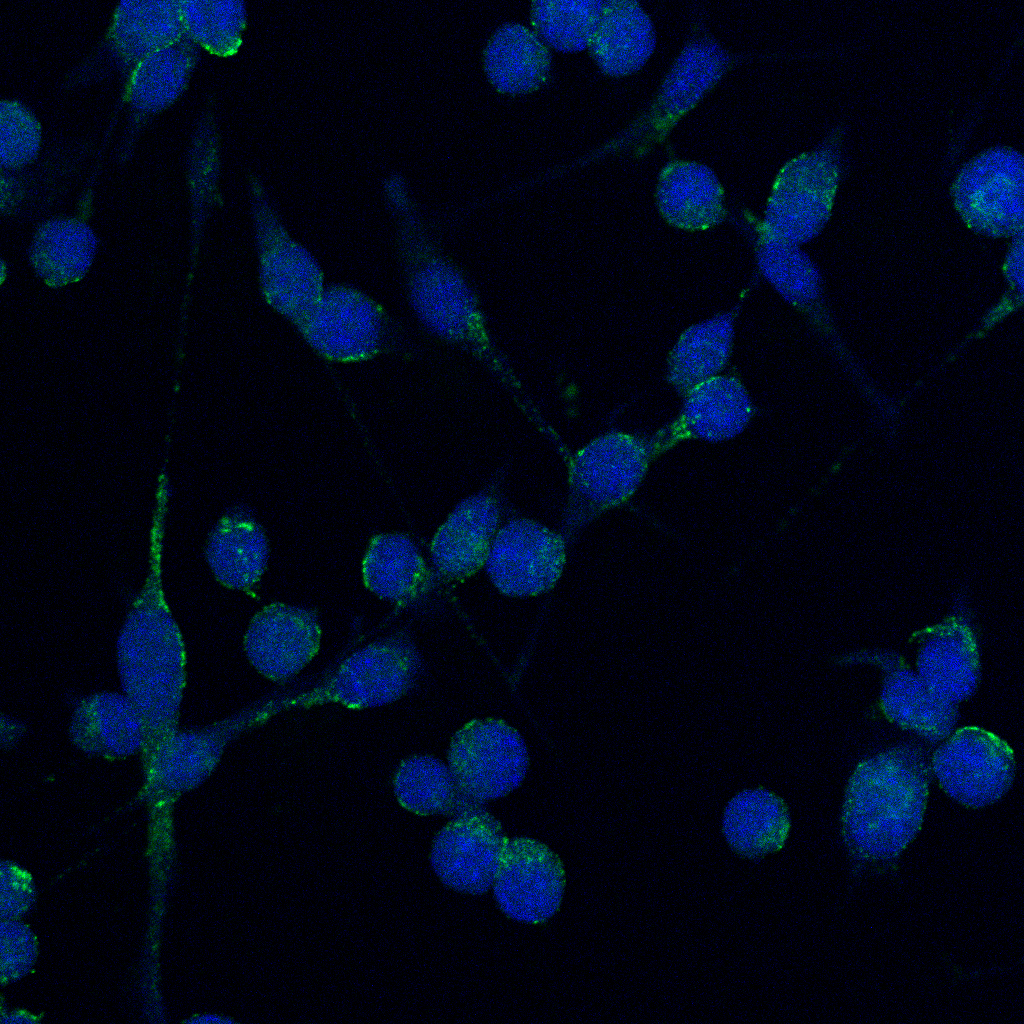

Supplement: Supplementary file 1 [file diseases-13-00060-s001.zip › source data-IF/all IF raw data/OELPS/OE LPS 3 600-1.tif]

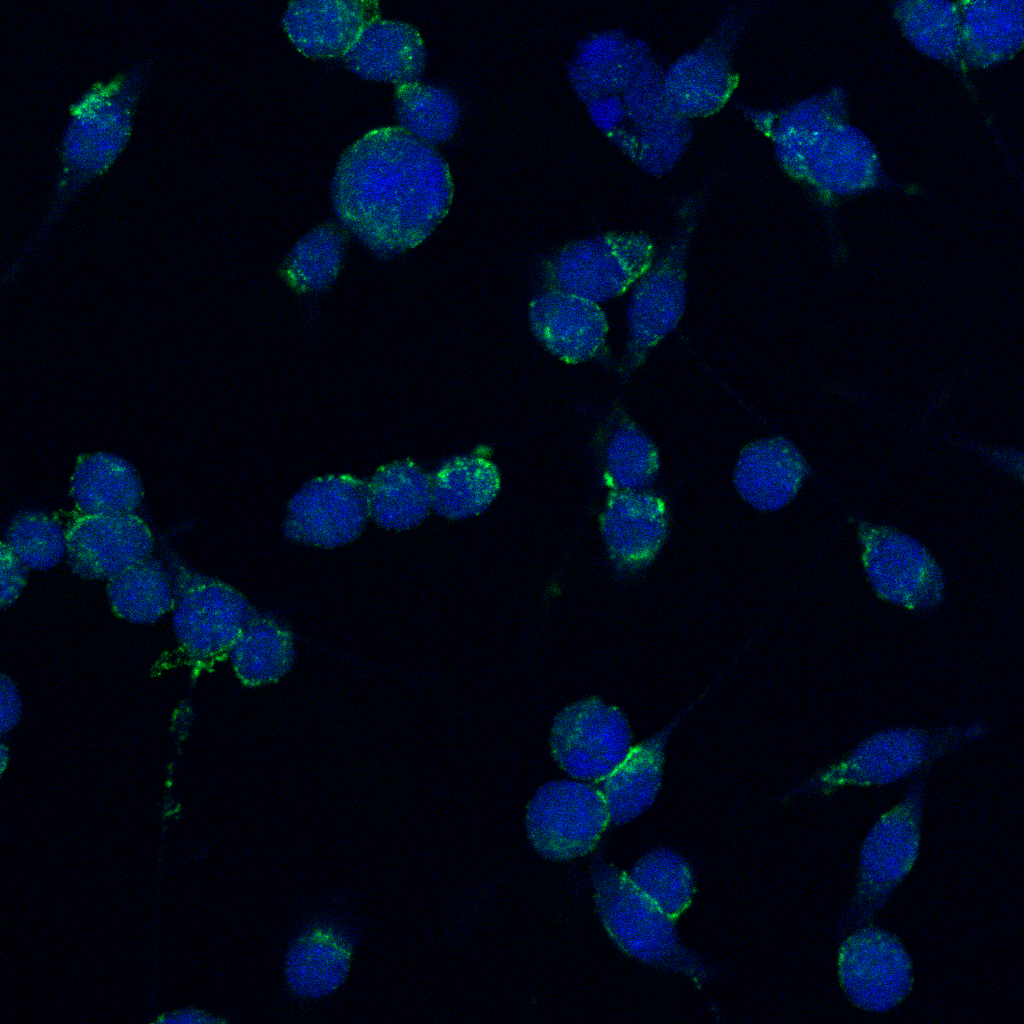

Supplement: Supplementary file 1 [file diseases-13-00060-s001.zip › source data-IF/all IF raw data/OELPS/OE LPS 3 600-2.tif]

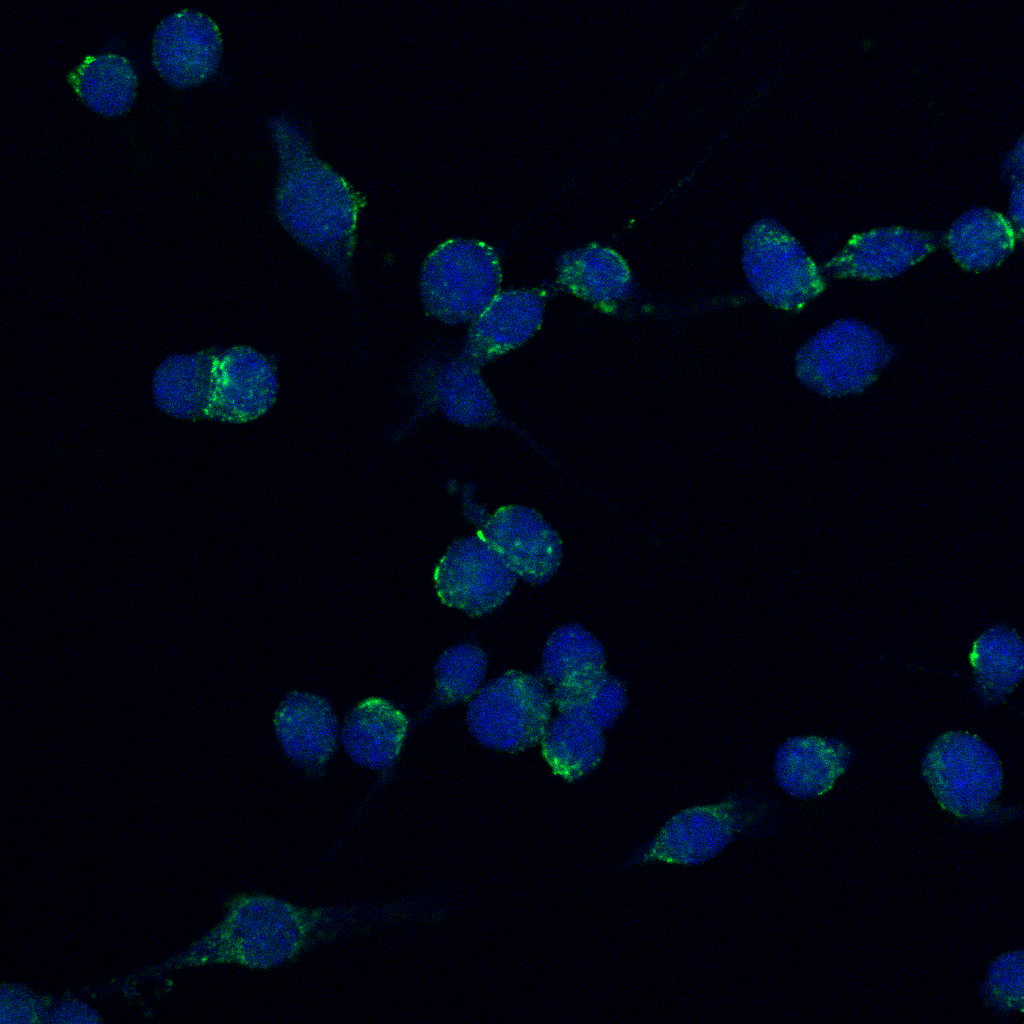

Supplement: Supplementary file 1 [file diseases-13-00060-s001.zip › source data-IF/all IF raw data/OELPS/OE LPS 3 600-3.tif]

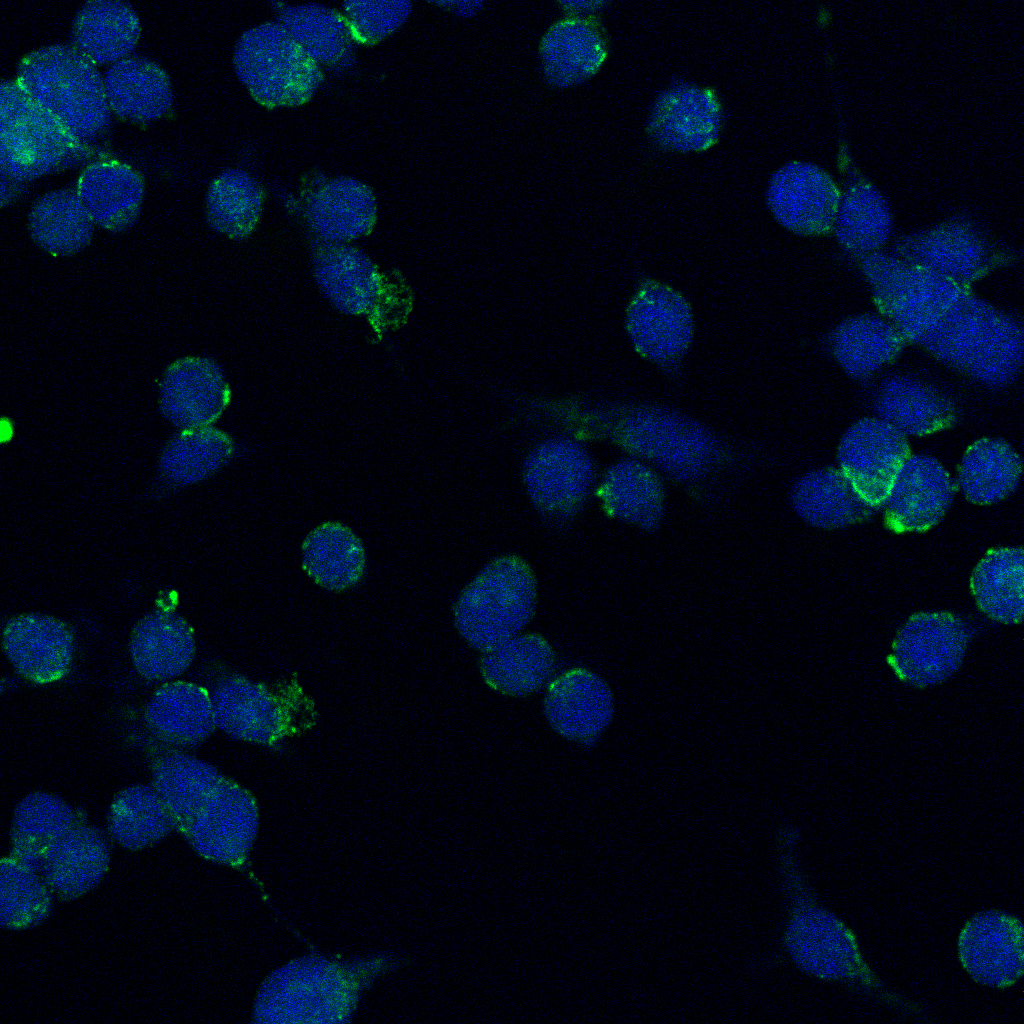

Supplement: Supplementary file 1 [file diseases-13-00060-s001.zip › source data-IF/all IF raw data/OELPS/OE LPS 3 600-4.tif]

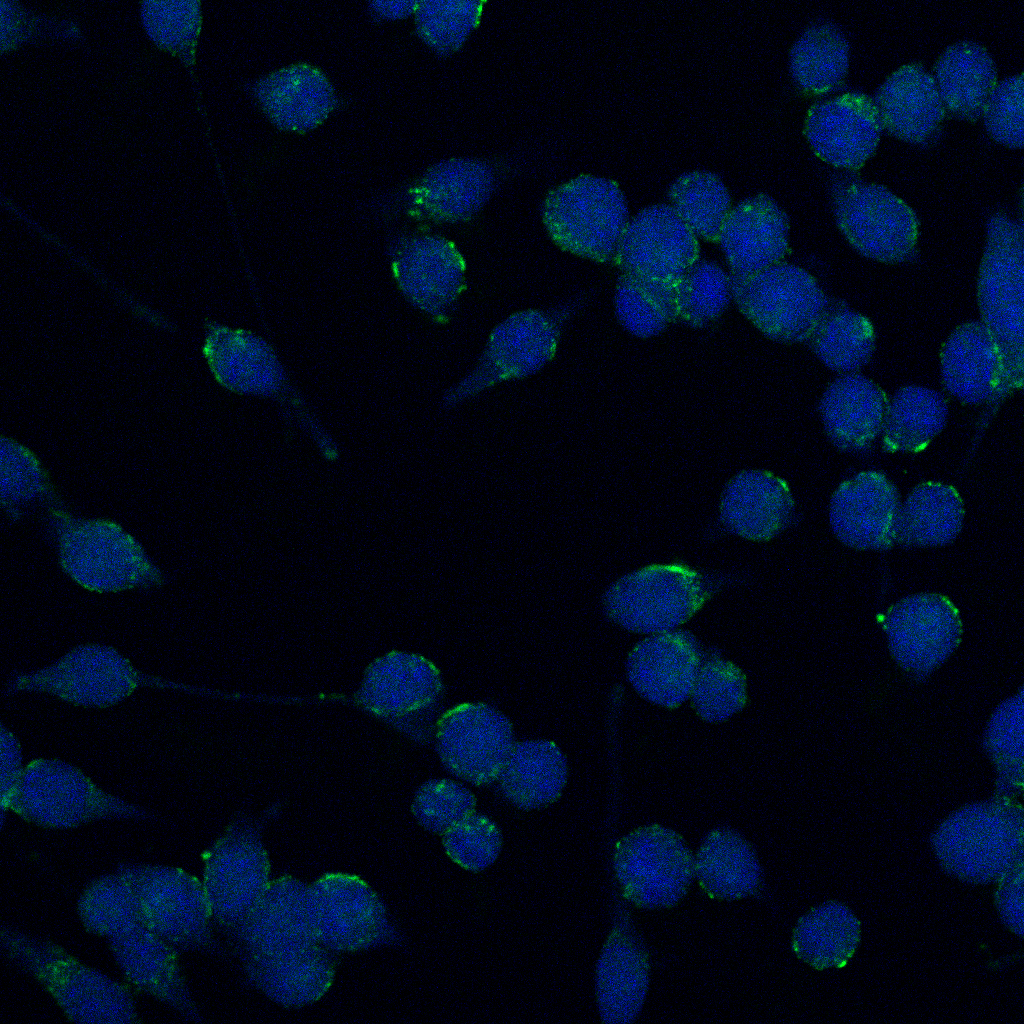

Supplement: Supplementary file 1 [file diseases-13-00060-s001.zip › source data-IF/all IF raw data/OELPS/OE LPS 3 600-5.tif]

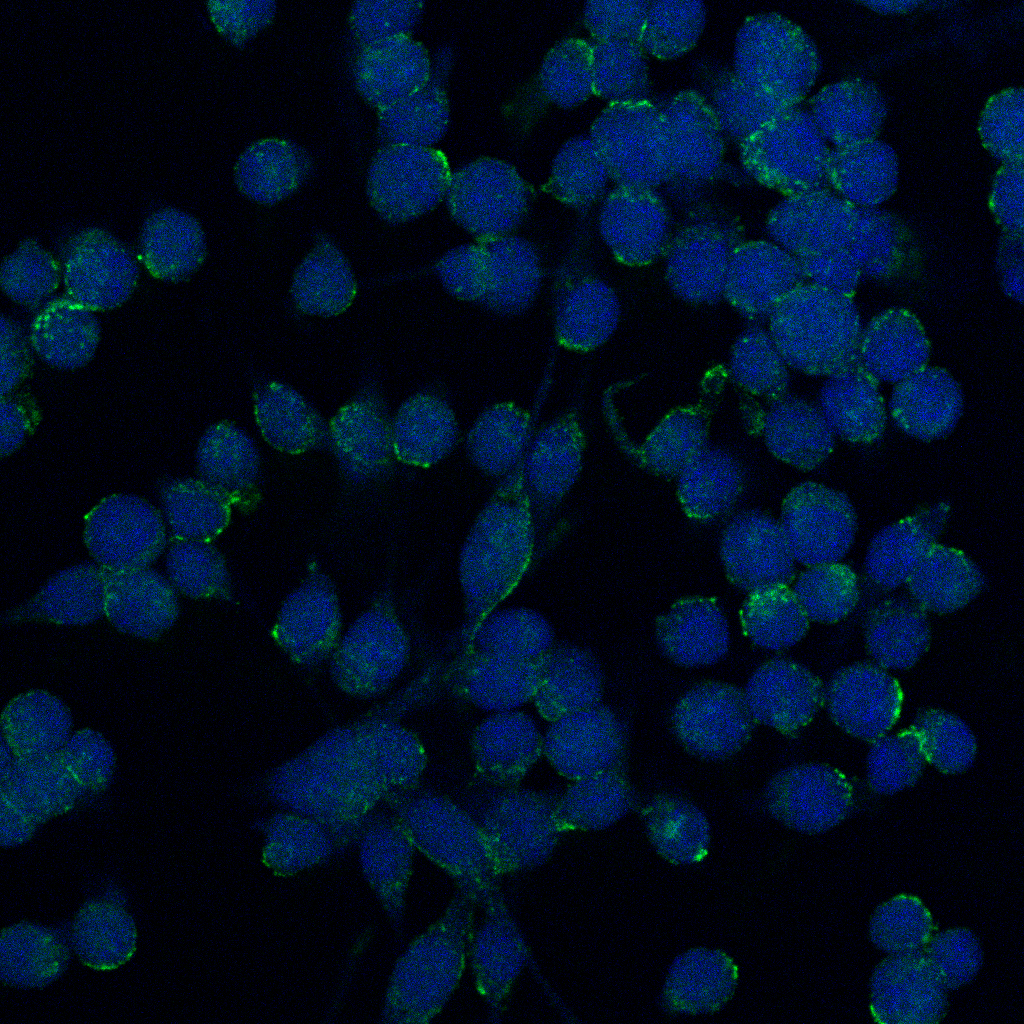

Supplement: Supplementary file 1 [file diseases-13-00060-s001.zip › source data-IF/all IF raw data/OELPS/OE LPS 3 600-6.tif]

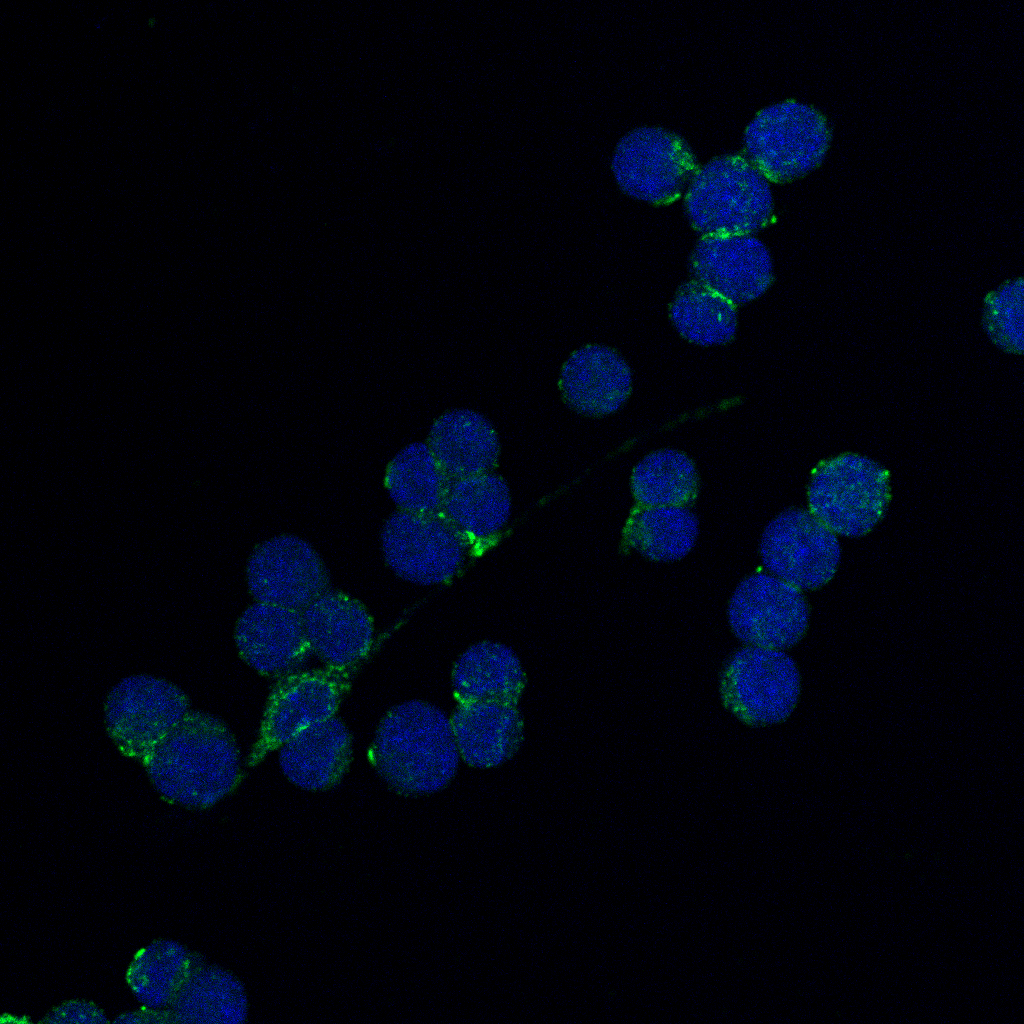

Supplement: Supplementary file 1 [file diseases-13-00060-s001.zip › source data-IF/all IF raw data/WT/WT-1 600-1.tif]

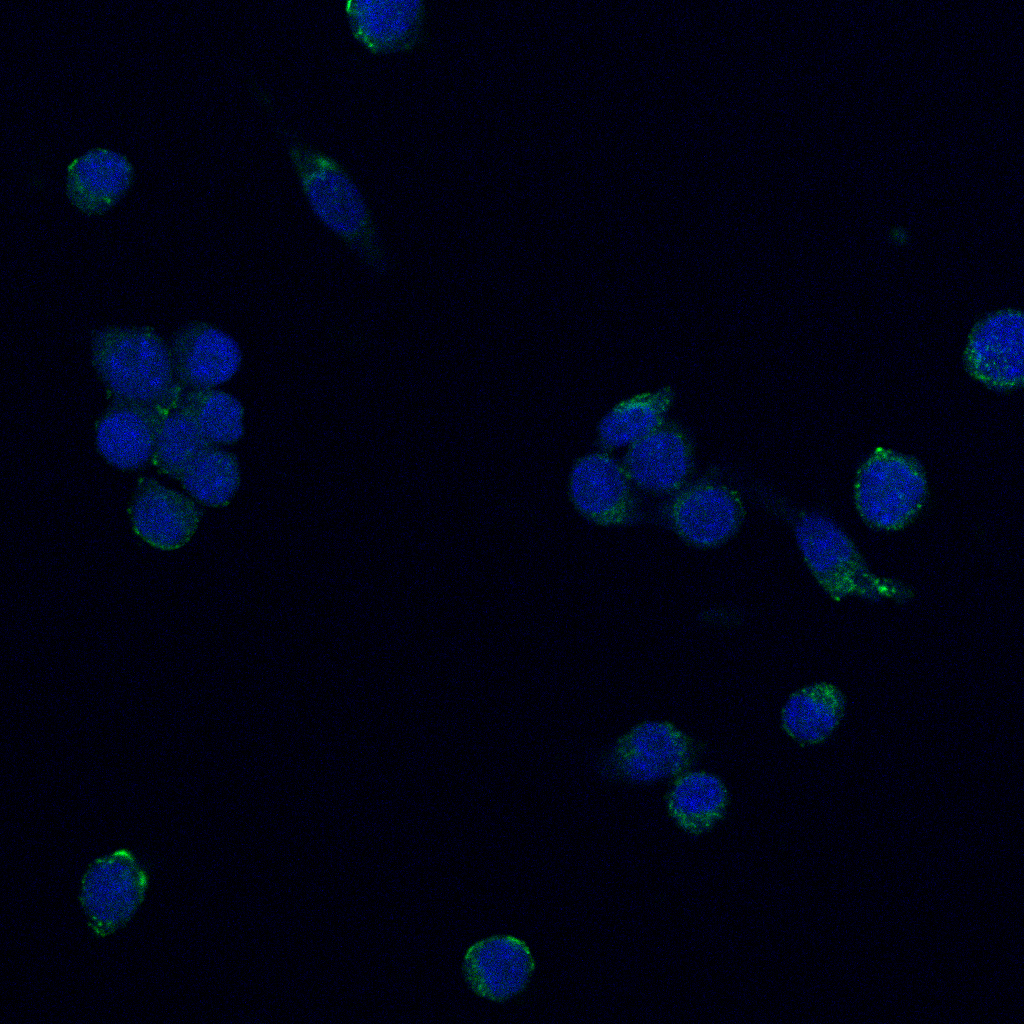

Supplement: Supplementary file 1 [file diseases-13-00060-s001.zip › source data-IF/all IF raw data/WT/WT-1 600-2.tif]

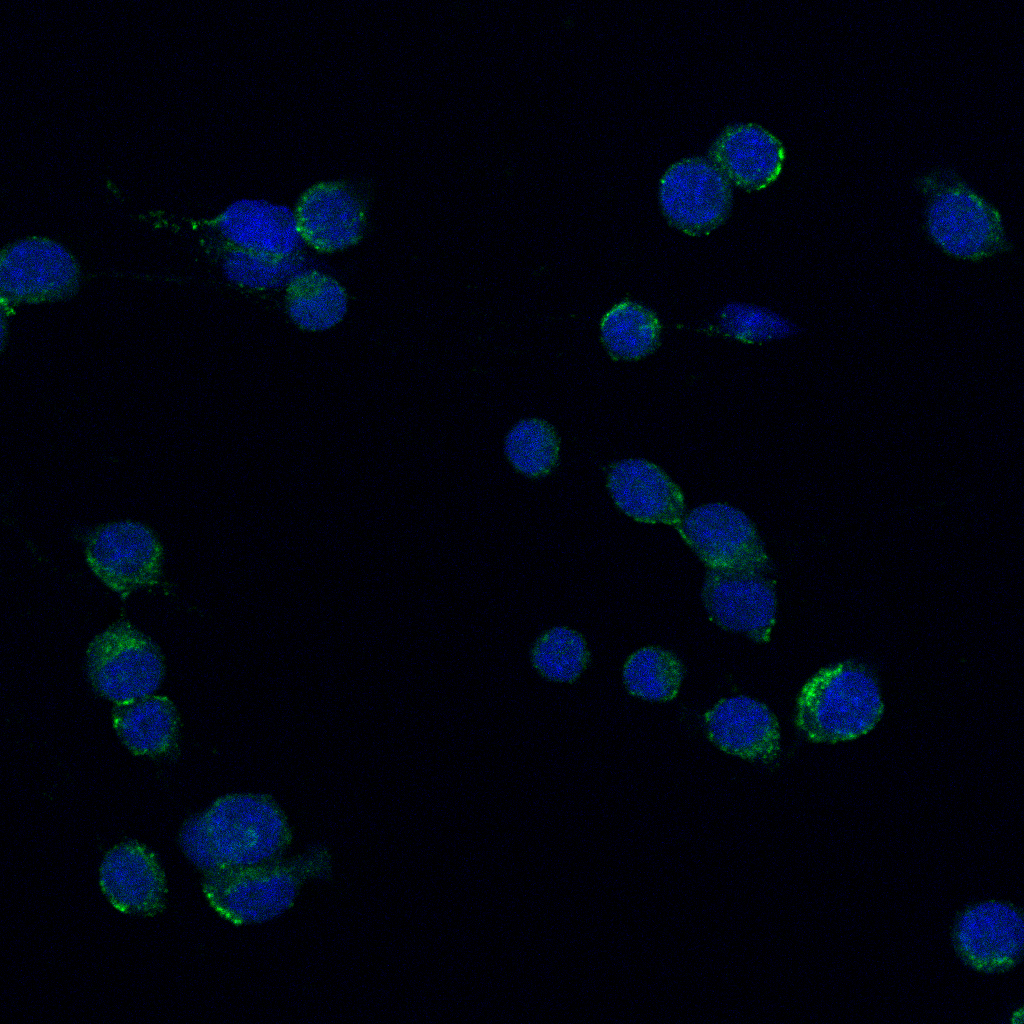

Supplement: Supplementary file 1 [file diseases-13-00060-s001.zip › source data-IF/all IF raw data/WT/WT-1 600-3.tif]

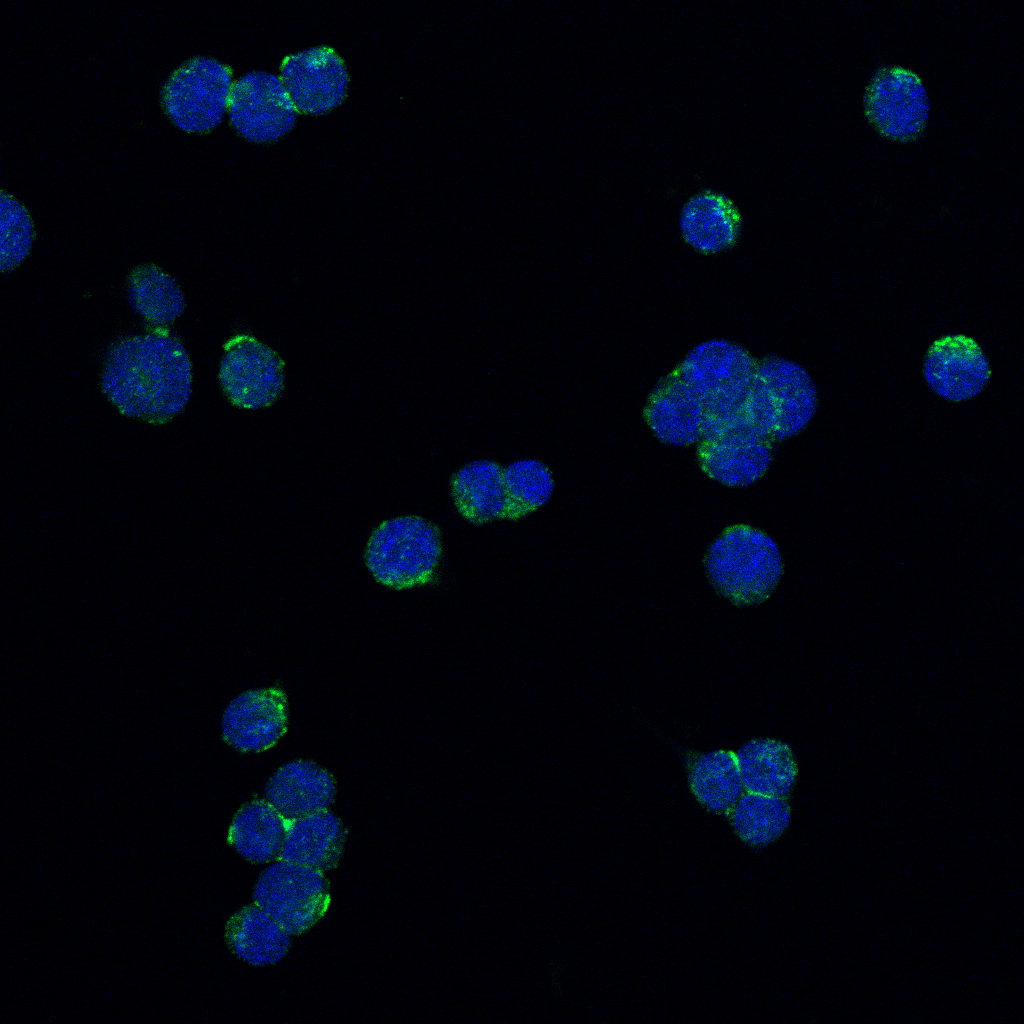

Supplement: Supplementary file 1 [file diseases-13-00060-s001.zip › source data-IF/all IF raw data/WT/WT-1 600-4.tif]

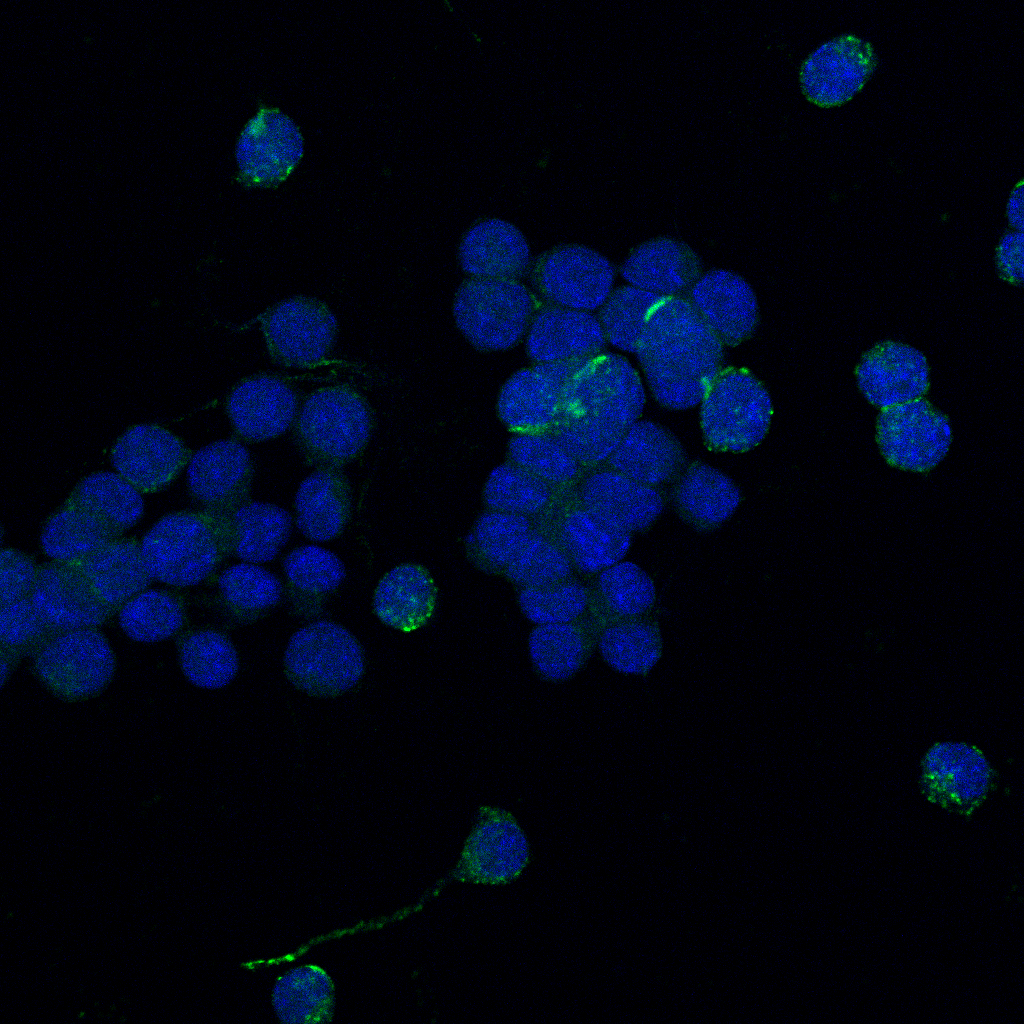

Supplement: Supplementary file 1 [file diseases-13-00060-s001.zip › source data-IF/all IF raw data/WT/WT-1 600-5.tif]

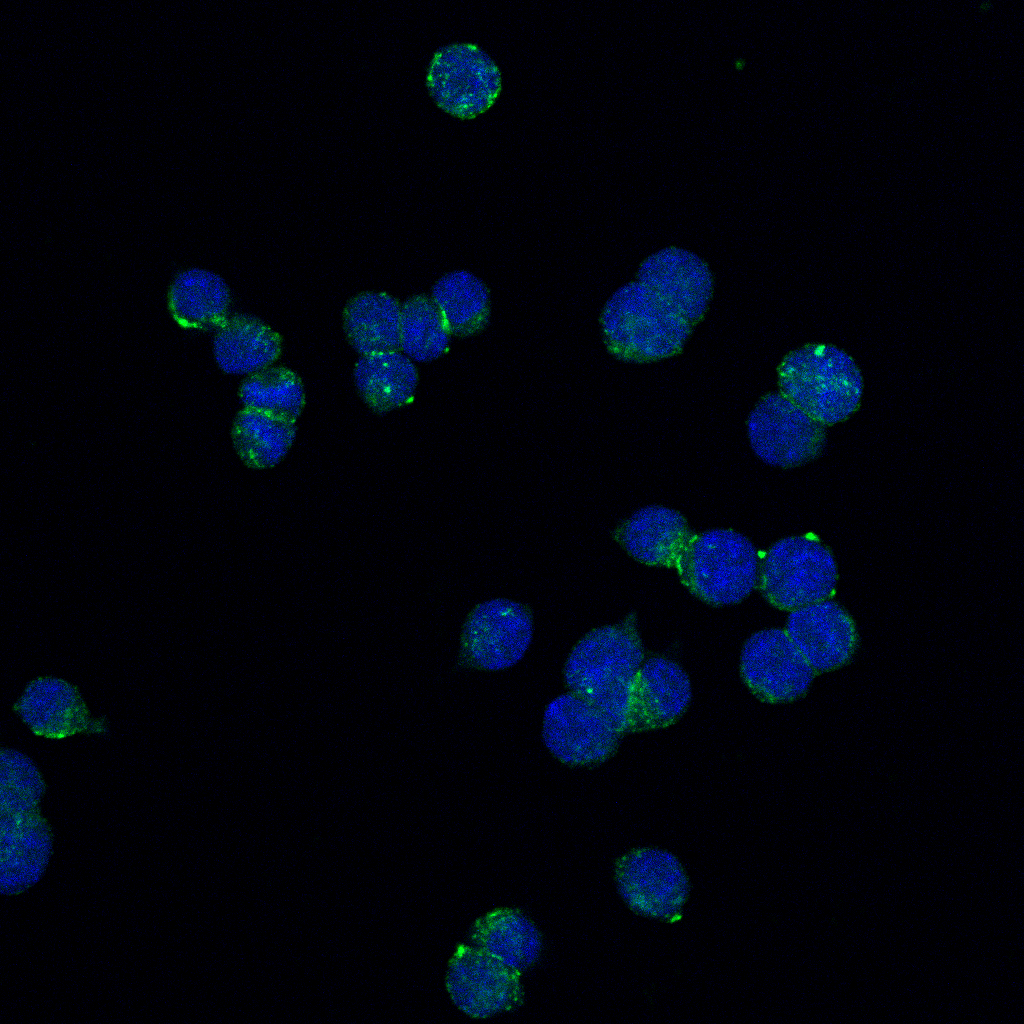

Supplement: Supplementary file 1 [file diseases-13-00060-s001.zip › source data-IF/all IF raw data/WT/WT-1 600-6.tif]

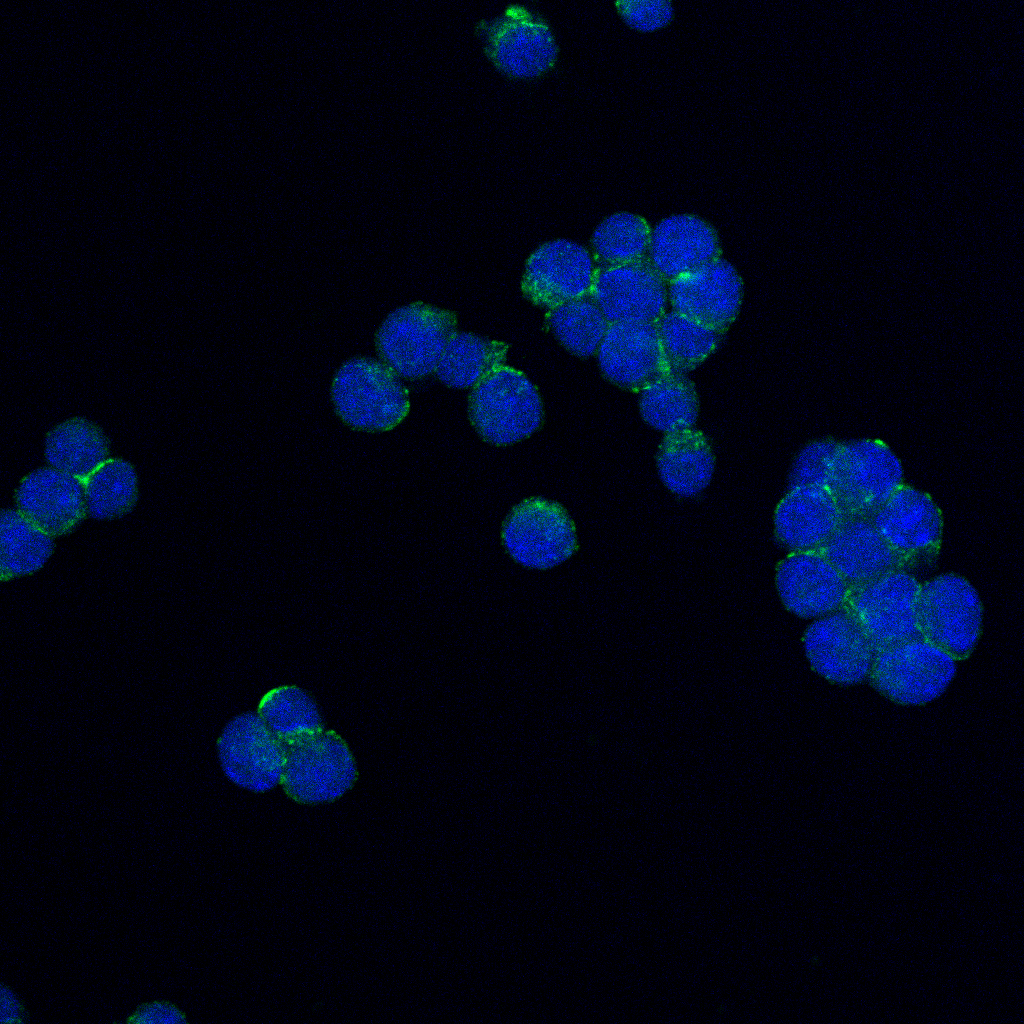

Supplement: Supplementary file 1 [file diseases-13-00060-s001.zip › source data-IF/all IF raw data/WT/WT-2 600-1.tif]

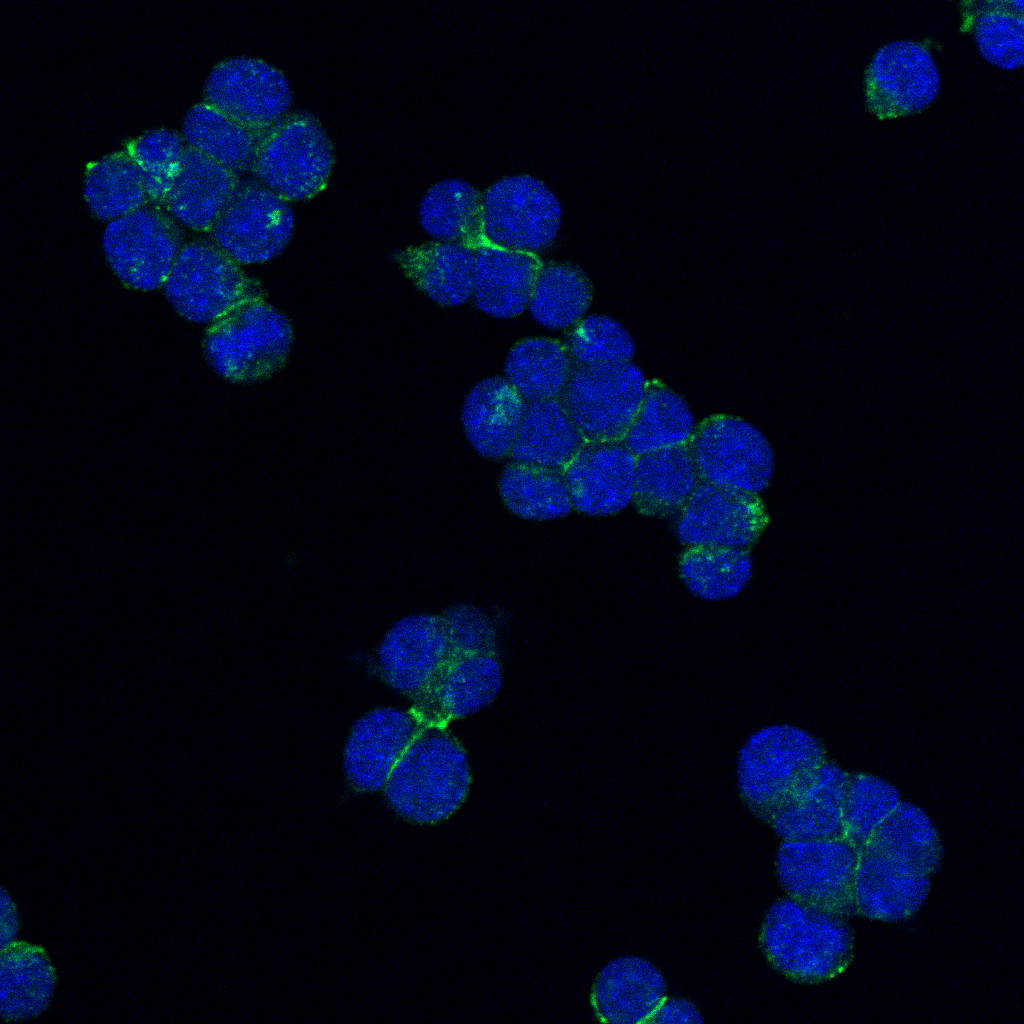

Supplement: Supplementary file 1 [file diseases-13-00060-s001.zip › source data-IF/all IF raw data/WT/WT-2 600-2.tif]

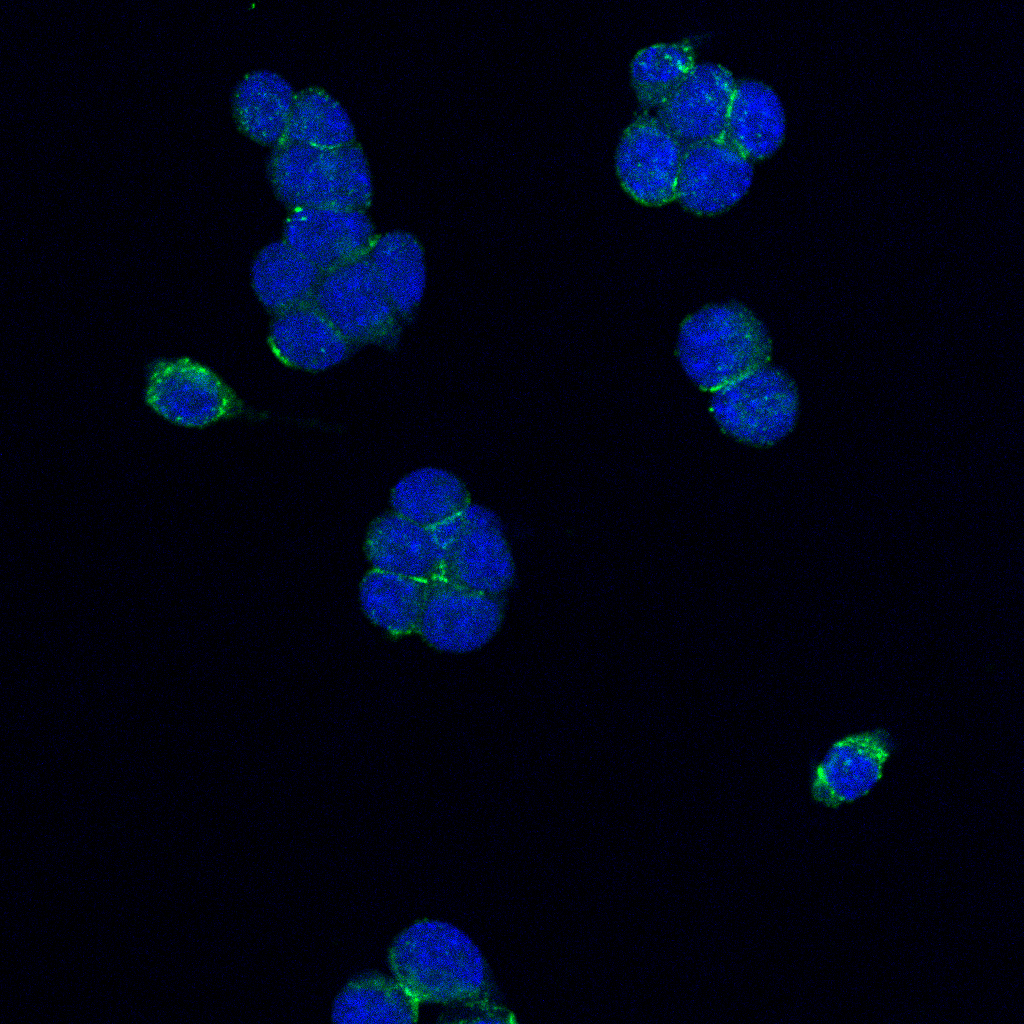

Supplement: Supplementary file 1 [file diseases-13-00060-s001.zip › source data-IF/all IF raw data/WT/WT-2 600-3.tif]

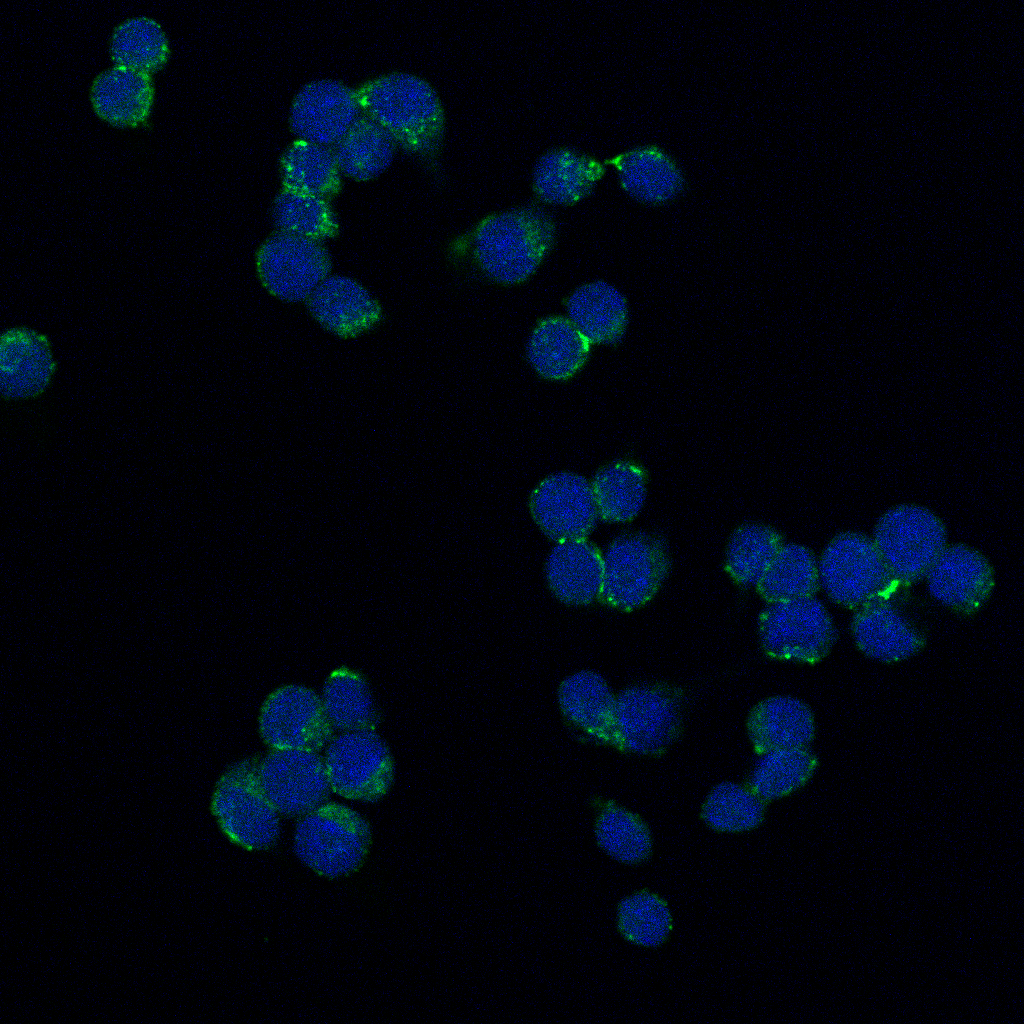

Supplement: Supplementary file 1 [file diseases-13-00060-s001.zip › source data-IF/all IF raw data/WT/WT-2 600-4.tif]

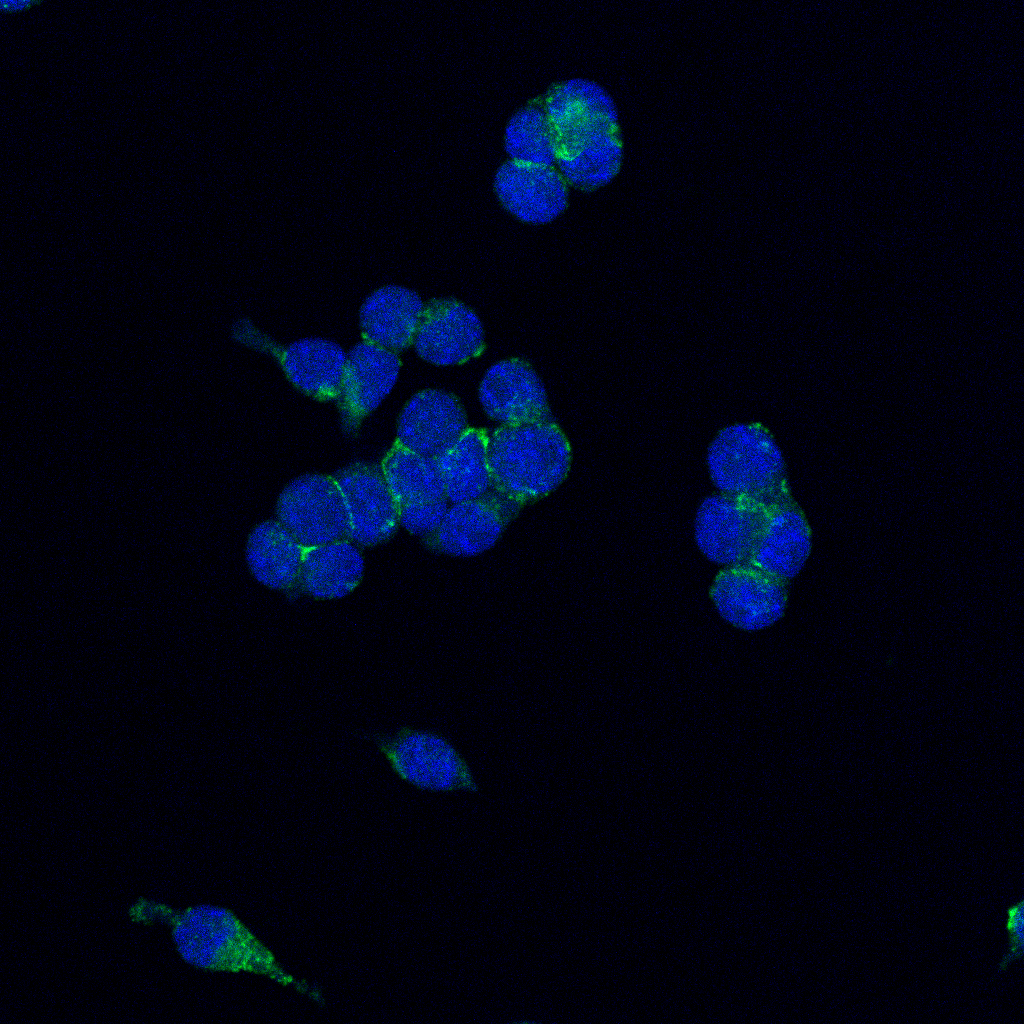

Supplement: Supplementary file 1 [file diseases-13-00060-s001.zip › source data-IF/all IF raw data/WT/WT-2 600-5.tif]

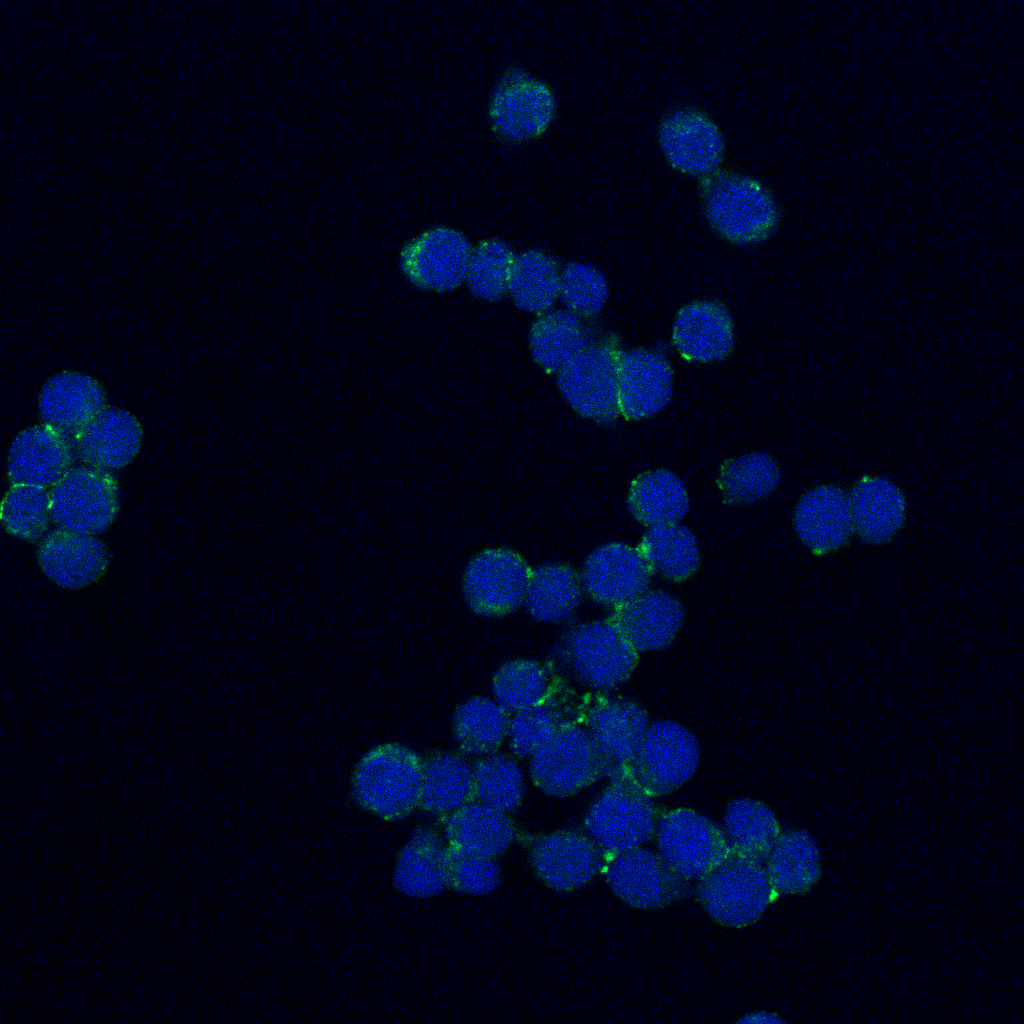

Supplement: Supplementary file 1 [file diseases-13-00060-s001.zip › source data-IF/all IF raw data/WT/WT-2 600-6.tif]

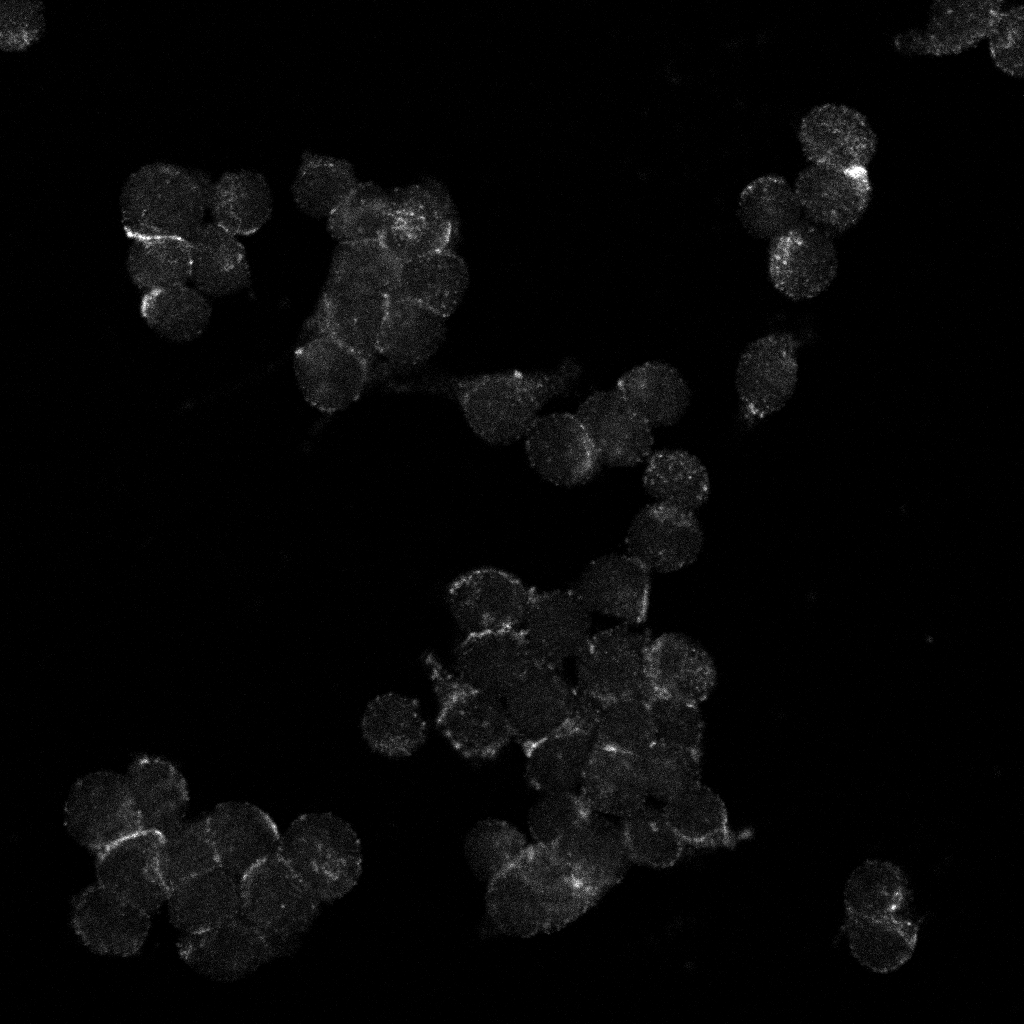

Supplement: Supplementary file 1 [file diseases-13-00060-s001.zip › source data-IF/all IF raw data/WT/WT-3 600-1.tif]

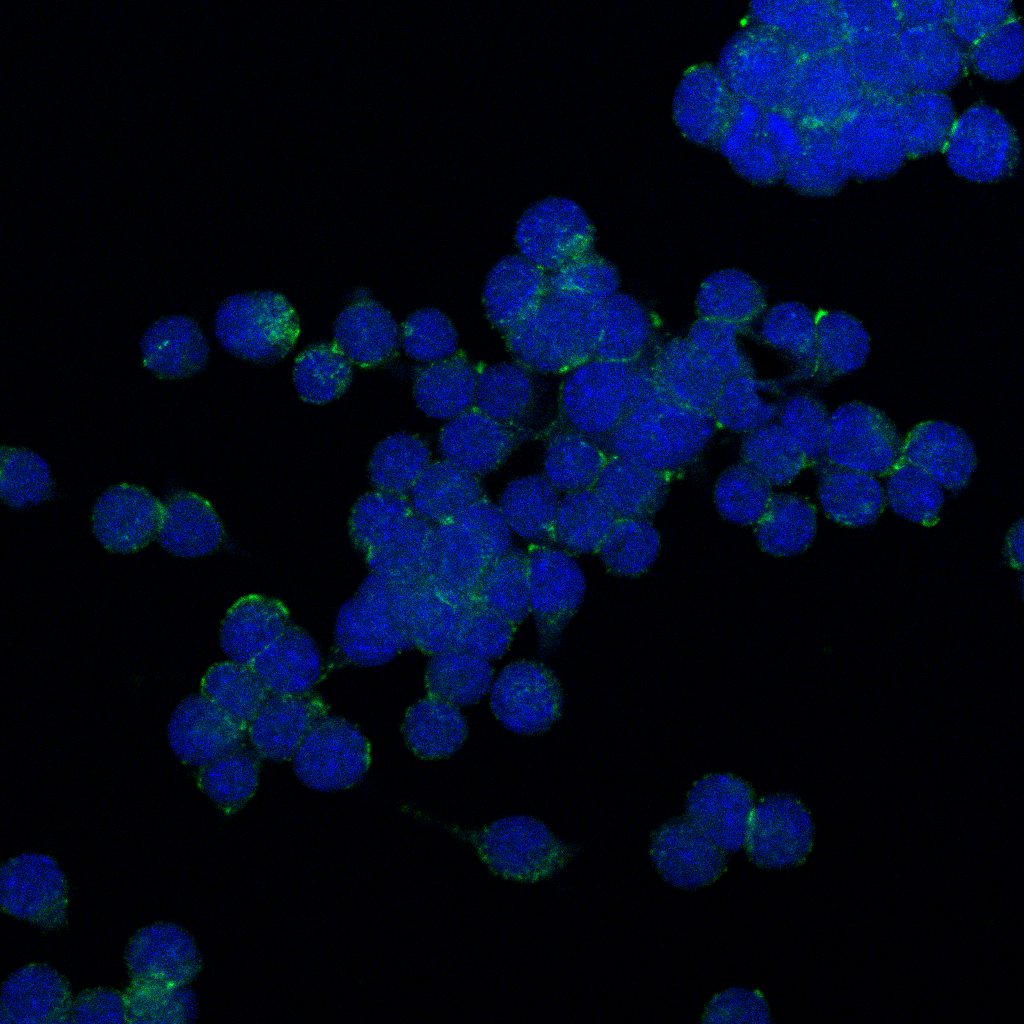

Supplement: Supplementary file 1 [file diseases-13-00060-s001.zip › source data-IF/all IF raw data/WT/WT-3 600-2.tif]

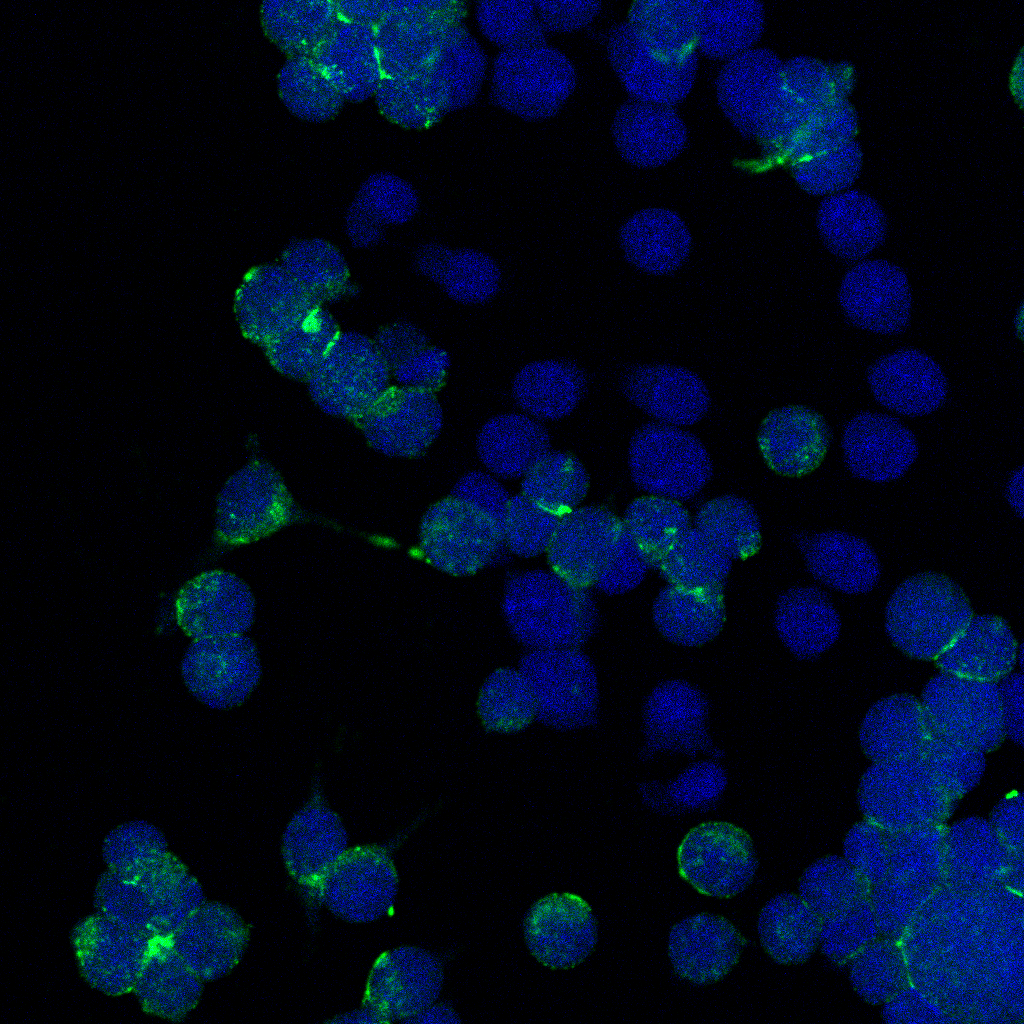

Supplement: Supplementary file 1 [file diseases-13-00060-s001.zip › source data-IF/all IF raw data/WT/WT-3 600-3.tif]

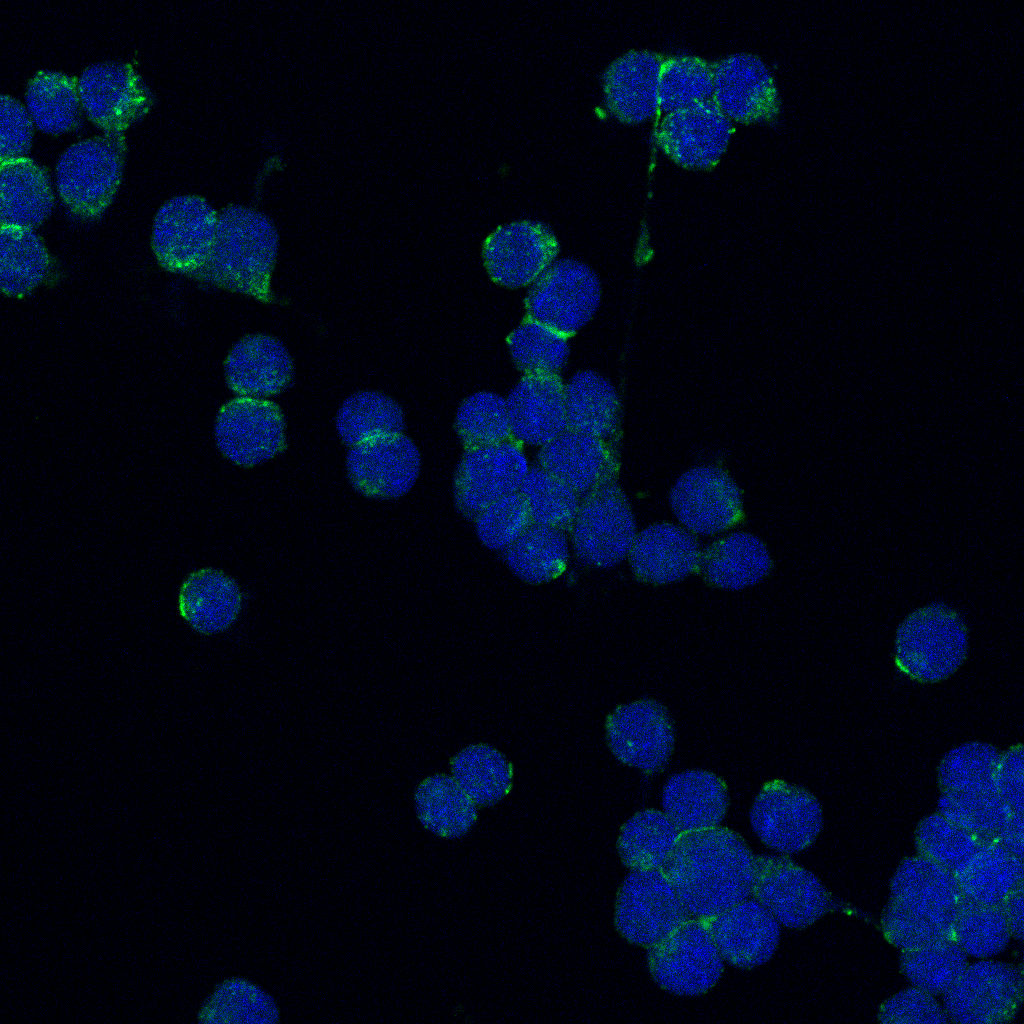

Supplement: Supplementary file 1 [file diseases-13-00060-s001.zip › source data-IF/all IF raw data/WT/WT-3 600-4.tif]

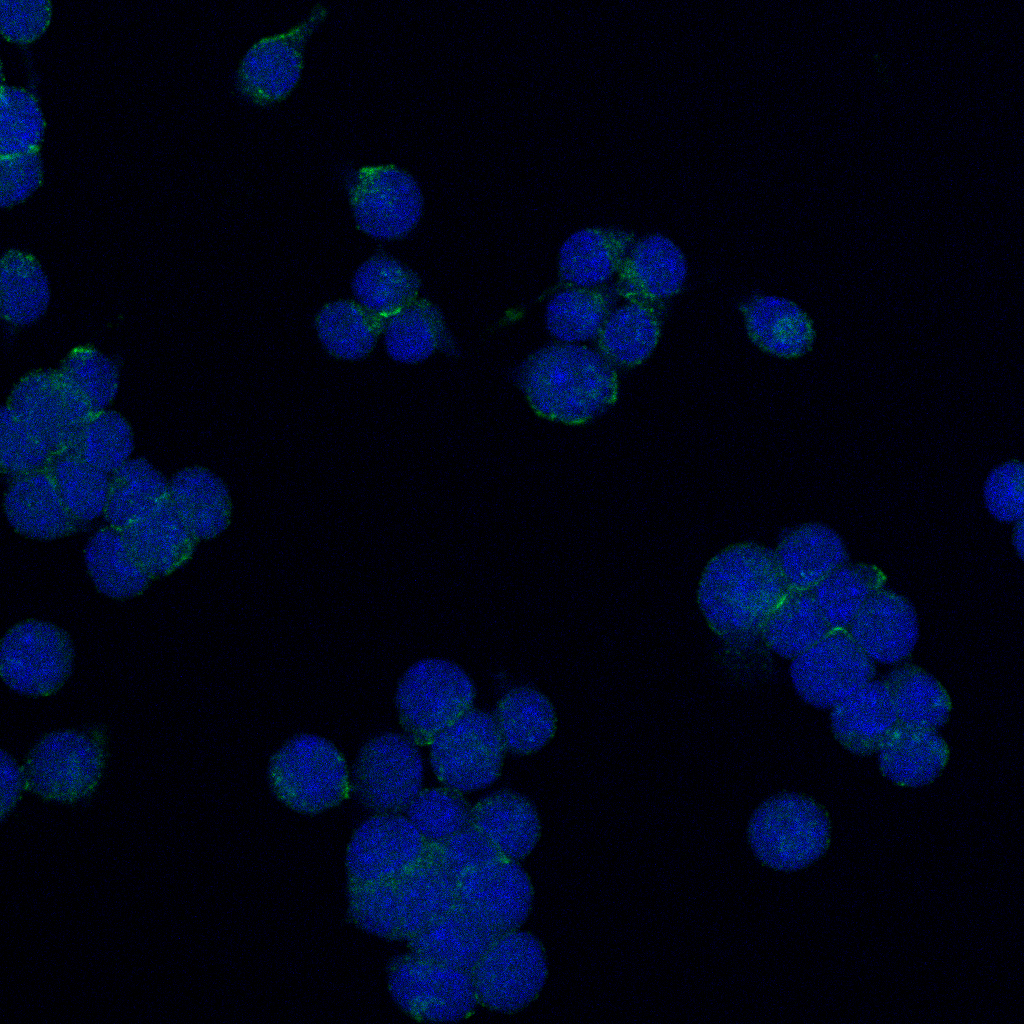

Supplement: Supplementary file 1 [file diseases-13-00060-s001.zip › source data-IF/all IF raw data/WT/WT-3 600-5.tif]

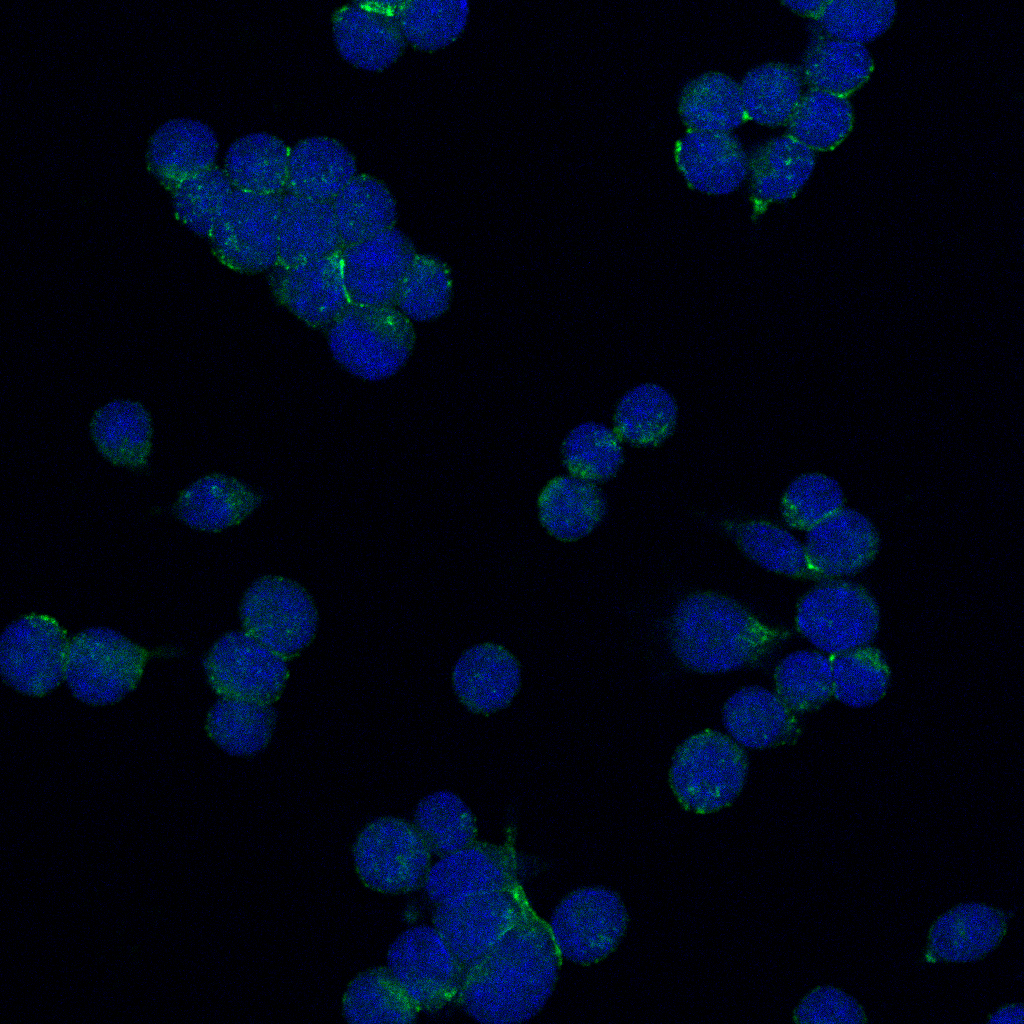

Supplement: Supplementary file 1 [file diseases-13-00060-s001.zip › source data-IF/all IF raw data/WT/WT-3 600-6.tif]

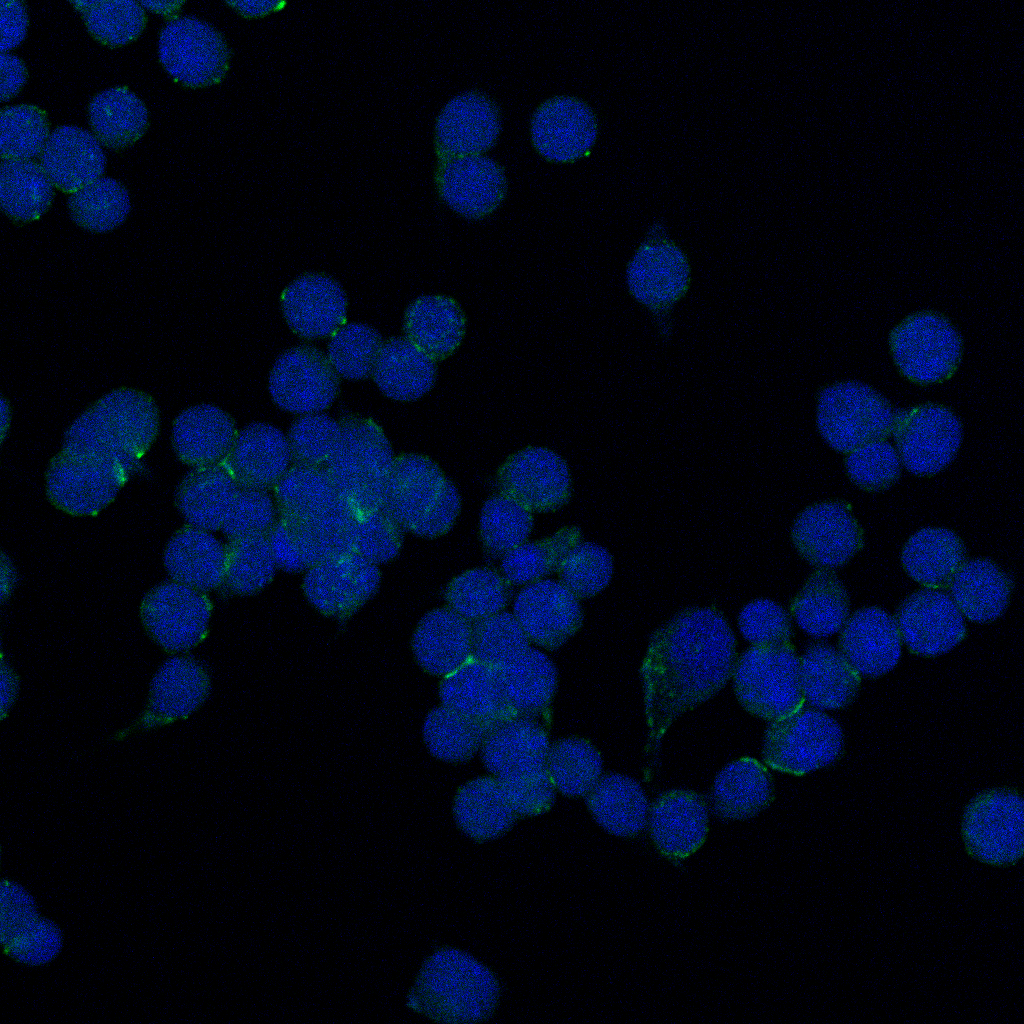

Supplement: Supplementary file 1 [file diseases-13-00060-s001.zip › source data-IF/all IF raw data/WTLPS/WT LPS 1 600-1.tif]

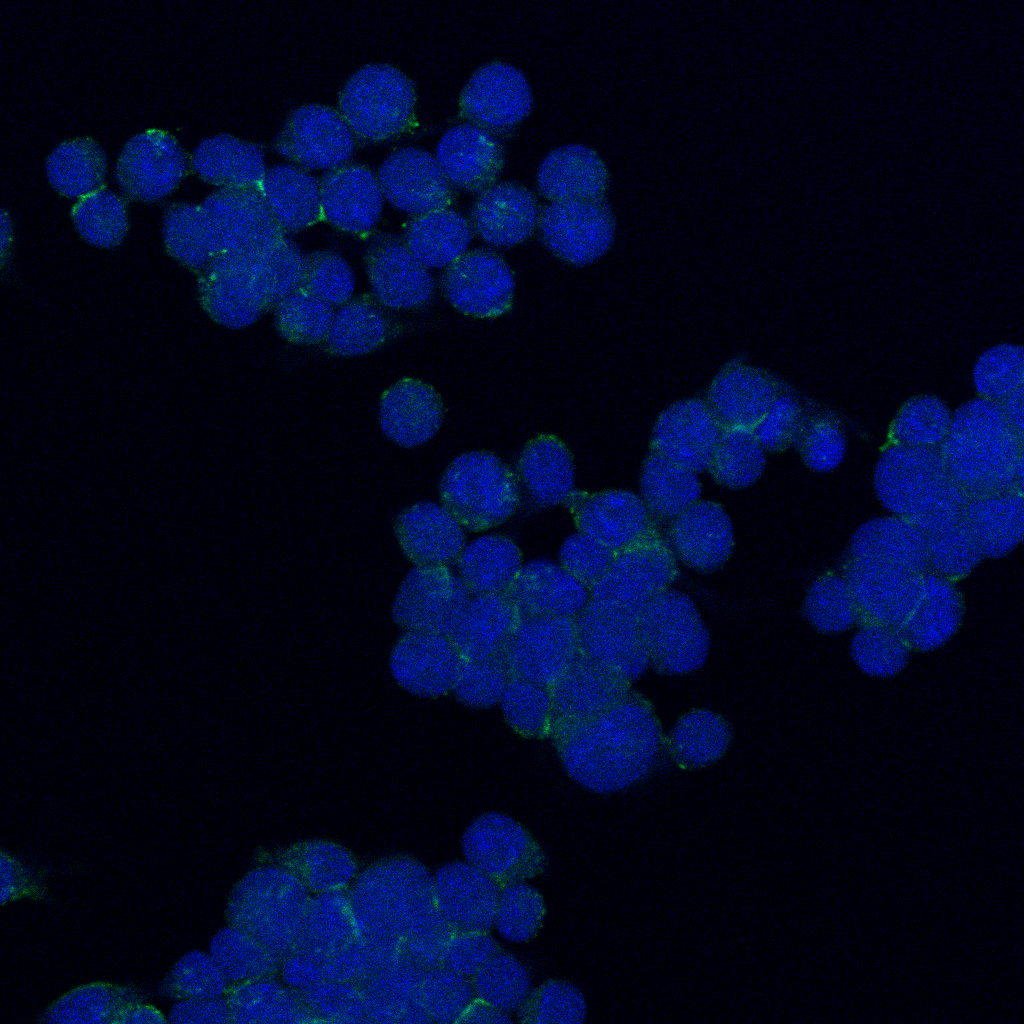

Supplement: Supplementary file 1 [file diseases-13-00060-s001.zip › source data-IF/all IF raw data/WTLPS/WT LPS 1 600-2.tif]

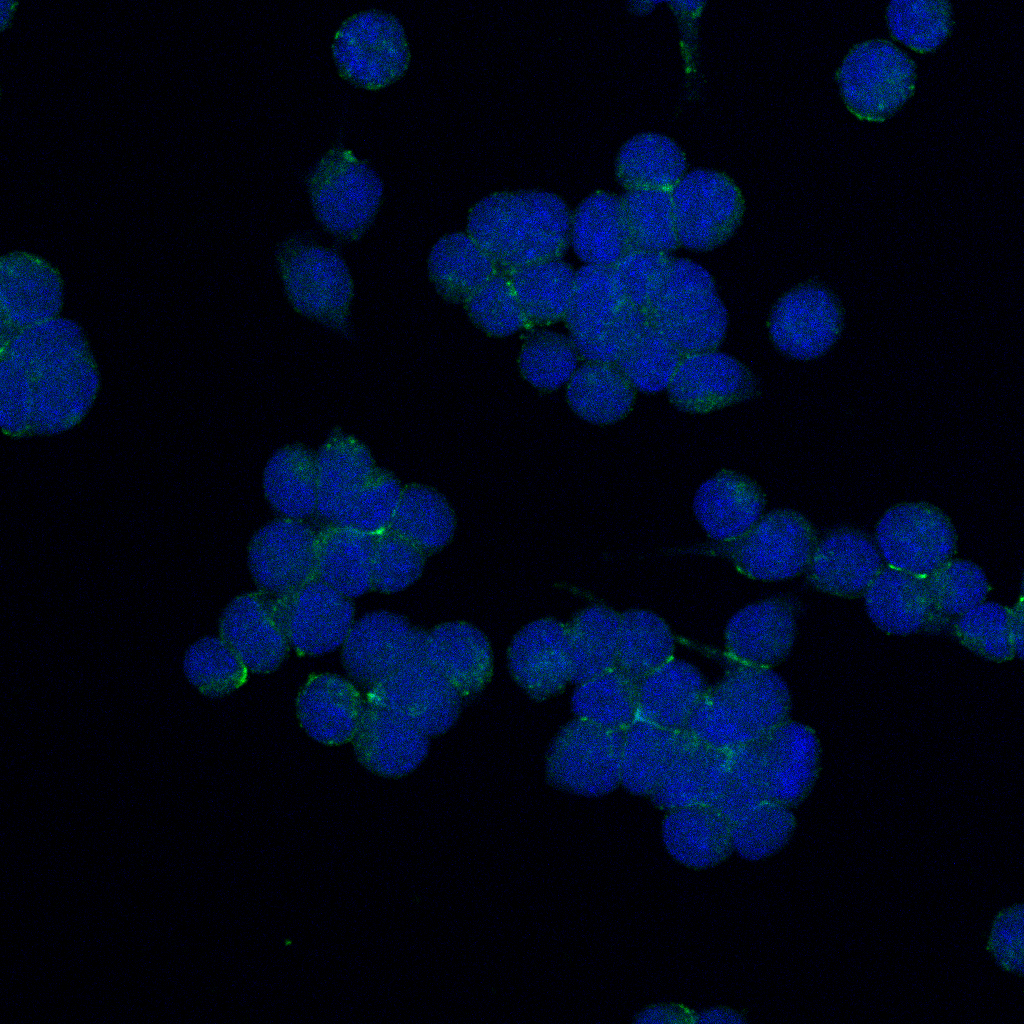

Supplement: Supplementary file 1 [file diseases-13-00060-s001.zip › source data-IF/all IF raw data/WTLPS/WT LPS 1 600-3.tif]

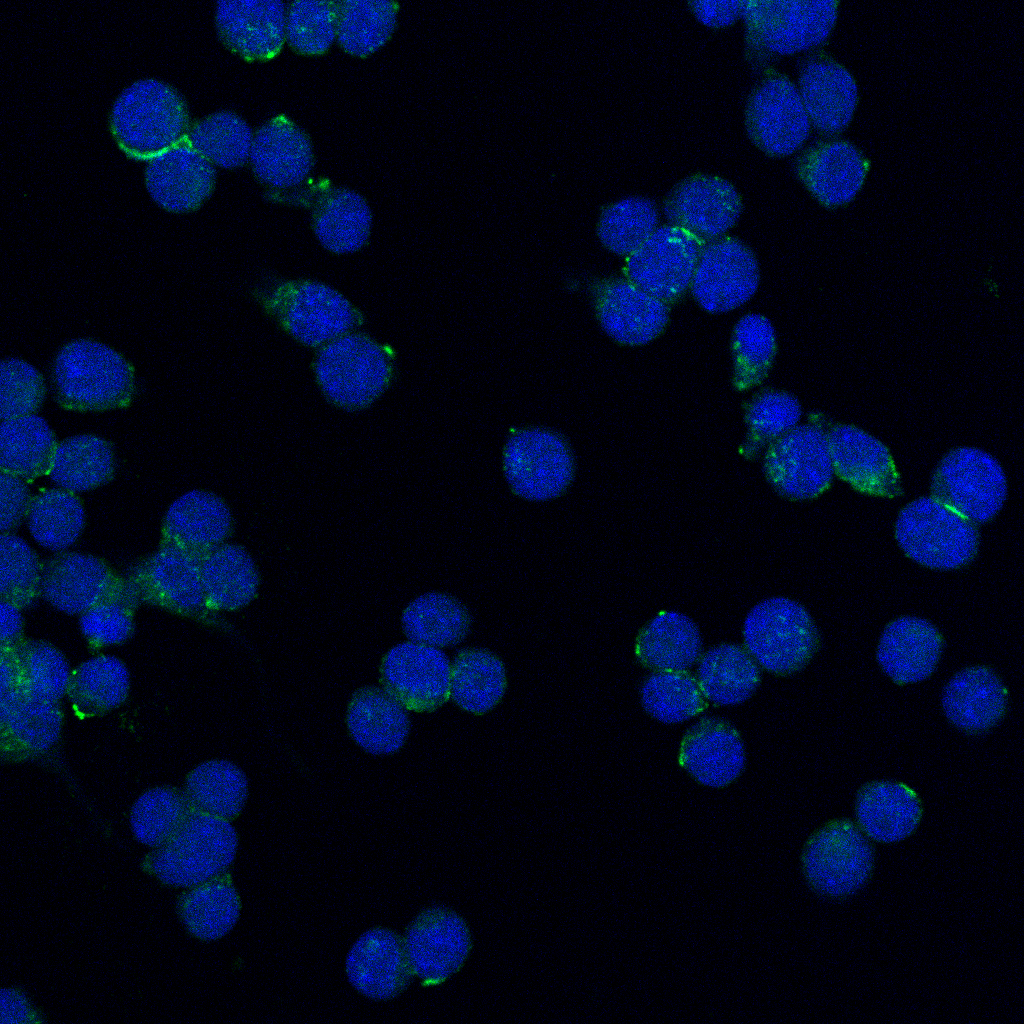

Supplement: Supplementary file 1 [file diseases-13-00060-s001.zip › source data-IF/all IF raw data/WTLPS/WT LPS 1 600-4.tif]

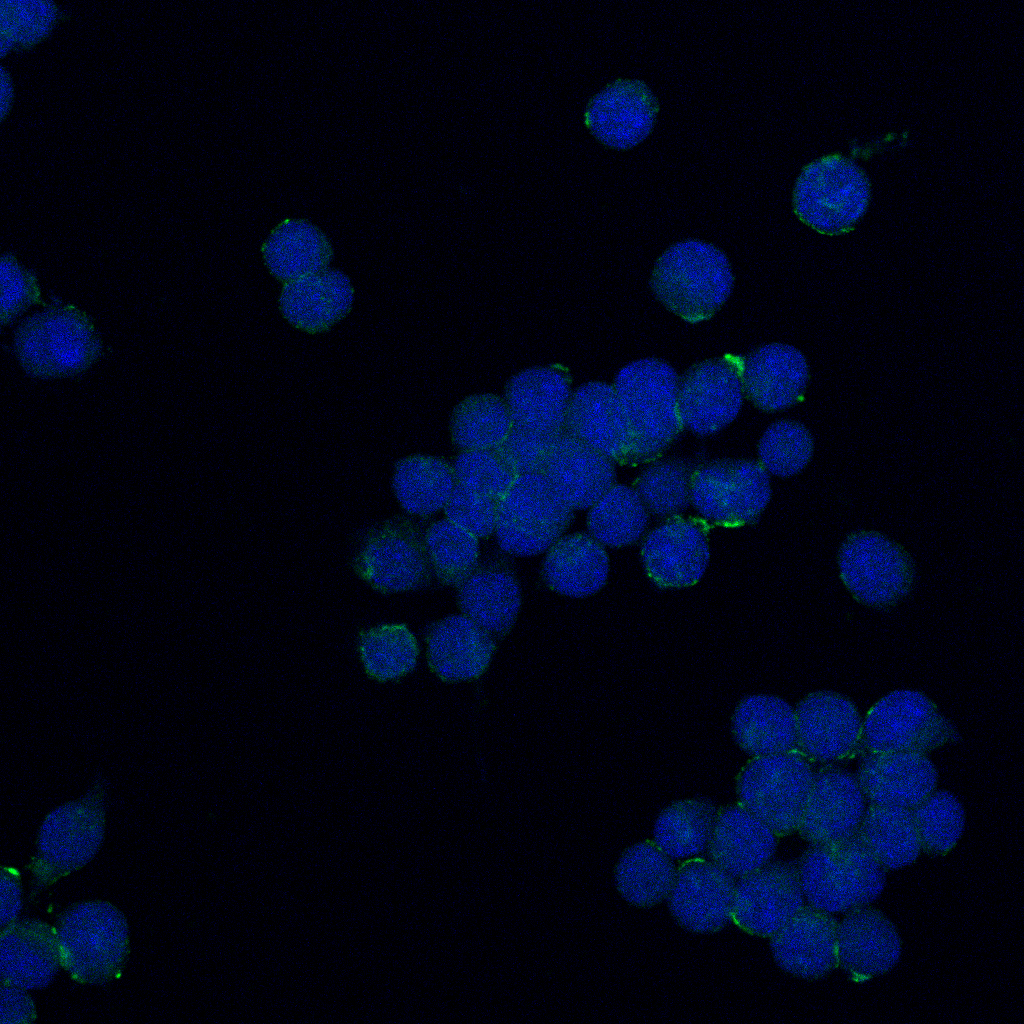

Supplement: Supplementary file 1 [file diseases-13-00060-s001.zip › source data-IF/all IF raw data/WTLPS/WT LPS 1 600-5.tif]

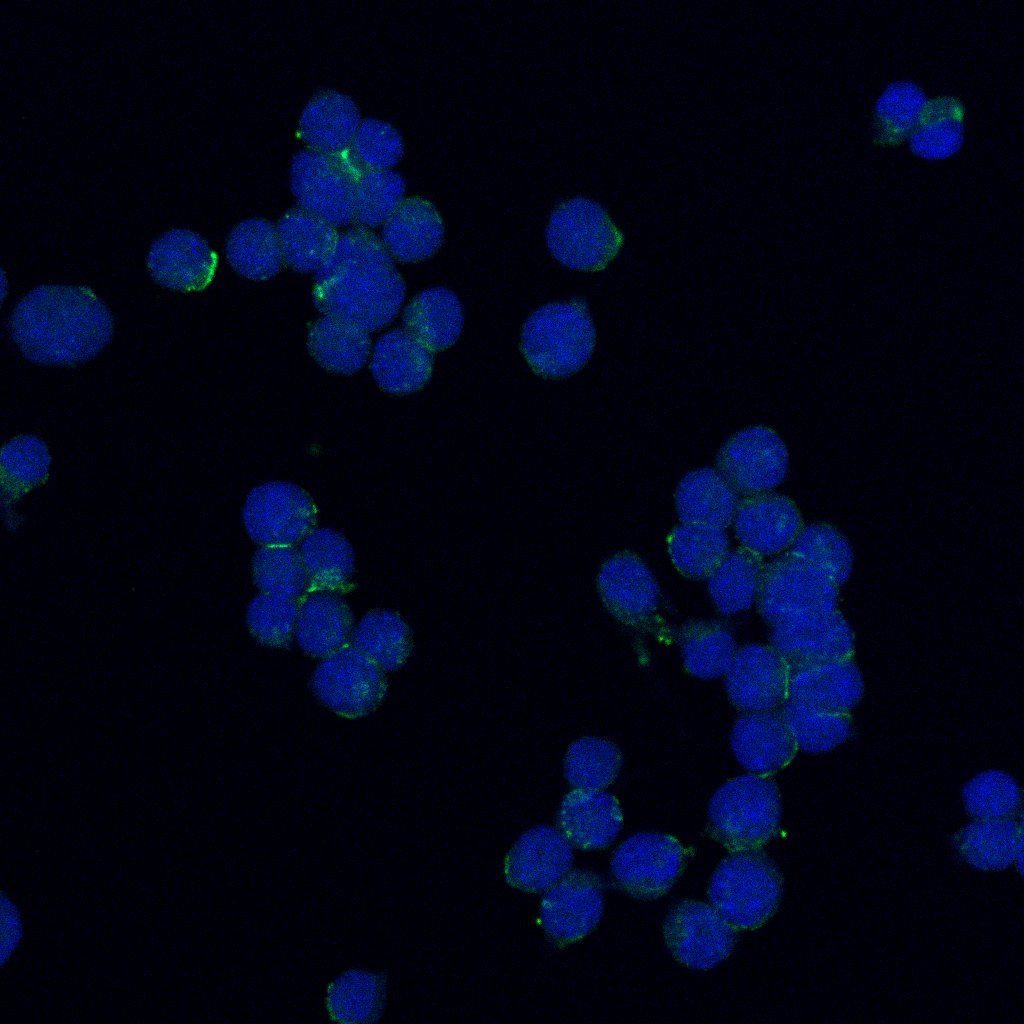

Supplement: Supplementary file 1 [file diseases-13-00060-s001.zip › source data-IF/all IF raw data/WTLPS/WT LPS 1 600-6.tif]

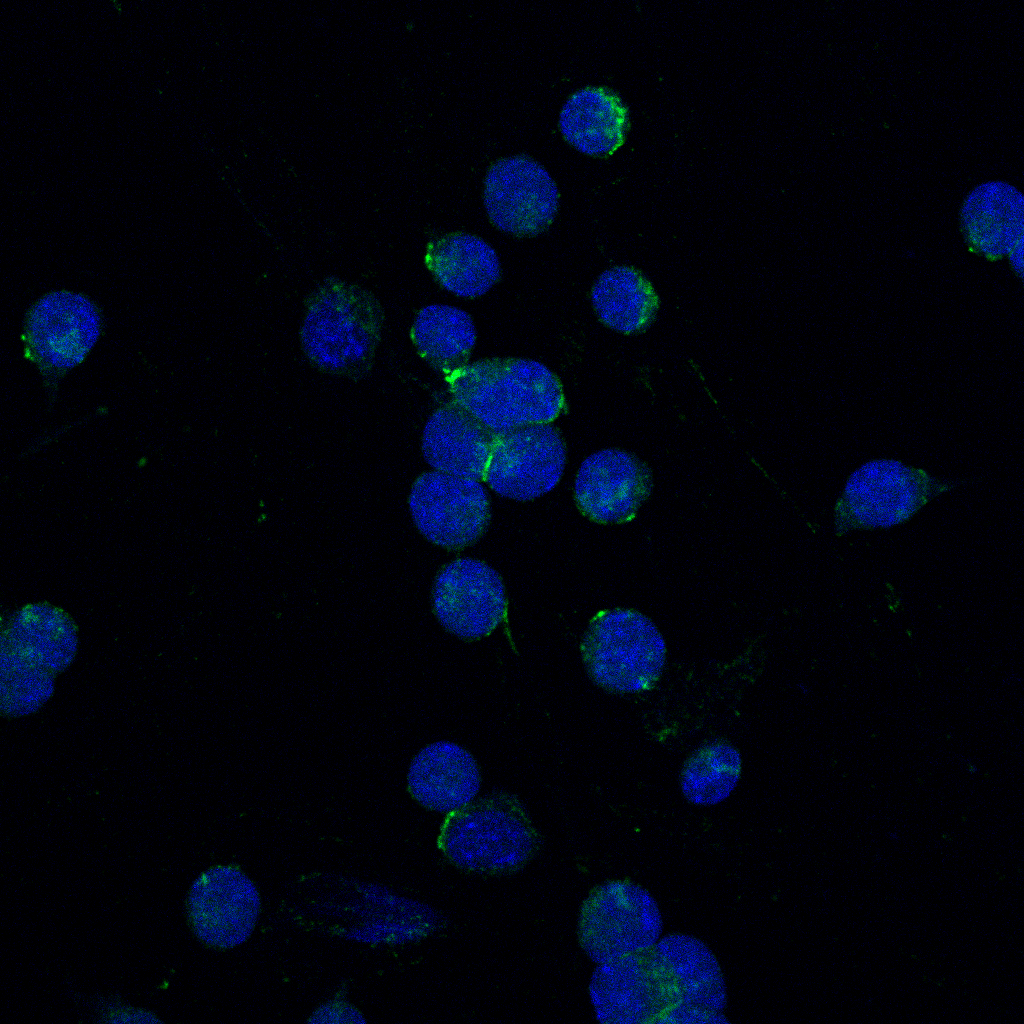

Supplement: Supplementary file 1 [file diseases-13-00060-s001.zip › source data-IF/all IF raw data/WTLPS/WT LPS 2 600-1.tif]

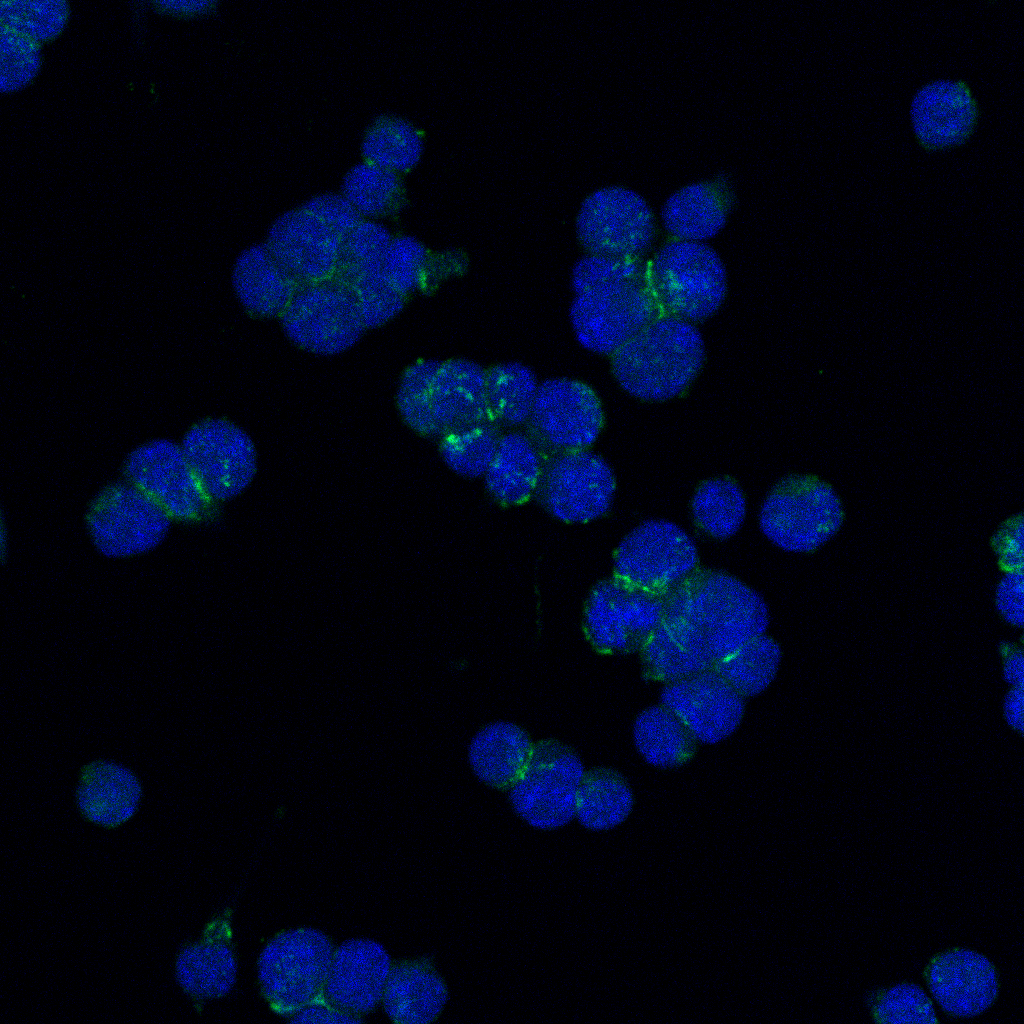

Supplement: Supplementary file 1 [file diseases-13-00060-s001.zip › source data-IF/all IF raw data/WTLPS/WT LPS 2 600-2.tif]

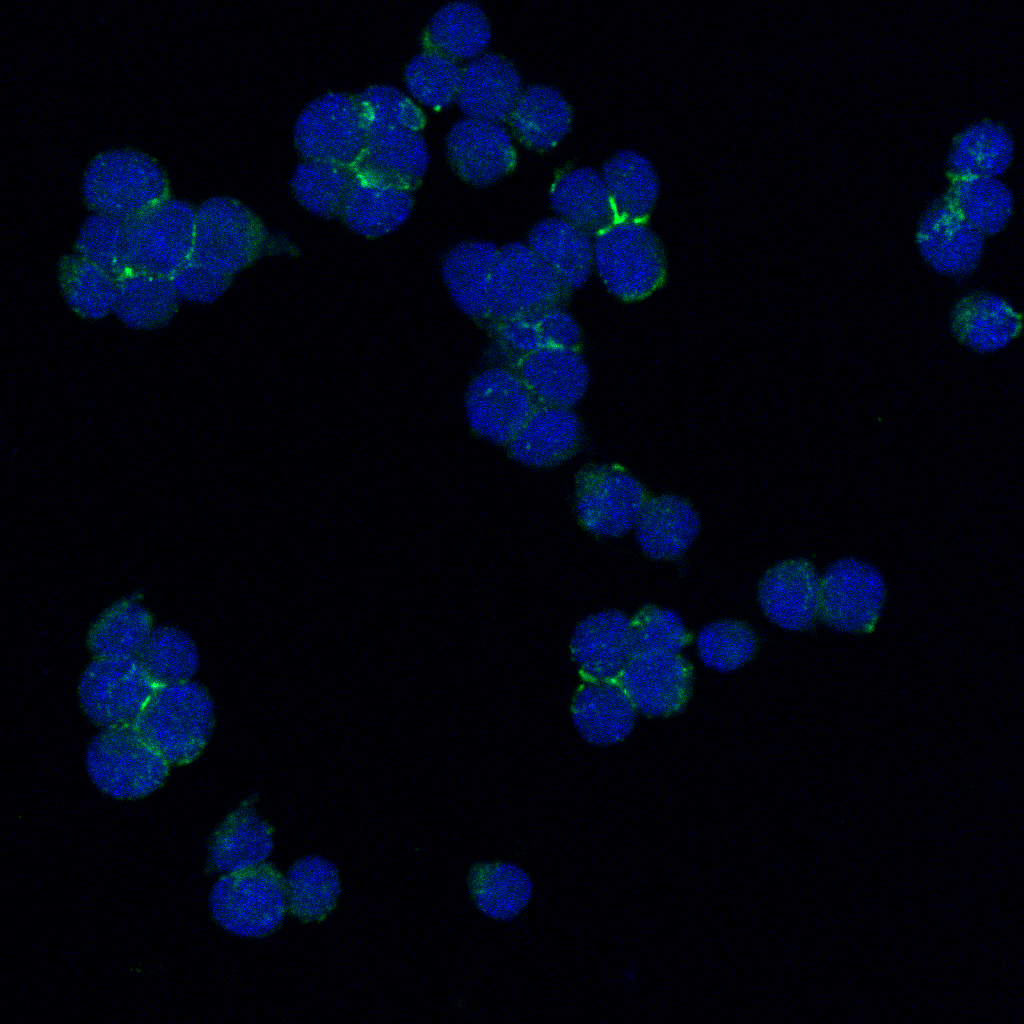

Supplement: Supplementary file 1 [file diseases-13-00060-s001.zip › source data-IF/all IF raw data/WTLPS/WT LPS 2 600-3.tif]

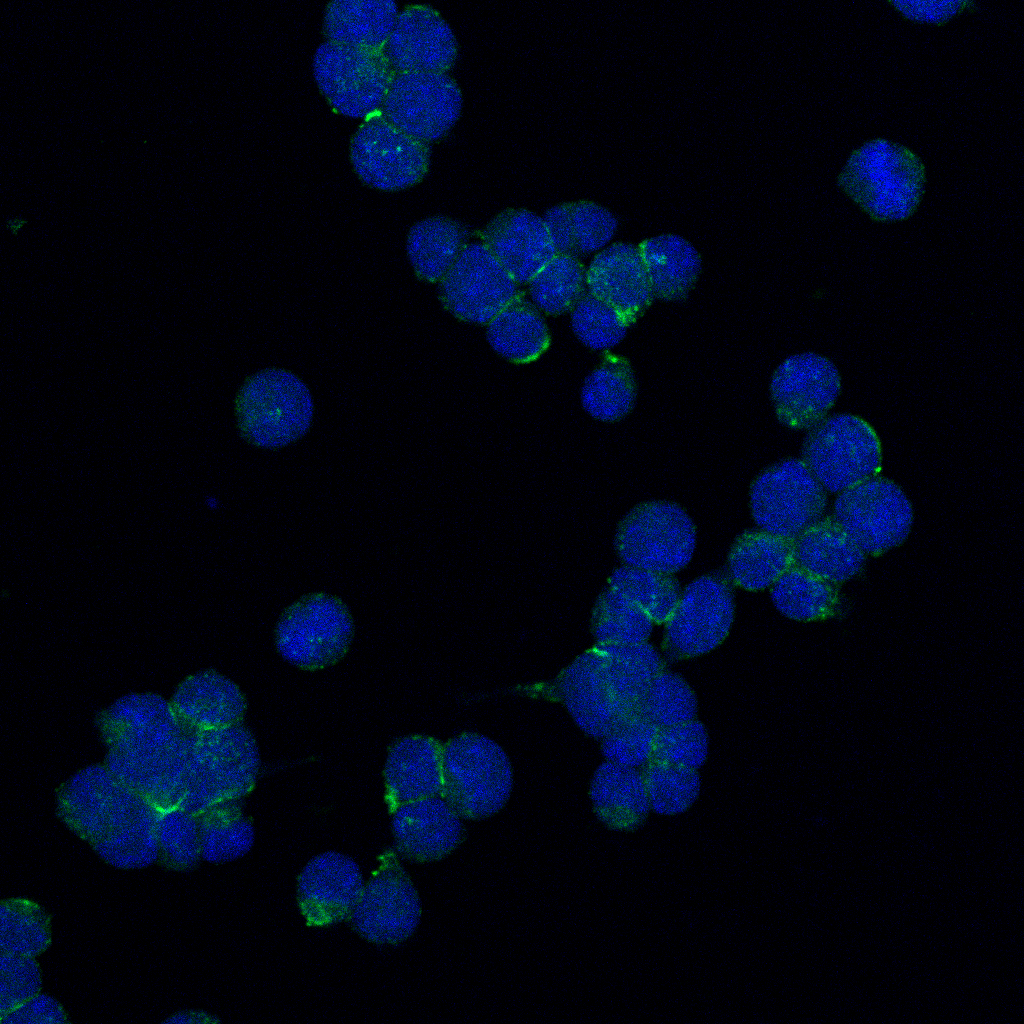

Supplement: Supplementary file 1 [file diseases-13-00060-s001.zip › source data-IF/all IF raw data/WTLPS/WT LPS 2 600-4.tif]

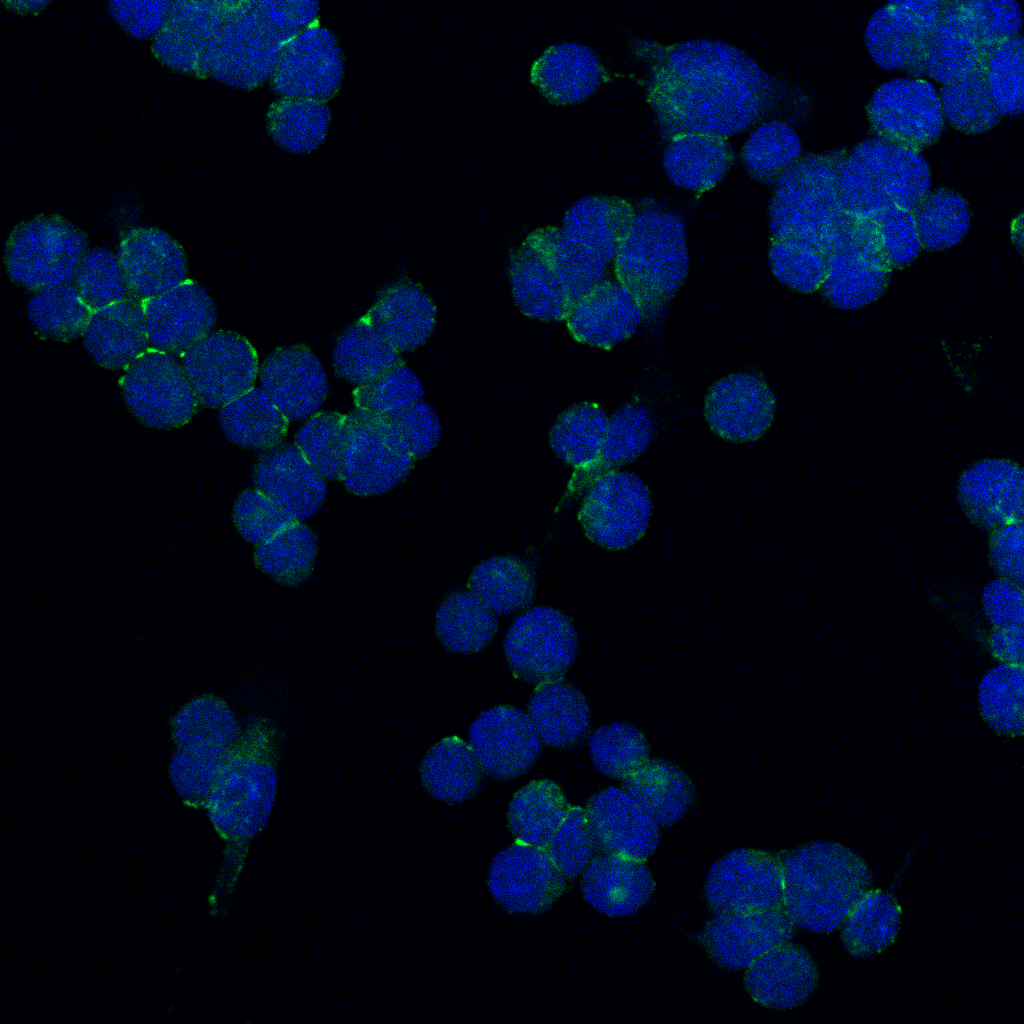

Supplement: Supplementary file 1 [file diseases-13-00060-s001.zip › source data-IF/all IF raw data/WTLPS/WT LPS 2 600-5.tif]

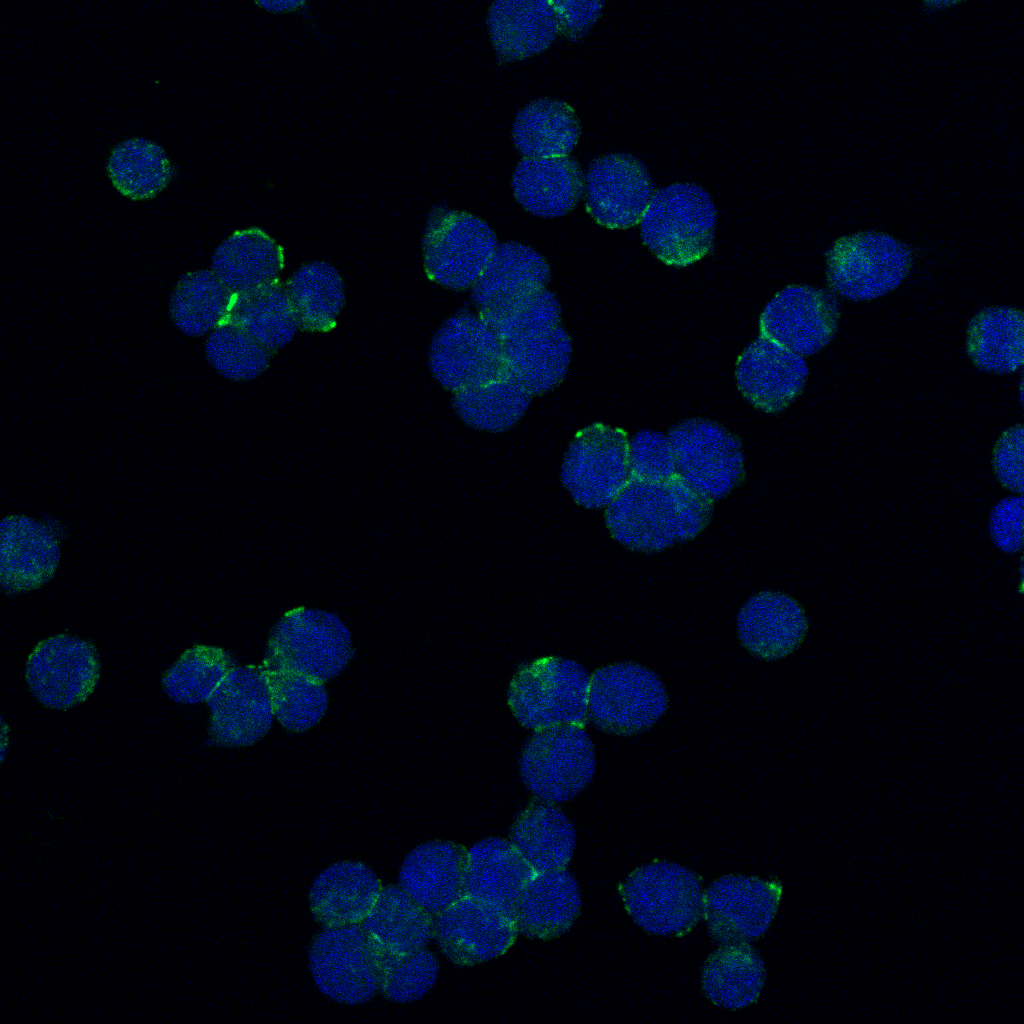

Supplement: Supplementary file 1 [file diseases-13-00060-s001.zip › source data-IF/all IF raw data/WTLPS/WT LPS 2 600-6.tif]

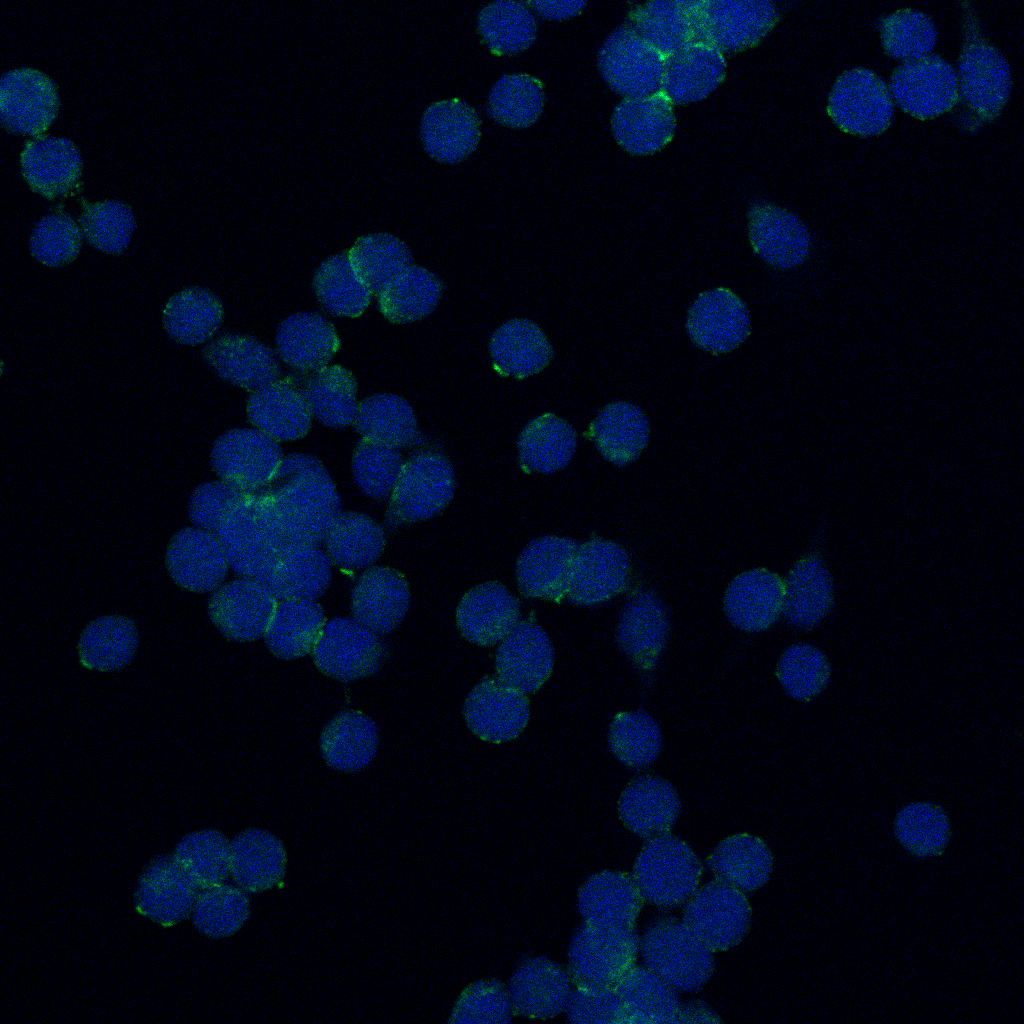

Supplement: Supplementary file 1 [file diseases-13-00060-s001.zip › source data-IF/all IF raw data/WTLPS/WT LPS 3 600-1.tif]

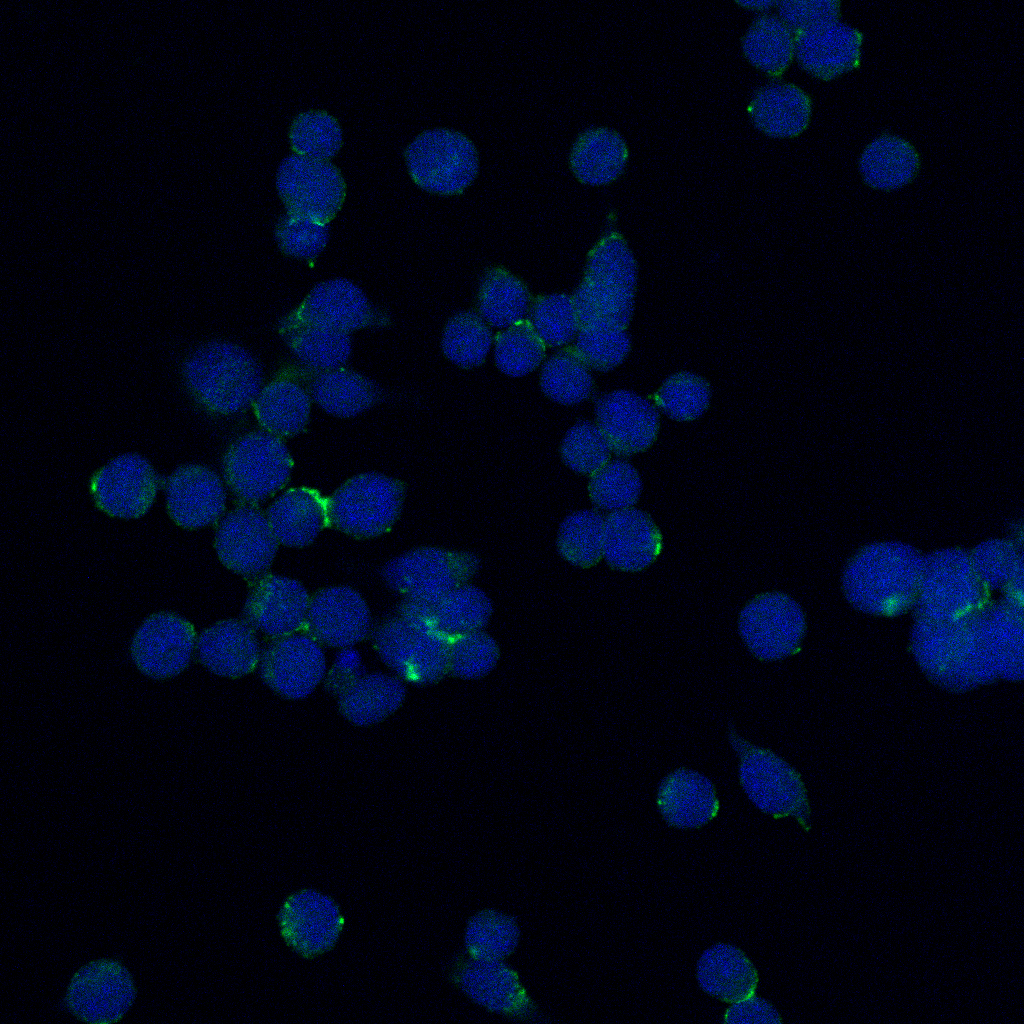

Supplement: Supplementary file 1 [file diseases-13-00060-s001.zip › source data-IF/all IF raw data/WTLPS/WT LPS 3 600-2.tif]

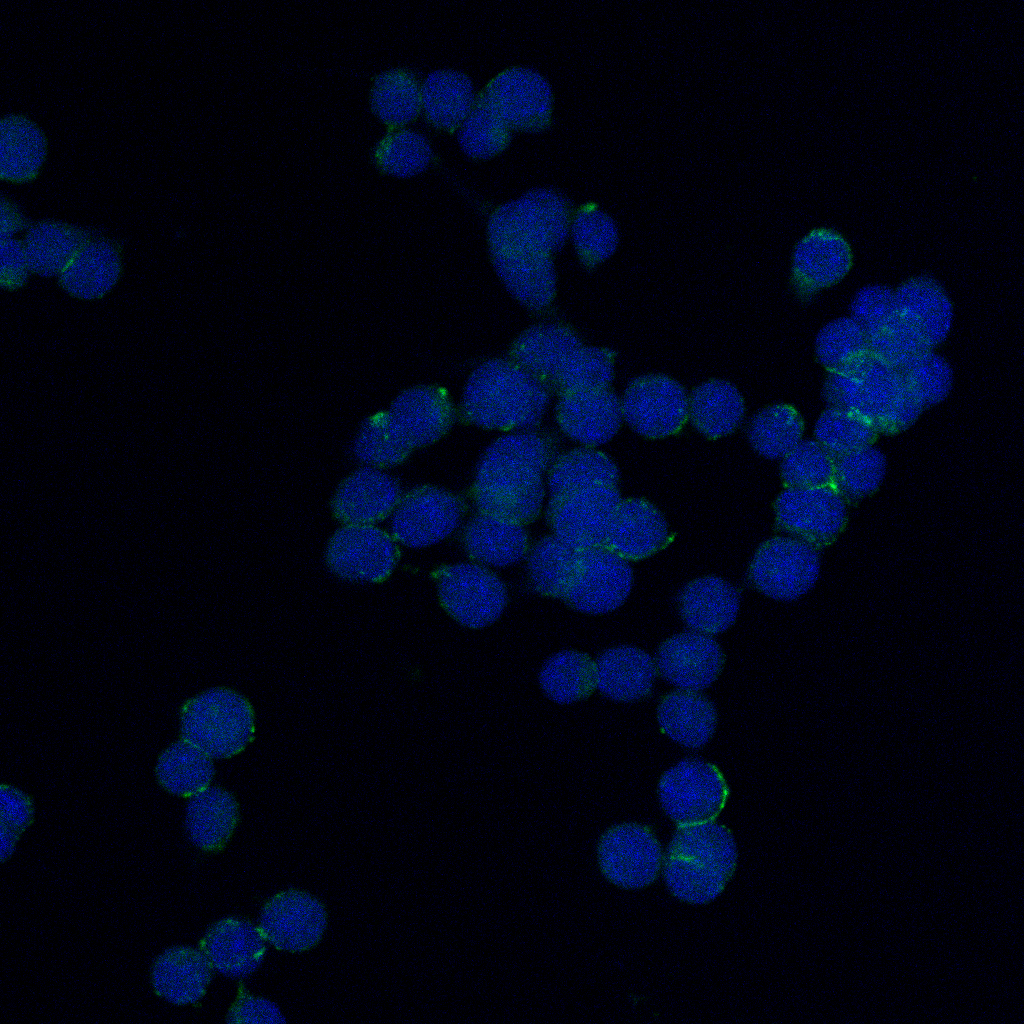

Supplement: Supplementary file 1 [file diseases-13-00060-s001.zip › source data-IF/all IF raw data/WTLPS/WT LPS 3 600-3.tif]

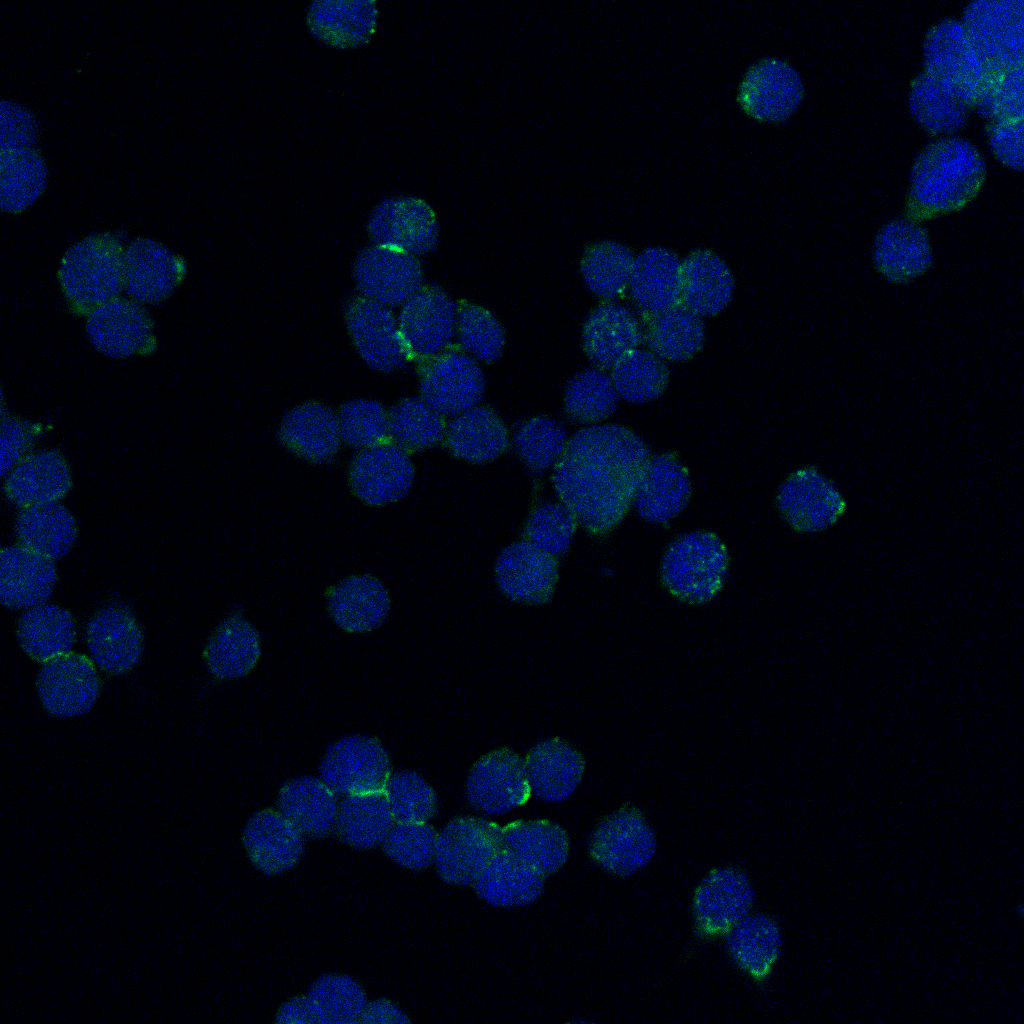

Supplement: Supplementary file 1 [file diseases-13-00060-s001.zip › source data-IF/all IF raw data/WTLPS/WT LPS 3 600-4.tif]

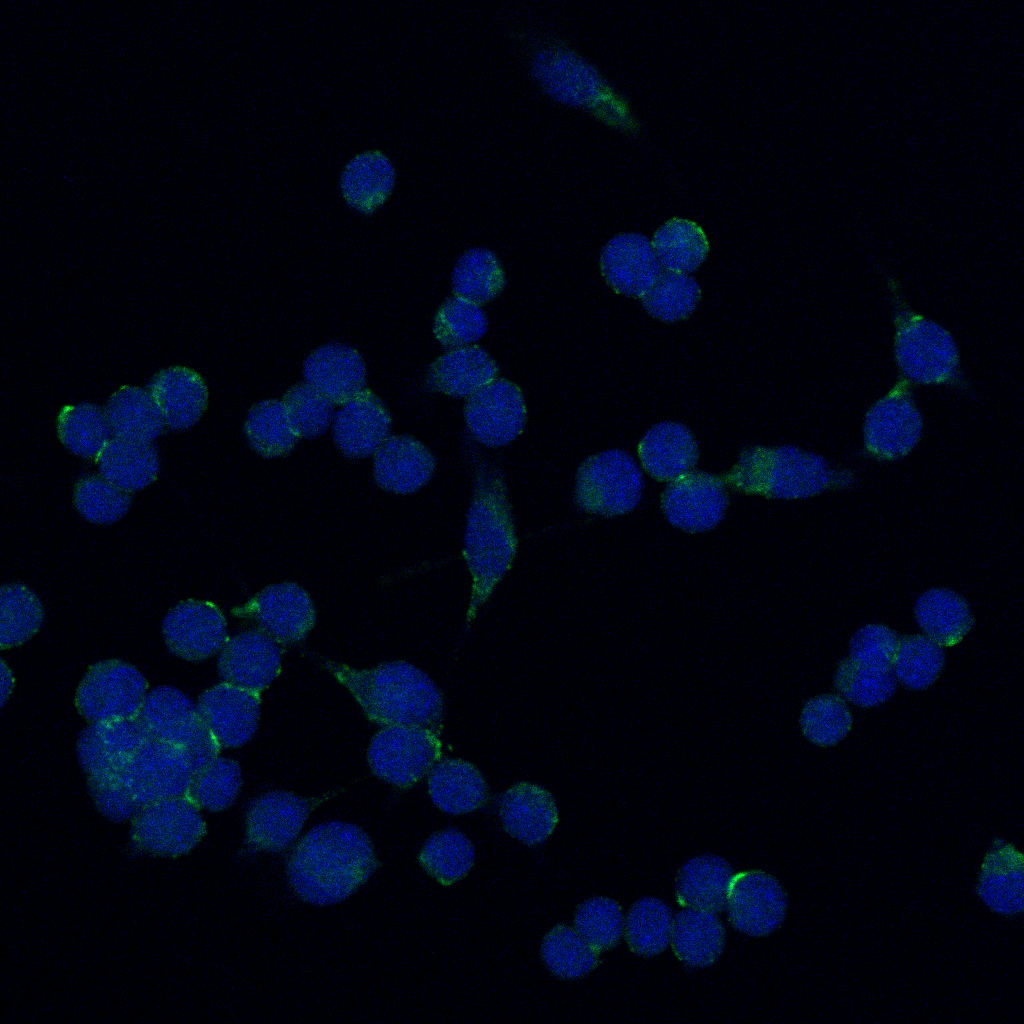

Supplement: Supplementary file 1 [file diseases-13-00060-s001.zip › source data-IF/all IF raw data/WTLPS/WT LPS 3 600-5.tif]

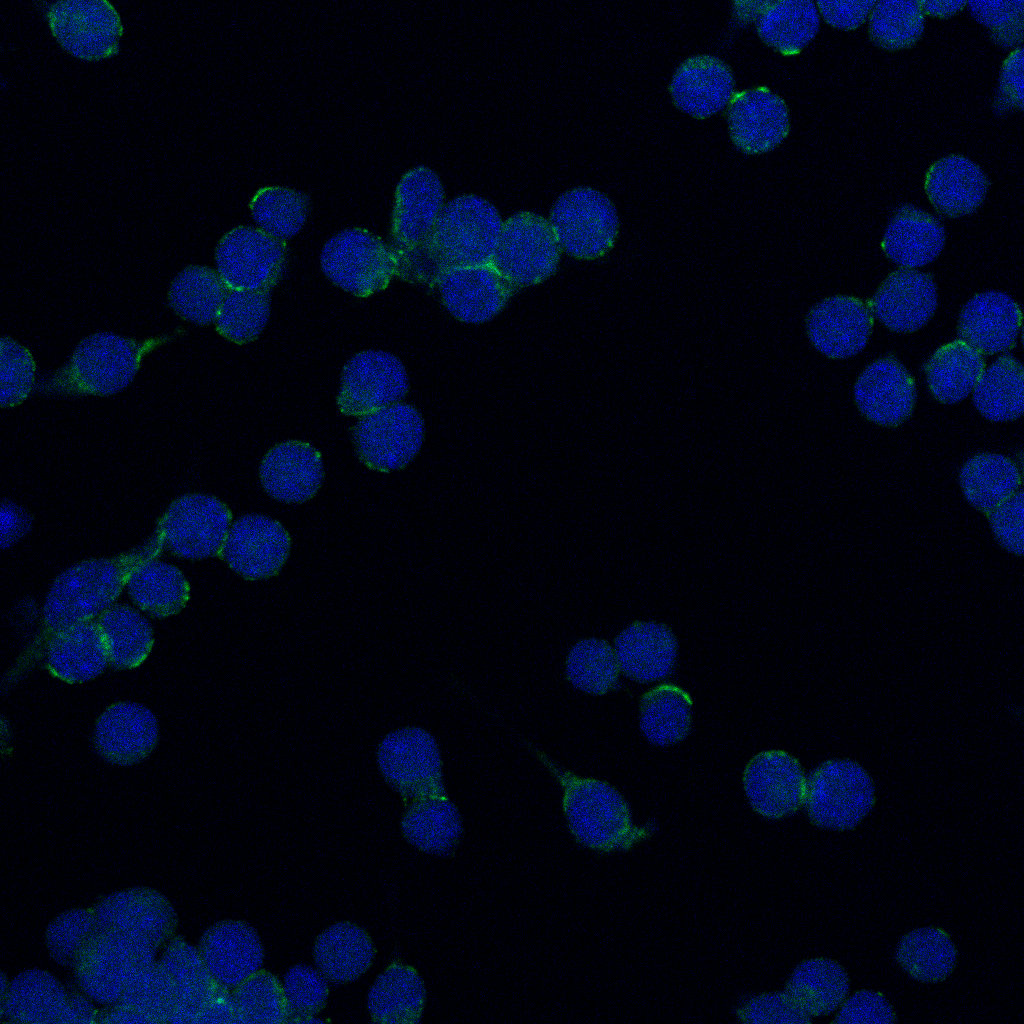

Supplement: Supplementary file 1 [file diseases-13-00060-s001.zip › source data-IF/all IF raw data/WTLPS/WT LPS 3 600-6.tif]

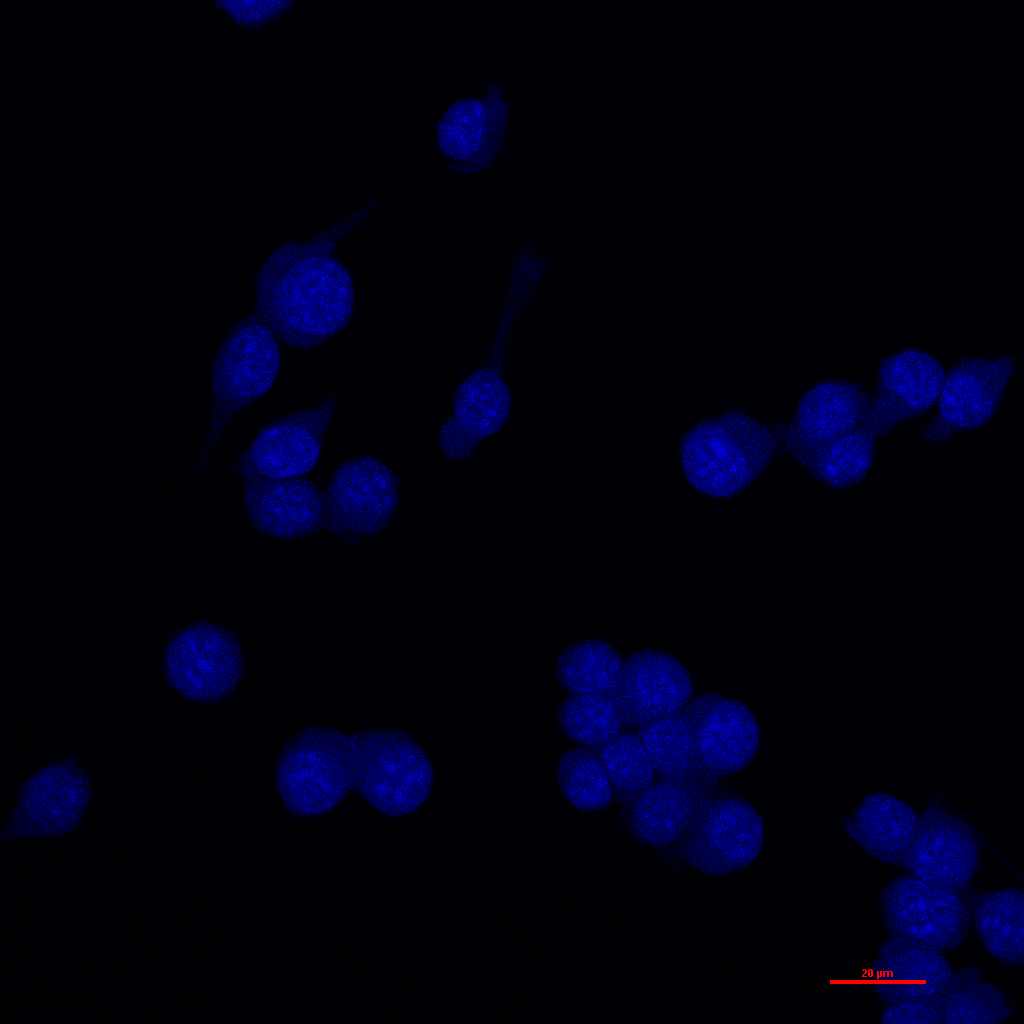

Supplement: Supplementary file 1 [file diseases-13-00060-s001.zip › source data-IF/figure6 shown/OE DAPI.tif]

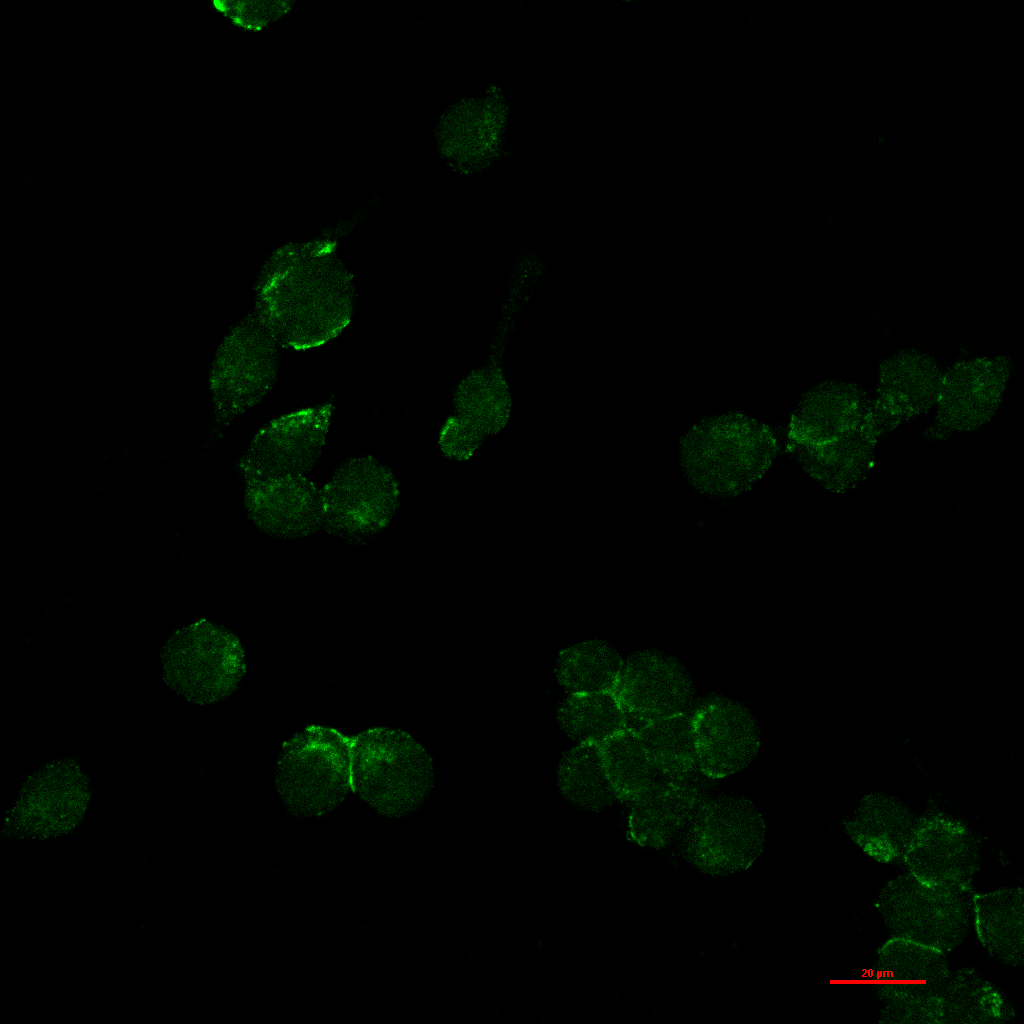

Supplement: Supplementary file 1 [file diseases-13-00060-s001.zip › source data-IF/figure6 shown/OE FITC.tif]

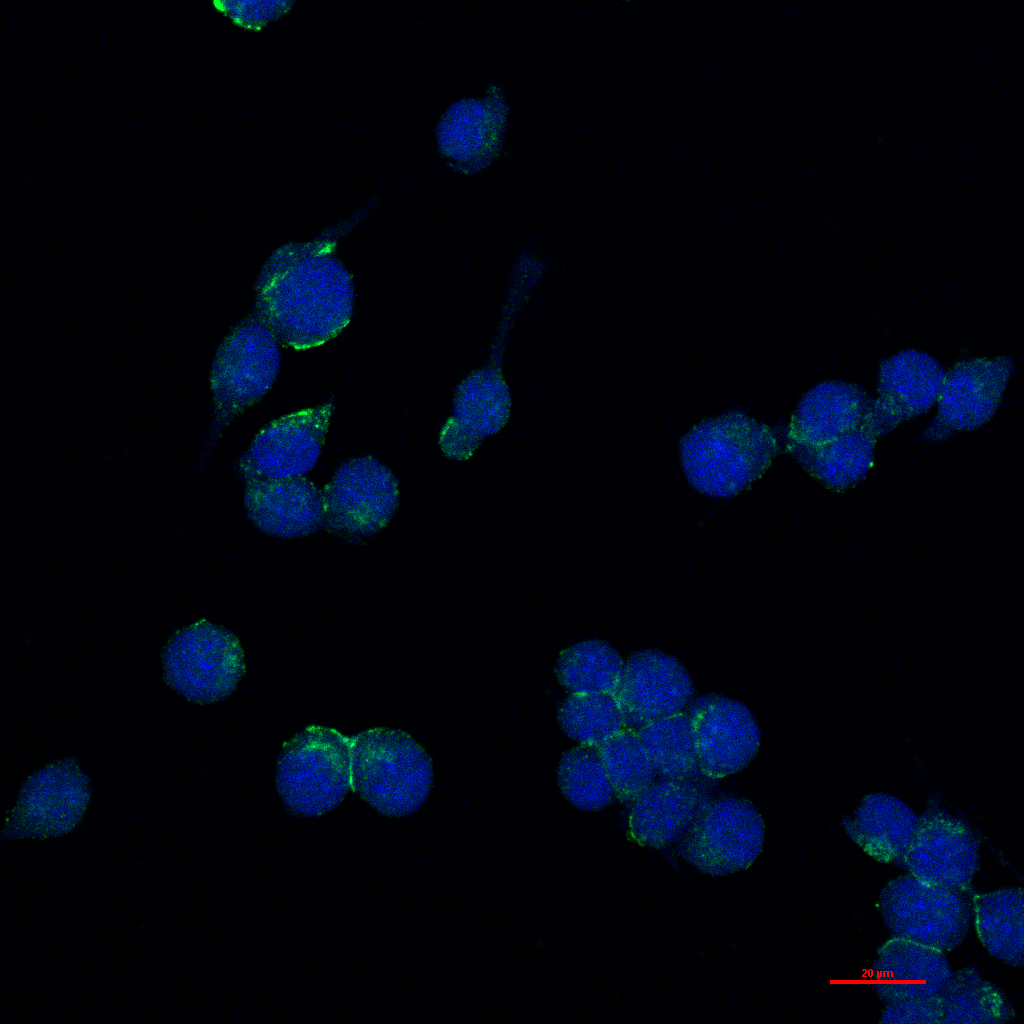

Supplement: Supplementary file 1 [file diseases-13-00060-s001.zip › source data-IF/figure6 shown/OE merge.tif]

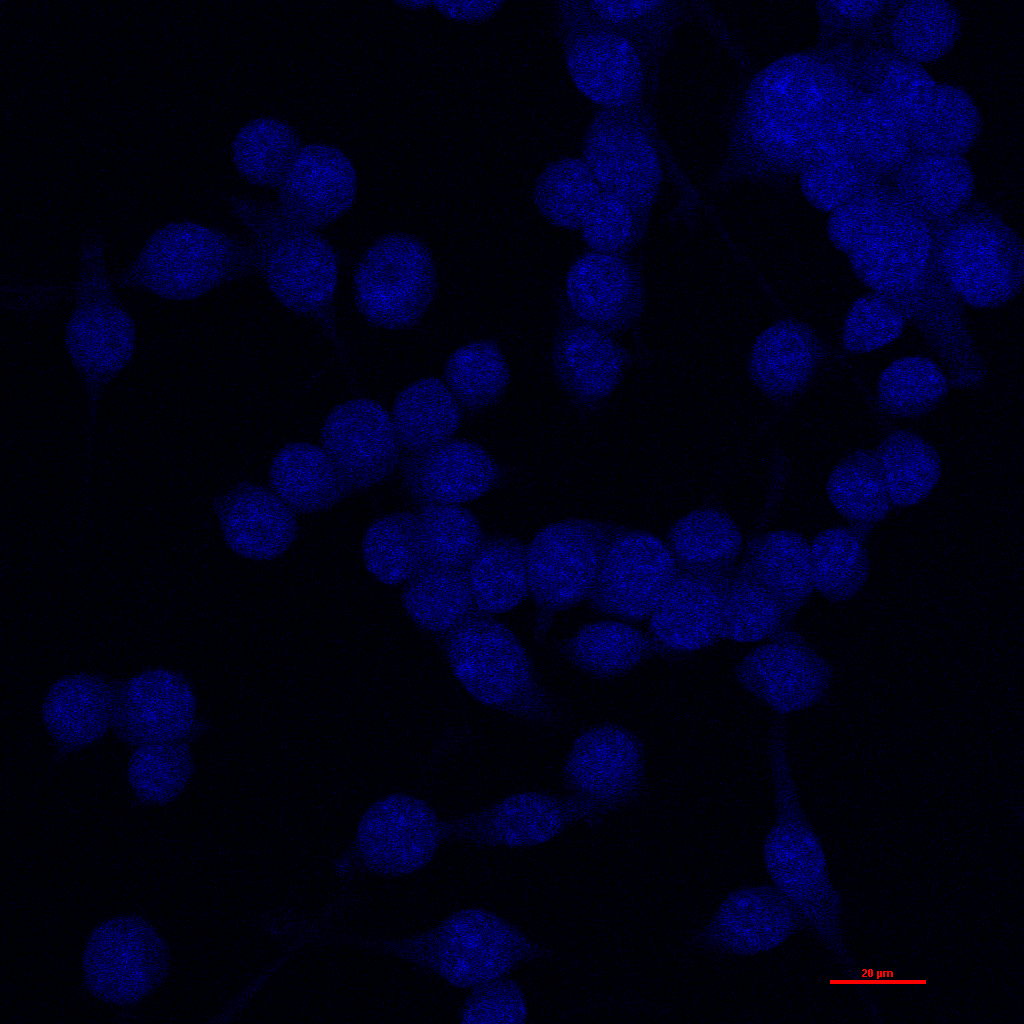

Supplement: Supplementary file 1 [file diseases-13-00060-s001.zip › source data-IF/figure6 shown/OELPS DAPI.tif]

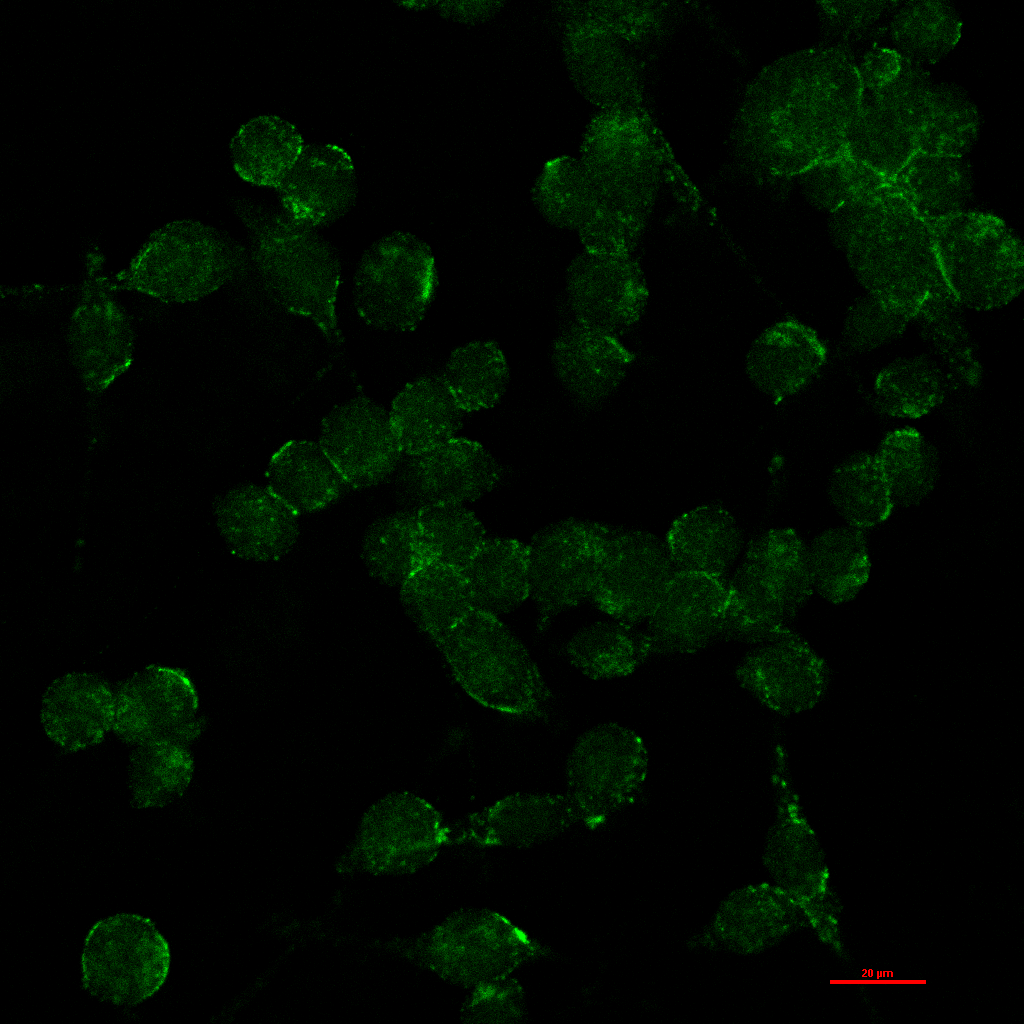

Supplement: Supplementary file 1 [file diseases-13-00060-s001.zip › source data-IF/figure6 shown/OELPS FITC.tif]

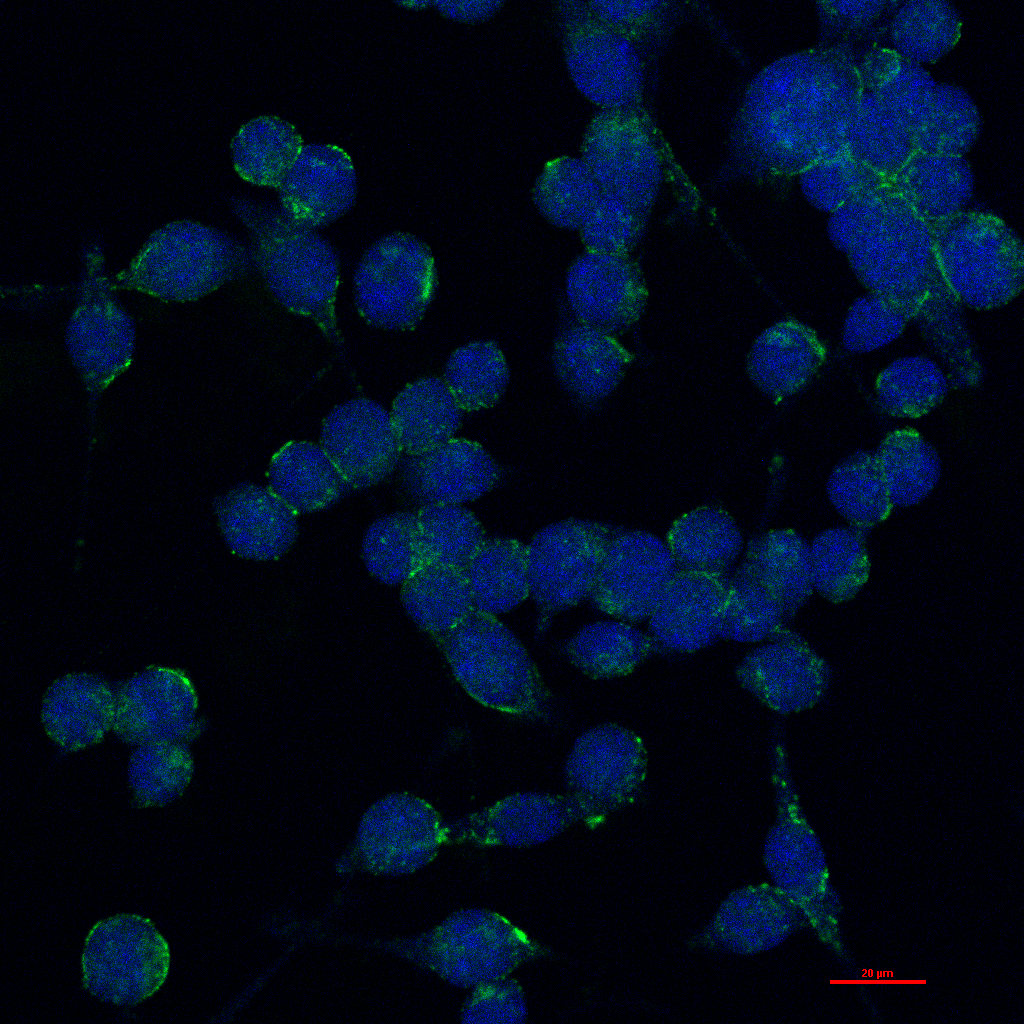

Supplement: Supplementary file 1 [file diseases-13-00060-s001.zip › source data-IF/figure6 shown/OELPS merge.tif]

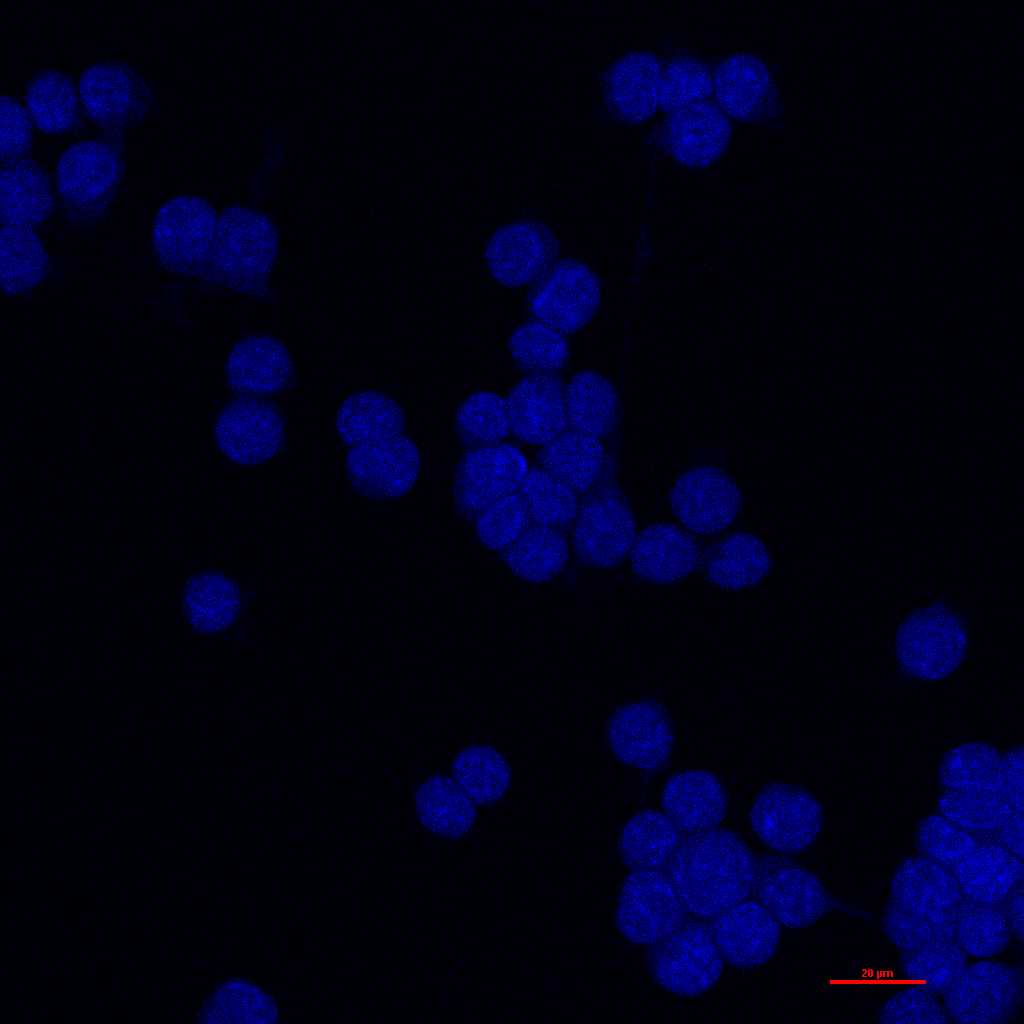

Supplement: Supplementary file 1 [file diseases-13-00060-s001.zip › source data-IF/figure6 shown/WT DAPI.tif]

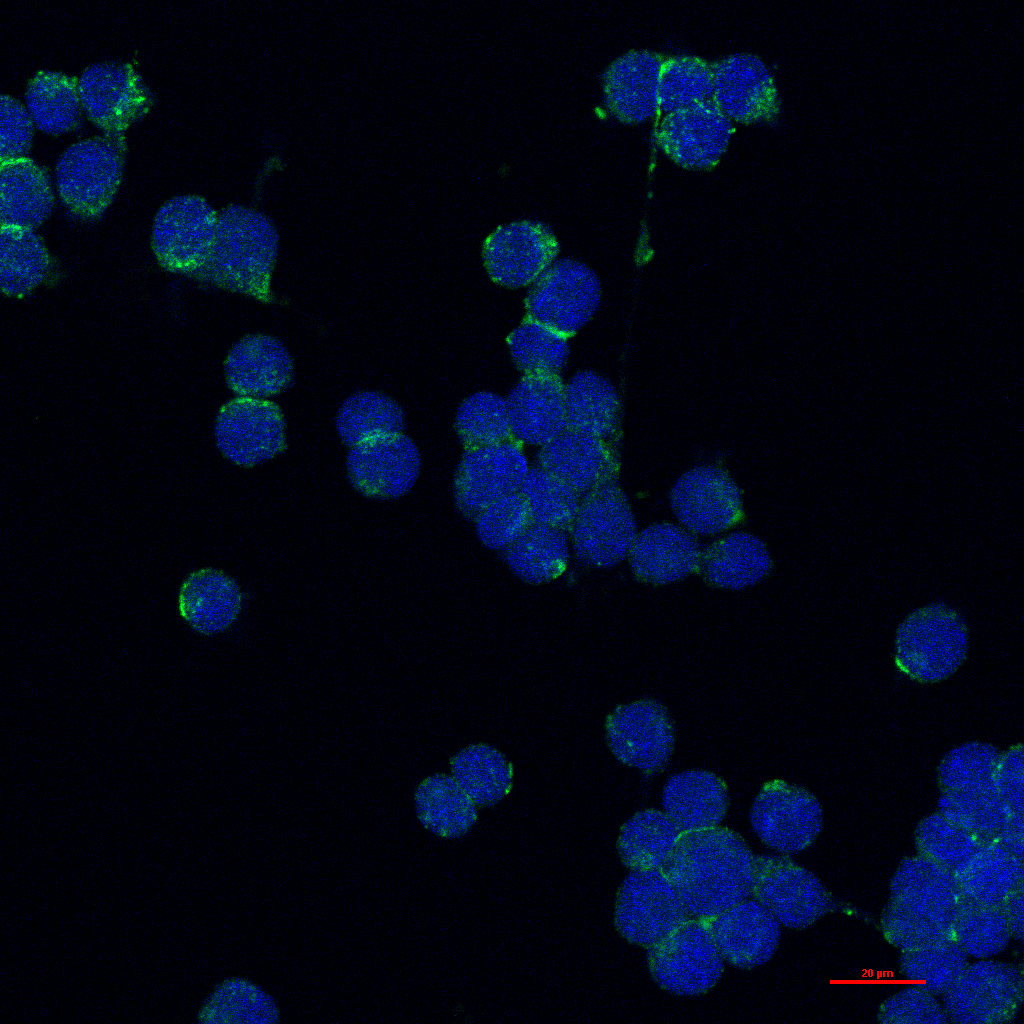

Supplement: Supplementary file 1 [file diseases-13-00060-s001.zip › source data-IF/figure6 shown/WT merge.tif]

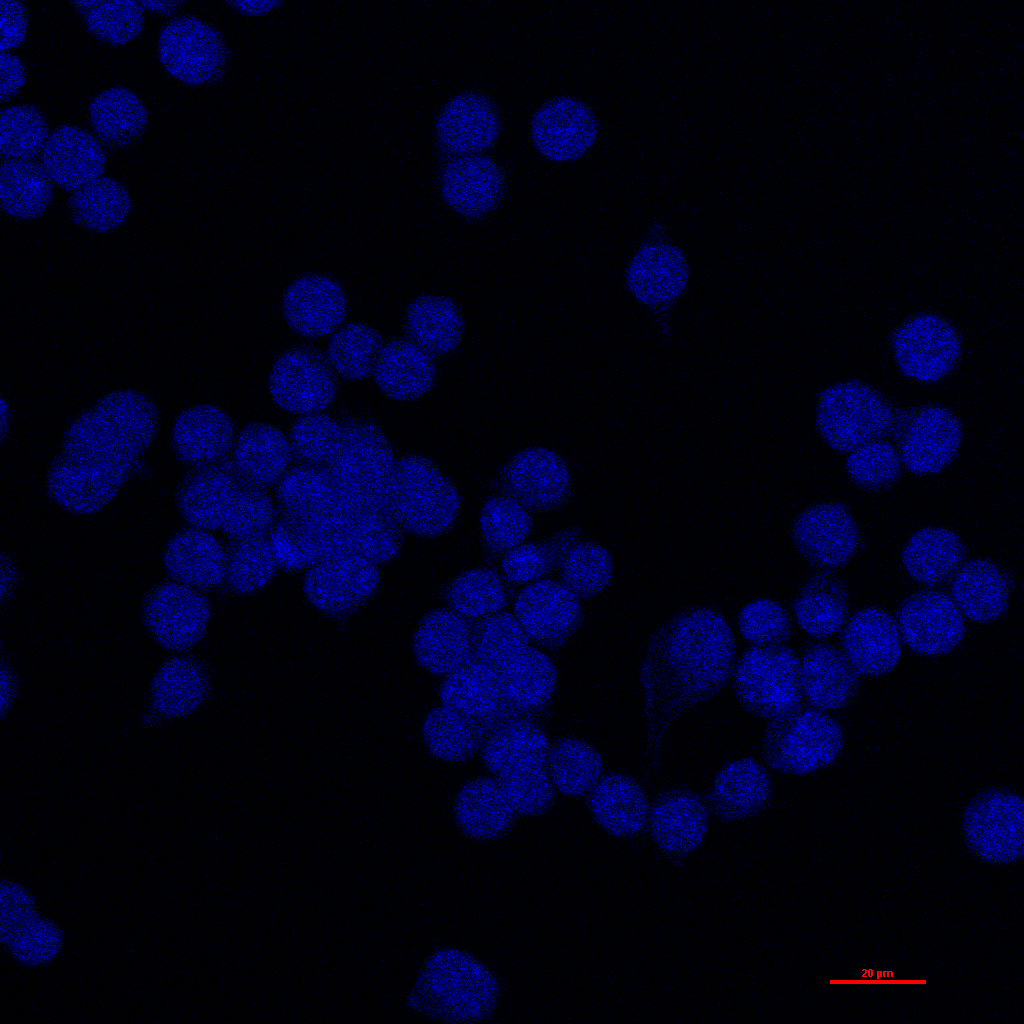

Supplement: Supplementary file 1 [file diseases-13-00060-s001.zip › source data-IF/figure6 shown/WTLPS DAPI.tif]

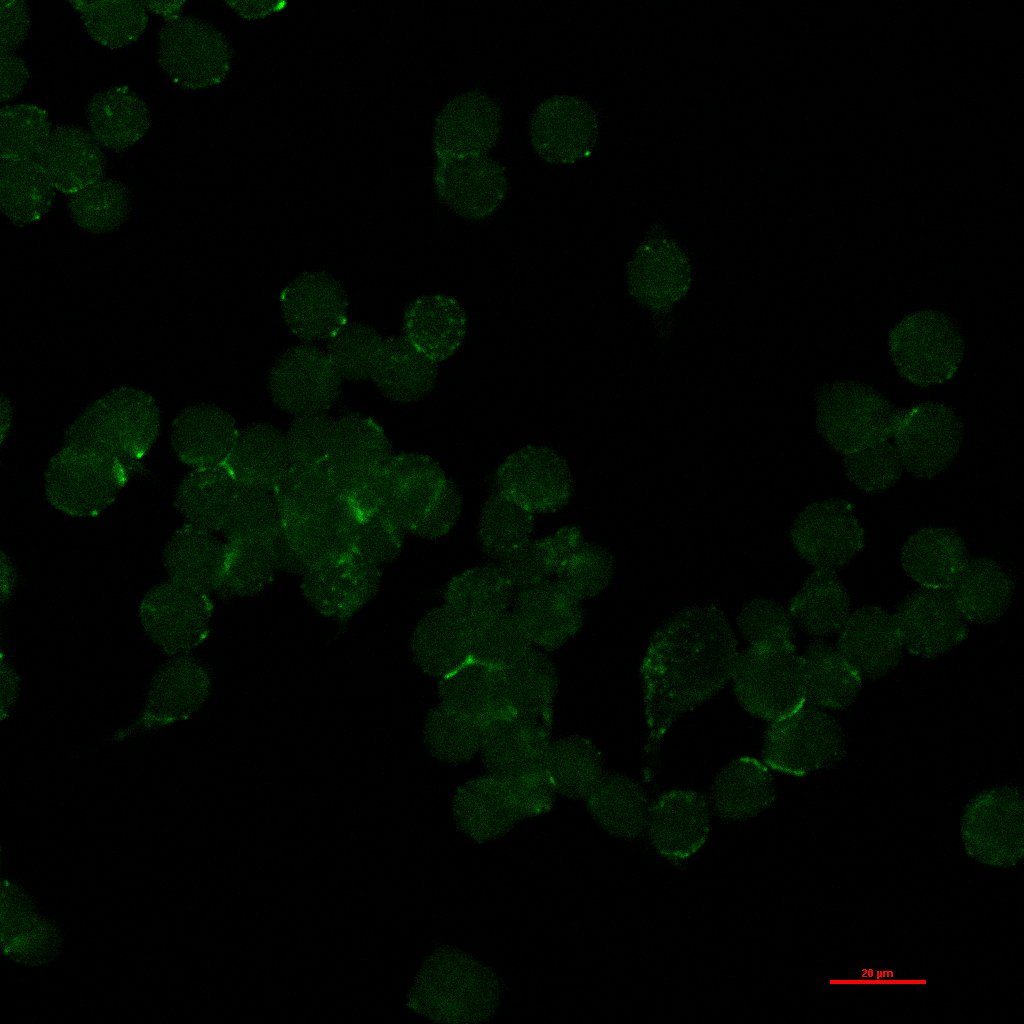

Supplement: Supplementary file 1 [file diseases-13-00060-s001.zip › source data-IF/figure6 shown/WTLPS FITC.tif]

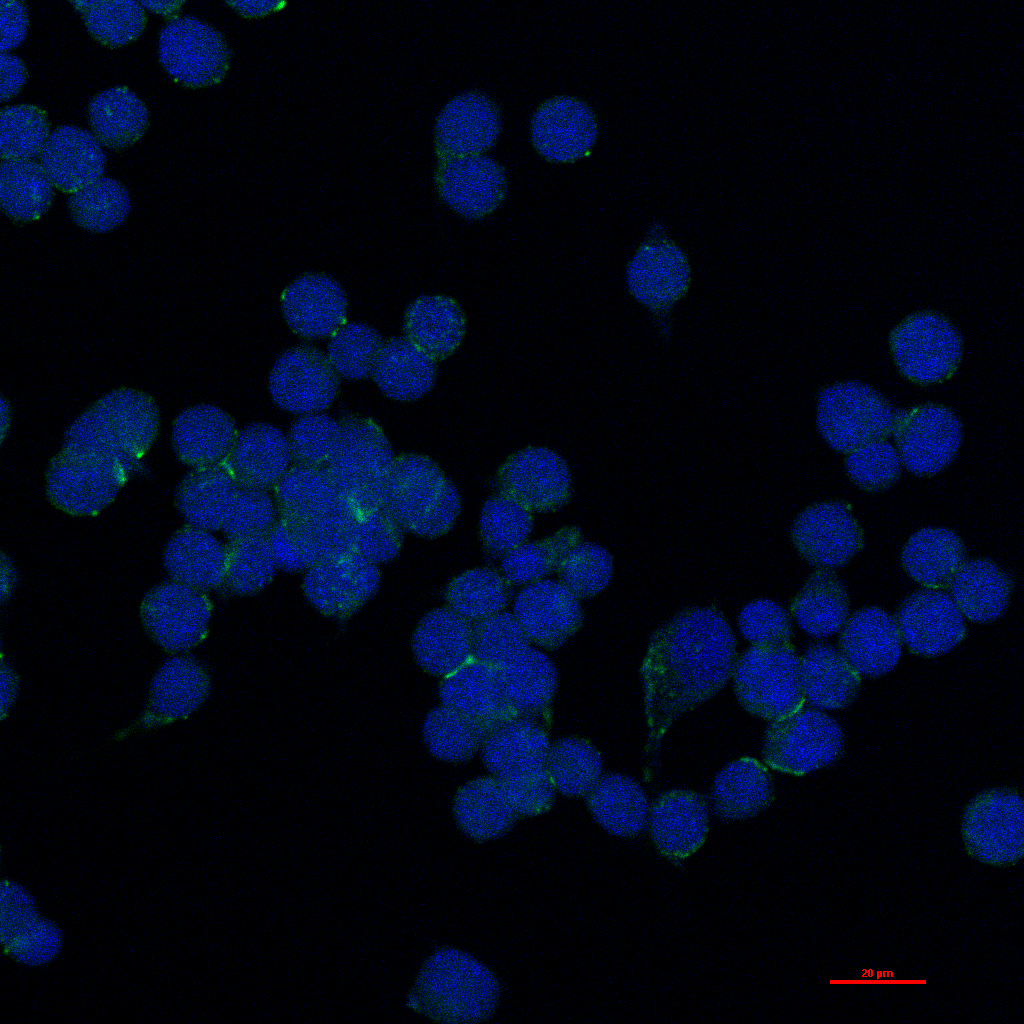

Supplement: Supplementary file 1 [file diseases-13-00060-s001.zip › source data-IF/figure6 shown/WTLPS merge.tif]

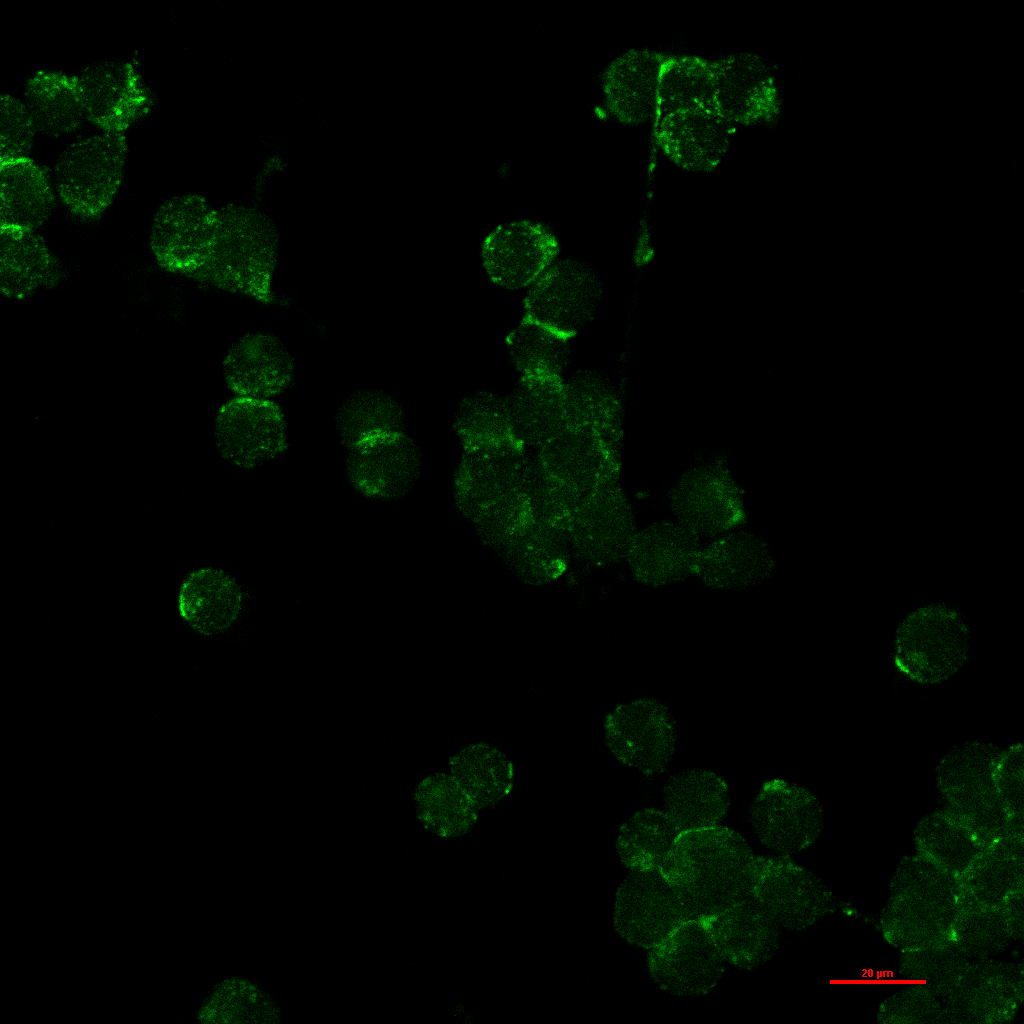

Supplement: Supplementary file 1 [file diseases-13-00060-s001.zip › source data-IF/figure6 shown/WT_FITC.tif]
